# Assessment of Tunisian *Trichoderma* Isolates on Wheat Seed Germination, Seedling Growth and Fusarium Seedling Blight Suppression

**DOI:** 10.3390/microorganisms11061512

**Published:** 2023-06-06

**Authors:** Mouadh Saadaoui, Mohamed Faize, Ludovic Bonhomme, Noura Omri Benyoussef, Mohamed Kharrat, Hatem Chaar, Philippe Label, Jean-Stéphane Venisse

**Affiliations:** 1 Université Clermont Auvergne, INRAE, PIAF, 63000 Clermont-Ferrand, France; mouadh.saadaoui@doctorant.uca.fr (M.S.);; 2 Université de Tunis El Manar, Campus Universitaire Farhat Hached, B.P. n° 94—ROMMANA, Tunis 1068, Tunisia; 3 Field Crops Laboratory, National Institute for Agricultural Research of Tunisia (INRAT), Hedi Karray Street, El Menzah, Ariana 1004, Tunisia; 4 Laboratory of Plant Biotechnology, Ecology and Ecosystem Valorization URL-CNRST 10, Faculty of Sciences, University Chouaib Doukkali, El Jadida 24000, Morocco; 5 UMR 1095 Génétique Diversité Ecophysiologie des Céréales, INRAE, Université Clermont Auvergne, 63000 Clermont-Ferrand, France; 6 National Institute of Agronomy of Tunisia (INAT), Tunis 1082, Tunisia

**Keywords:** biostimulation, biofertilizer, biocontrol, plant defenses, phytohormones, VOCs, *Fusarium culmorum*

## Abstract

Beneficial microorganisms, including members of the *Trichoderma* genus, are known for their ability to promote plant growth and disease resistance, as well as being alternatives to synthetic inputs in agriculture. In this study, 111 *Trichoderma* strains were isolated from the rhizospheric soil of Florence Aurore, an ancient wheat variety that was cultivated in an organic farming system in Tunisia. A preliminary ITS analysis allowed us to cluster these 111 isolates into three main groups, *T. harzianum* (74 isolates), *T. lixii* (16 isolates) and *T.* sp. (21 isolates), represented by six different species. Their multi-locus analysis (tef1, translation elongation factor 1; rpb2, RNA polymerase B) identified three *T. afroharzianum*, one *T. lixii*, one *T. atrobrunneum* and one *T. lentinulae* species. These six new strains were selected to determine their suitability as plant growth promoters (PGP) and biocontrol agents (BCA) against Fusarium seedling blight disease (FSB) in wheat caused by *Fusarium culmorum*. All of the strains exhibited PGP abilities correlated to ammonia and indole-like compound production. In terms of biocontrol activity, all of the strains inhibited the development of *F. culmorum* in vitro, which is linked to the production of lytic enzymes, as well as diffusible and volatile organic compounds. An in planta assay was carried out on the seeds of a Tunisian modern wheat variety (Khiar) by coating them with *Trichoderma*. A significant increase in biomass was observed, which is associated with increased chlorophyll and nitrogen. An FSB bioprotective effect was confirmed for all strains (with *Th01* being the most effective) by suppressing morbid symptoms in germinated seeds and seedlings, as well as by limiting *F. culmorum* aggressiveness on overall plant growth. Plant transcriptome analysis revealed that the isolates triggered several SA- and JA-dependent defense-encoding genes involved in *F. culmorum* resistance in the roots and leaves of three-week-old seedlings. This finding makes these strains very promising in promoting growth and controlling FSB disease in modern wheat varieties.

## 1. Introduction

Organic and chemical-free farming methods to control biotic pathogens and enhance yield are increasing in popularity and practice currently due to the growing concern about the potential negative impact of synthetic chemicals used in conventional farming. This improved yield has been accompanied by modern breeding practices for half of the 20th century in order to select the most effective crop genotypes that take full advantage of farming inputs, thus limiting any potential added value coming from beneficial plant–microbe interactions [1,2]. Due to this, there is an urgent need to develop promising new ways to increase both crop performance and tolerance to biotic and abiotic factors without harming human health or the environment.

Worldwide, wheat is one of the major crops, providing food for about 35% of the world’s population and making up 17% of the global cultivated area [3]. In Tunisia, cereals and their derivatives constitute the base of all diets and the main source of calories. Tunisia’s average rate of wheat consumption per capita is 184 kg per year, making Tunisia one of the largest wheat consumers in the world, a rate which is expected to remain at the same level in the coming years [4,5].

Cereals are susceptible to fungal diseases such as Fusarium seedling blight (FSB) caused by *Fusarium* fungi, including *Fusarium culmorum*, the dominant pathogen in both Tunisia and the Mediterranean region [6]. FSB is responsible for extensive damage to growing seedlings; it can cause significant yield losses and impact the quality of the harvested grain resulting in reduced market value [7]. This disease is most severe in cool and wet conditions, which are common in the winter in Tunisia [8]. Symptoms of FSB include stunted seedlings, yellow leaves and root rot, as well as infected seeds which fail to germinate or die soon after germination. In some severe cases, the entire seedling can collapse and rot [7,9]. *F. culmorum* is a soil-borne fungus that can persist in the soil as chlamydospores for many years, and it can also survive in stubble residues of cereals and other grasses as hyphae [8,10]. This ability makes *F. culmorum* difficult to manage and has major implications for designing effective strategies for disease management. *F. culmorum* is capable of causing both FSB and Fusarium Head Blight (FHB) in wheat. FHB disease occurs in the spikes and is characterized by visible symptoms, such as reddish and scabby spikes, which can result in the accumulation of mycotoxins in the grains [11,12]. Control of FSB has received less attention than control of Fusarium head blight (FHB) because of the risk of mycotoxin contaminations of grain, even though FSB could provide a pathogen source for subsequent epidemics of FHB [12,13]. Control of FSB could be achieved by chemical, cultural and biological strategies [14,15]. Using pesticides as a seed treatment could be problematic because of the toxic residues associated with fungicides [16,17]. Some cultural practices may help reduce the fungal population in the soil, such as deep plowing or removing crop residues, but the effectiveness of these measures is limited due to the persistence of the pathogen in the soil [18]. Another management strategy, crop rotation, is not generally practiced in Tunisia because the market has always had a significant influence on the diversity of crops grown in the country. The aim of increasing food self-sufficiency has led to the promotion of certain crops; durum wheat, for example, is a dominant crop taking approximately 60% of the cereal growing area [19]. In addition, control of FSB has been challenging both due to the lack of fully resistant wheat cultivars and because Tunisia is now considered a hot spot for climate change [20].

The use of beneficial microorganisms which are isolated from soil or plant tissues to control plant pathogens and promote plant growth seems to be a very promising and cost-effective component of an integrated disease management plan for sustainable modern agriculture [21].

From this perspective, *Trichoderma*-based products are a segment that is growing in popularity in the agriculture industry, as *Trichoderma* spp. can help improve soil health and plant growth. These products are used for a variety of applications, including soil inoculants, seed treatments, biopesticides and biofertilizers. Indeed, *Trichoderma* spp. are common rhizospheric inhabitants, and the ability of certain species to colonize plant roots allows them to engage with their host in complex molecular dialogues that promote plant growth and productivity [22]. In addition, *Trichoderma* spp. are considered BioControl Agents (BCAs), conferring protection against a wealth of plant pathogens [23]. Multiple molecular mechanisms have been suggested to be responsible for its biocontrol abilities, such as interference with the life cycles of plant pathogens by mycoparasitism (or hyperparasitism); using the secretion of antibiotics (volatile organic compounds, VOCs) and extracellular cell wall-degrading enzymes (CDWEs); competition for nutrients and space; chemical modification of the environmental conditions; and modulation of the host’s innate immunity performance [24,25,26,27,28]. The local and systemic elicitations of the plant defensive responses are correlated with a significant increase in disease resistance against a broad spectrum of plant pathogenic fungi, such as various *Fusarium* spp. [29,30,31,32,33,34].

Some *Trichoderma* strains were isolated from Tunisian soil and plant habitats and were studied as beneficial agents in wheat [33,35]. However, the diversity of available beneficial strains is very limited, predominantly focused on some species that primarily belong to one of the five taxonomic sections of the “*Trichoderma harzianum species complex*” (THSC) [36,37,38]. This gap concerns all of the plant species of agronomic interest. In this respect, exploring the greater diversity of *Trichoderma* and evaluating their biocontrol abilities would be of great benefit in minimizing the pathogens spread in wheat. However, the success of a fungal biocontrol agent depends not only on the complex interactions it is able to establish with the plant host and its related pathogens but also on its aptitude to colonize a rhizospheric environment modeled by the crops already present, the soil structure and the remarkable endogenous microbial diversity.

Based on the hypothesis that ancient wheat genotypes could represent a source of beneficial fungi such as *Trichoderma*, we focused our research on Florence Aurore, an ancient wheat variety that was cultivated in an organic farming system in Tunisia, for this study. Our aim was to check this variety for the presence of beneficial *Trichoderma* species in the rhizosphere through isolation, molecular characterization (ITS, *tef1* and *rpb2*) and assessment of their plant growth promotion abilities (with the production of indole-like compounds, hydrogen cyanide, ammonia and lytic enzymes, or the solubilization of phosphate). In addition, the isolated strains were evaluated for their possible biocontrol activity against *F. culmorum*, in the context of Fusarium seedling blight, through dual confrontation assays and assessments of diffusible and volatile compound productions. Finally, to confirm these PGP and biocontrolling features, in planta experiments were conducted in greenhouse conditions on Khiar, one of the most marketed wheat varieties in Tunisia, followed by the quantification of defense-related gene expression in the leaves and roots to assess the potential of the *Trichoderma* isolates as plant immunity inducers. Our data provide a set of new Tunisian *Trichoderma* strains that could contribute to the use of field applications of *Trichoderma* fungus as a biocontrol agent.

## 2. Materials and Methods

### 2.1. Tunisian Sampling Location

Field sampling was conducted in May 2020. The study site was located in “Gousset el bey” in the governorate of Bizerte at 36.924096° N, 9.699089° E, in the northwestern part of Tunisia (Figure S1 in Supplementary Materials). This area is characterized by a warm Mediterranean climate with hot, dry summers and humid and cool winters. The average annual temperature range is 18.4 °C, and the rainfall is around 547 mm per year (https://en.climate-data.org/africa/tunisia/bizerte/bizerte-3551/; accessed on 31 May 2023).

### 2.2. Isolation and Culture Conditions

*Trichoderma* strains were isolated from rhizospheric soil of healthy plants of the wheat variety Florence Aurore cultivated in an organic farming system in Tunisia. Rhizospheric soil samples were randomly collected at grain-filling stage, i.e., Growth Stage 87, according to the code defined by Zadoks et al. [39]. One gram of soil tightly adhering to the roots was incubated in 10 mL of sterile Deshydrate Bouillon Potato Dextrose (Potato Dextrose Broth, PDB) in a test tube and shaken at 200 rpm for 30 min at 28 ± 2 °C. From this starting solution, serial dilutions were prepared at the following concentrations, 10^−1^, 10^−2^ and 10^−3^, and then plated on *Trichoderma*-Selective Medium (TSM) [40]. After 5 days of incubation, all of the new colonies that appeared were purified and maintained on Potato Dextrose Agar (PDA) at 25 °C. Pure cultures were cut into cubes using sterile scalpels, transferred into Eppendorf tubes containing 1 mL of 20% glycerol then stored at −20 °C for later use.

### 2.3. Molecular Identification and Bioinformatic Analyses of Trichoderma spp.

Molecular identification of *Trichoderma* strains was performed from monosporic cultures. Fresh mycelia were frozen in liquid nitrogen and ground to a fine powder with 2 mm diameter metal balls for 90 s. Genomic DNA was extracted using the Cetyltrimethylammonium Bromide method [41]. DNA concentration and purity were determined using a NanoDrop™ Spectrophotometer ND-1000 (NanoDrop, Saint Cyr l’Ecole, France). The three loci, which include the nuclear ribosomal internal transcribed spacer (ITS: ITS1-5.8S-ITS4), the partial translation elongation factor 1-alpha (*tef1*) and the partial second-largest subunit of RNA polymerase II (*rpb2*), were amplified by polymerase chain reaction (PCR). PCR amplifications were carried out using BioRad thermal cyclers, and conditions were as follows: initial denaturation at 95 °C for 2 min, followed by 35 cycles including denaturation at 95 °C for 30 s, annealing for 30 s with the corresponding temperatures (58 °C for ITS, 56 °C for *tef1*, 55 °C for *rpb2*); extension at 72 °C for 45 s, then a final extension at 72 °C for 5 min. PCR reactions were performed in a final volume of 50 μL, comprising 1 μL genomic DNA (25 ng/μL), 10 μL colorless GoTaq Flexi Buffer (5×), 3 μL MgCl_2_ solution (25 mM), 1 μL PCR Nucleotide Mix (10 mM each dNTP), 1 μL of each 5 μM primer, 0.25 μL GoTaq (G2 Flexi DNA Polymerase 5 u/μL) and 32.75 μL sterile water. The ITS primer pair consisted of *ITS1* (5′-TCCGTAGGTGAACCTGCGG) and *ITS4* (5′-TCCTCCGCTTATTGATATGC) [42], the *tef1* primer pair consisted of EF1-728F (5′-CATCGAGAAGTTCGAGAAGG-3′) [43] and TEF1-LLErev (5′-GCCATCCTTGGAGATACCAGC-3′) [44] and the *rpb2* pair consisted of RPB2-5F2 (5′-GGGGWGAYCAGAAGAAGGC-3′) and RPB2-7CR (5′-CCCATRGCTTGYTTRCCCA-3′) [45]. PCR products were analyzed by agarose gel electrophoresis (1.5%) and sequenced by the genomics service company Genewiz. Nucleic sequences were identified using NCBI BLASTn. The newly generated partial ITS, *tef1* and *rpb2* gene sequences of the six *Trichoderma* isolates reported in this study were recorded to NCBI GenBank database. Their accession numbers and those of the related *Trichoderma* strains used in the analyses are listed in Table 1.

The phylogenetic analyses were conducted on the concatenated gene version 5′-ITS (575 bp)/tef1 (1260 bp)/rpb2 (930 bp)-3′, with the reference sequences downloaded from GenBank that correspond to the most related sequences. All of the nucleotide data were weighted equally, and gaps were treated as missing characters. After removing any ambiguously aligned regions, the alignments were 2639 nucleotides. The multiple DNA sequence alignments were performed with the Clustal-W algorithm. The maximum likelihood analysis was employed to construct a phylogenetic tree as implemented in the PhyML program. The topology of the tree was evaluated by bootstrap analysis of the sequence data based on 1000 random resamplings.

### 2.4. Bioassays for Antagonism Traits of Trichoderma spp. against F. culmorum

#### 2.4.1. Plant Pathogen

Virulent isolate of *F. culmorum*, the causal agent of Fusarium seedling blight (FSB), was kindly provided by the crop laboratory (LGC) of the National Tunisian Institute of Agronomic Research (INRAT). Fungus was routinely grown and maintained on PDA medium at 25 °C.

#### 2.4.2. In Vitro Confrontation Assay

The antagonism of *Trichoderma* strains against *F. culmorum* was evaluated with dual culture assays, according to Skidmore and Dickinson [46]. Mycelial plugs of 5 mm diameter of *F. Culmorum* and *Trichoderma* strains (7-day-old) were placed on opposite sides of PDA Petri plate (8.5 cm diameter, containing 20 mL of PDA medium) at 5 cm from each other and at equal distance from the periphery. Co-cultures were incubated at 28 °C for six days. The radial growth of the *F. culmorum* in the presence of *Trichoderma* strains was measured, and the inhibition rate (IR) was calculated as follows: IR = R1 − R2/R1 ∗ 100, where R1 is the colony radius of *F. culmorum* in the control plate, and R2 is the colony radius of *F. culmorum* in the presence of *Trichoderma* strain. Each biological condition was repeated five times.

#### 2.4.3. Determination of Diffusible and Volatile Metabolite Productions

The activity of *Trichoderma* diffusible compounds was tested using cellophane method described by Dennis and Webster [47]. Strains were grown on Petri dishes containing a sterile cellophane sheet over the PDA medium. After five days of incubation at 28 °C, cellophane was removed, and a 5 mm plug of *F. culmorum* was placed in the center of each Petri plate. *F. culmorum* grown on untreated PDA served as control. Assays were carried out 5 times, and plates were incubated at 28 °C for five days. Results were expressed as the inhibition rate (IR) calculated according to the formula mentioned above, where R1 is the colony radius of *F. culmorum* in the control plates, and R2 is the colony radius of *F. culmorum* in the treated plates. To verify the volatile metabolite production, a qualitative method was used. Before inoculation, a 1.5 cm wide agar strip was removed from the mid-portion of PDA medium. A 5 mm diameter disk of *Trichoderma* strain was placed on one side, while a disk of *F. culmorum* was placed on the opposite side. PDA plates inoculated only with *F. culmorum* served as controls. All plates were sealed with parafilm, and this assay includes 5 replicates per biological condition. After seven days of incubation at 28 °C, the RI was determined according to the abovementioned formula.

#### 2.4.4. Qualitative Determination of Detoxifying and Lytic Enzyme Production

Qualitative determination of extracellular enzymes was performed on 7-day-old *Trichoderma* cultures. All assays were carried out in triplicate, and a non-inoculated plate was used for each biochemical test as negative control.

*Catalase.* Catalase activity was assessed with 3% *v/v* hydrogen peroxide. A 5 mm diameter plug of actively growing *Trichoderma* culture was put on a glass slide in a Petri plate, on which a drop of hydrogen peroxide was applied. The appearance of immediate bubbling reveals the positive catalase ability of the strain.

*Protease.* For protease enzyme detection, *Trichoderma* strains were grown on Glucose–Yeast–Peptone medium (GYP) with the following composition (per liter of distilled water): glucose, 1 g; yeast extract, 0.1 g; peptone, 0.5 g; agar, 15 g, supplemented with 1% Skim milk (pH 6.5) [48]. After seven days of incubation at 28 °C, a positive result for protease production was revealed by the appearance of a clear zone around the *Trichoderma* colony.

*Amylase.* To evaluate the amylase activity, *Trichoderma* strains were inoculated following the previous conditions on GYP medium enriched with 1% soluble starch as substrate for amylase enzyme [49]. After seven days of incubation, plates were flooded with an aqueous solution of Iodine 1% (*w*/*v*) in potassium iodide 2% (*w*/*v*) for 10 min, and then, the iodine solution was carefully removed by decanting each plate. Amylase activity was revealed by the presence of a clear halo around *Trichoderma* colony.

*Chitinase.* Chitinase assay was performed by growing *Trichoderma* strains on a basal medium comprising (per liter of distilled water): colloidal chitin, 1.5 g; K_2_HPO_4_, 2.7 g; MgSO_4_·7H_2_O, 0.7 g; NaCl, 0.5 g; KCL, 0.5 g; yeast extract, 0.13 g, agar 15 g, and pH 5.5 [50]. After seven days of incubation, according to the previously cited conditions, plates showing a transparent halo around the colony were considered as Chitinase positive. The method of Roberts and Selitrennikoff [51] was used for colloidal chitin preparation. Five grams of chitin powder was added to 60 mL concentrated HCl and kept at 4 °C overnight with vigorous stirring. The mix was added to 500 mL of ice-cold 95% ethanol with rapid stirring and left overnight at room temperature. The precipitate was collected by centrifugation at 5000× *g* for 20 min at 4 °C, washed with sterile distilled water several times until the colloidal chitin became neutral (pH 7.0) and stored in the dark at 4 °C before being used.

*Endoglucanase.* Czapek-agar medium was used to reveal cellulose degradation [52]. It is composed of (per liter of distilled water) NaNO_3_, 2 g; KH_2_PO_4_, 1 g; KCl, 0.5 g; MgSO_4_·7H_2_O, 0.5 g; FeSO_4_·7H_2_O, 0.01 g; agar, 20 g, and supplemented with 0.1% carboxymethylcellulose (CMC) as the sole carbon source (pH 4.5 adjusted with 100% glacial acetic acid). Petri dishes were inoculated with plugs of the different *Trichoderma* strains and incubated at the same cited conditions. After seven days, plates were flooded with an aqueous solution of Congo red (0.1% *w*/*v*). After five minutes of reaction, this solution was gently removed, and dishes were washed with 5 M NaCl to reveal the degradation halo.

### 2.5. Bioassays for Plant Growth Promoting Traits

#### 2.5.1. Solubilization of Inorganic Phosphate

The capacity to solubilize phosphate was tested on solid Pikosvskaya medium (PVK) [53] that had the following composition (per liter): 10 g of glucose, 0.5 g of yeast extract, 0.5 g of (NH_4_)_2_SO_4_, 0.1 g of MgSO_4_, 0.2 g of KCL, 15 g of agar and 5 g of Ca_3_(PO_4_)_2_ as sole source of phosphate. Plates were inoculated with a plug of 5 mm of *Trichoderma* (7 days old) with 3 repetitions for each strain per plate. After incubation for 7 days at 28 °C, the presence of a clear halo around the colony indicates the presence of phosphate dissolving activity. The experiment was conducted twice, with three sets of replication plates.

#### 2.5.2. Colorimetric Detection of Indole-Related Compounds

Indole-related compound production was determined using a colorimetric method [54]. Two plugs of 5 mm of each *Trichoderma* strain (7 days old) were transferred in 10 mL of Potato Dextrose Broth (PDB) supplemented with 0.1% L-Tryptophan and then incubated under shaking at 28 °C for 72 h. Supernatant was collected from 1 mL of culture after 5 min of centrifugation at 14,000 rpm, then 100 µL of this supernatant was added to 200 µL of Salkowski reagent in triplicate. After incubation at room temperature for 30 min, the optical density was recorded at 530 nm [55]. The amount of indole-related compounds was calculated from a standard curve generated by suspending IAA in 100% ethanol at a concentration of 1 mg/mL and then diluted in PDB medium to a concentration of 1, 2, 3, 4, 5, 7, 8, 9 and 10 µg/mL. PDB with or without L-Tryptophan served as controls. The experiment was conducted with three biological replicates.

#### 2.5.3. Hydrogen Cyanide (HCN) Production

*Trichoderma* spp. was grown on solidified PDA supplemented with 4.4 g/L of Glycine or Succinate. White filter paper discs cut into the same size as the upper lid of the Petri dish were immersed in an alkaline Picric acid solution (Picric acid, 0.5% (*v*/*v*), Sodium carbonate, 1.25% (*w*/*v*); in water), and carefully positioned on the lid of each plate. Plates were sealed with Parafilm and incubated for seven days at 28 ± 2 °C. After incubation, HCN production was observed by the color changes of the filter paper from yellow to light brown or reddish brown, which indicated the production of HCN [56]. Because HCN can be produced at very low concentrations, the filter paper was then immersed in 5 mL of distilled water to dissolve the precipitate, and the optimum density was measured at 625 nm using a spectrophotometer [57]. Each measurement corresponds to the mean of five biological replications.

#### 2.5.4. Ammonia Production

Ammonia production was determined using a colorimetric method [58]. Each *Trichoderma* strain was tested for the production of ammonia in peptone water. Two plugs of 5 mm of actively growing *Trichoderma* (7 days old) were added to 10 mL peptone water and then incubated under shaking at 28 °C for 7 days. Cultures were centrifuged at 3500 rpm for 10 min. A volume of 50 µL of Nessler’s reagent (7% KI; 10% HgCI_2_; 50% aqueous solution of NaOH (32%)) was added to 1 mL of every culture supernatant. Development of yellow to brown color indicates the presence of ammonia. The optical density was measured at 450 nm, and ammonia production was calculated using a standard curve of ammonium sulfate ((NH_4_)_2_SO_4_) solution at different concentrations of 0.1, 0.5, 1, 1.5, 2, 2.5, 3, 3.5 and 4 µmol/mL. Each measurement corresponds to the mean of three biological replications.

### 2.6. Bioassay of Trichoderma Strains for Plant Growth Promoting and Fusarium Seedling Blight Disease Suppression

The plant growth-promoting effect, as well as the suppression of *Fusarium* seedling blight (FSB) disease of 6 isolated *Trichoderma* strains on durum wheat, was tested by germinating the seeds in axenic conditions and then by monitoring their growth under greenhouse conditions for 3 weeks. The variety “Khiar”, which is one of the most used and productive durum wheat varieties in Tunisia, has been chosen for this study [59]. Seeds were provided by the National Institute of Agronomic Research of Tunisia (INRAT).

#### 2.6.1. Effect on Seed Germination in Axenic Conditions

Seeds were surface sterilized as described by Fernandez and Chen (2005) [60] with minor modifications. Seeds were soaked in an aqueous solution of 0.5% sodium hypochlorite (NaClO) for 5 min, washed three times with sterile distilled water, dried overnight on filter paper under aseptic conditions and placed in sterile dishes for further use. For fungal preparation, *Trichoderma* strains were grown on PDA medium under 12 h regime of fluorescent light at 26 ± 2 °C for 7 days to promote sporulation. Fungal spore suspension of *F. culmorum* was prepared from a 2-week-old culture. The collected fungal suspension was filtered, macrospores were counted, and the concentration was adjusted to 10^7^ spores/mL in sterile PDB.

Seeds were soaked for 24 h under slow shaking in 20 mL of *Trichoderma*-PDB suspensions (10^7^ spores/mL) to promote *Trichoderma* growth on seeds (50 seeds in a falcon tube of 50 mL for each repetition). Seeds soaked only in PDB served as control, and each treatment was repeated 4 times. Seeds were dried on filter paper under aseptic conditions and then sown in square Petri dishes of 12 ∗ 12 cm (50 seeds per plate) containing 1% of water agar medium (10 g/L) previously autoclaved and sterilized for 30 min under UV radiations [61]. For Fusarium seedling blight suppression, seeds were prepared in a similar way, coated with *Trichoderma* strains and then sprayed with 1 mL of *F. culmorum* spore suspension adjusted at 10^7^ spores/mL. Seeds sprayed only with *F. culmorum* served as positive control. All the following treatments, i.e., for non-treated seeds, seeds treated only with *Trichoderma*, seeds treated with *Trichoderma* and *F. culmorum* and seeds treated only with *F. culmorum*, were repeated 4 times. All plates were sealed with parafilm and incubated in the dark at 25 °C for 5 days, and some parameters, which include the final germination percentage (FGP), fresh weight of seedlings and root and shoot lengths, were measured. FGP was calculated using the following formula: FGP = germinated seeds/total seeds ∗ 100.

#### 2.6.2. Effect on Seedling Growth under Greenhouse Conditions

After 7 days of germination in axenic conditions, PDB control and fungal-treated seedlings (treatments described in the Section 2.5.1) were carefully removed from water agar medium and transferred into pots of 6 ∗ 6 ∗ 6 cm, with 1 seedling per pot. Each experiment incorporates six biological repetitions. Substrate consisted of 1/3 sterilized sand and 2/3 potting soil. All pots were placed in greenhouse under the following conditions: 16/8 h light/dark photoperiod under an illumination of 150 µmol/m^2^/s photosynthetic photon flux density, a temperature of 23 ± 0.5 °C, and a relative humidity of 65 ± 5%. Pots were watered every two days with tap water for 21 days until all plants were harvested to complete all the ecophysiological measurements (fresh and dry weights, shoot and root lengths, *cf* Section 2.5.3) and molecular analyses (*cf* Section 2.7).

#### 2.6.3. Measurements of Total Chlorophyll, Epidermal Flavonols, Nitrogen Balance Index (NBI)

Chlorophyll content (Chl), epidermal flavonols (Flav) and NBI were measured in vivo using Dualex sensor (Force-A, France), which is a clamp that performs instantaneous and non-destructive quantifications [62]. All proxy measurements were done on uniform, fully developed and light-exposed leaves of the same plant 21 days after fungal treatment.

#### 2.6.4. Total RNA Isolation, cDNA Synthesis, and Quantification of the Defense-Encoding Gene Accumulation

From the experimental set-up used for the ecophysiological analyses and concomitantly to the execution of the ecophysiological measurements, the aerial and root tissues were collected from fifteen randomly selected plants, which were then distributed into 3 biological replicates, each comprising the tissues of 5 plants. All samples were immediately frozen in liquid nitrogen and stored at −80 °C before being used. Samples were ground to a fine powder with 3 metallic balls for 90 s (Ø 5 mm) in Eppendorf tubes. Total RNA was extracted using a CTAB extraction buffer (Cetyltrimethylammonium Bromide), including the elimination of the genomic DNA as previously described in Ben Amira et al. (2018) [63]. The quality and quantity of the recovered total RNA were estimated by a NanoDrop ND-1000 spectrophotometer. Two μg of total RNA were reverse-transcribed with Oligo-dT using the SuperScript^®^ III First-Strand Synthesis System for RT-PCR (Invitrogen, Carlsbad, CA, USA) according to the manufacturer’s instructions. The quantification of the transcript levels of genes was measured by real-time quantitative PCR from 1 µL of cDNA diluted 30-fold with sterile water. Expression analysis of the targeted genes involved in the defense responses (listed in Table S1 in Supplementary Materials) was performed with an Applied Biosystems StepOnePlusTM Real-Time PCR System (Applied Biosystems, Foster City, CA, USA) using TakyonTM Rox SYBR^®^ MasteMix dTTP Blue (Eurogentec, Liege Science Park, Belgium), according to manufacturer’s recommendations. PCR cycling conditions were 1 cycle for 3 min at 95 °C, followed by 40 cycles for 10 s at 95 °C, 15 s at 60 °C, and 10 s at 72 °C. PCR reactions were ended by generating a dissociation curve of 60–95 °C with an increment of 0.3 °C/15 s to ensure primer dimers and nonspecific amplifications. Three reference genes (*Actin* (*ACT*), *Heterogeneous nuclear ribonucleoprotein* (*RPN*), and *RNaseL inhibitor-like* (*RLI*)) were used to normalize the expression results of the target genes. The reference genes were chosen from a panel of widely used reference genes and specifically selected for their stable expressions between samples and treatments. Moreover, each referrer belongs to protein families involved in different cellular processes in order to minimize the risk of co-regulations. The expression level of the normalized target genes in each RNA preparation was calculated using the 2^−∆∆Ct^ [64]. All reactions were performed in triplicate. Primer pairs are detailed in Table S1 in Supplementary Materials. PCR efficiencies (*E*) and conditions were determined by comparing threshold values in a dilution series of the RT product (×10, ×20, ×40, ×80), followed by non-template control for each primer pair. PCR efficiencies are given in Table S1 in Supplementary Materials.

#### 2.6.5. PCR Detection of Trichoderma and *F. culmorum* Strains in Wheat Roots and Leaves

The presence of *Trichoderma* or *F. culmorum* isolates was qualitatively characterized in the cDNA samples used for gene expression defense analysis. Amplifications targeted the *tubulin*-encoding gene with primer pairs specific to each fungal genus (*Trichoderma*: 5′-GTACTAAGTTGTTTCTTTGCTGTTG/5′-CTCTTGTACATACACCAATTGCTC; *Fusarium*: 5′-GAGTACTAAGCGGTTTCGGATGC/5′-GCATGCCGCGTGCGCGCAAGGTTC; (5′-Forward/5′-Reverse)) [63]. The PCRs were carried out on cDNAs diluted by 1:5 and 1:20, according to the PCR cycling conditions described above, and with a hybridization temperature fixed at 68 °C.

### 2.7. PCR Statistical Analysis

Ecophysiological response variables were collected from at least three biological replicates. The standard one-way Analysis of Variance (ANOVA) was carried out to test the effect of Strain factor on each of these variables. Homogeneity of variance between strains was examined using Levene’s test [65] using the car::leveneTest function [66], and normality was examined through visual inspection of the residuals as well as the Shapiro–Wilk test [67] using the stats:shapiro.test function using R Statistical Software (v4.2.2; R) [68]. If the homogeneity of variance assumption was violated, Welch One-Way ANOVA [69] was carried out using the stats:oneway.test function. Multiple comparisons of means were carried out using Tukey’s HSD post hoc test, with the “rstatix::tukey_hsd()” for standard ANOVA or the Games-Howell test [70] with the “rstatix::games_howell_test()” for Welch ANOVA [71]. Omega squared was used as a measure of the effect size for standard ANOVA and as estimation for Welch ANOVA [72]. It is an estimate of how much variance in the response variables is explained by the Strain factor. For omega squared, 0.01 to <0.06 indicates a small effect size, 0.06 to <0.14 is a medium effect size, and ≥0.14 is considered a large effect size [73]. It was computed with MOTE::omega. F function [74]. The Kruskal–Wallis test [75] based on ranks of data was computed with stats:kruskal.test function and used instead of a one-way ANOVA in case the normality assumption did not hold. The rank epsilon squared was then computed with rcompanion: epsilonSquared and utilized as a measure of the Kruskal–Wallis test effect size [76]. It varied between 0 and 1 and indicated the ratio of variance in the response variable explained by the explanatory variable, the Strain factor. It also indicated the degree to which one strain had data with higher ranks than the other strain. For rank epsilon-squared, 0.01 to <0.08 indicates a small effect size, 0.08 to <0.26 is a medium effect size and ≥0.26 is considered a large effect size [77]. Kruskal–Wallis test was followed by Dunn’s *post hoc* test [78] using agricolae::kruskal [79] for multiple comparisons of medians. All statistical analyses were performed using R Statistical Software (v4.2.2; R) [68].

Concerning the molecular analyses, statistical analyses were performed with R (v4.2.3; R) [68]. Gene expressions obtained with DCT data were normalized using BestNormalize (1.8.3) package. Normality was tested with Shapiro–Wilk’s W test [80]. Homoskedascity was tested with Bartlett’s test [81]. Significant differences were called using Student’s *t*-test implemented with ggpubr (version 0.6.0) [82] and tidyverse (version 2.0.0) [83] packages. Differential gene expressions were selected after false discovery rate control using Benjamini–Hochberg procedure [84] and filtered at 5% error level.

## 3. Results and Discussion

### 3.1. Phylogenetic Position of the Trichoderma Isolates

**Figure 1:**
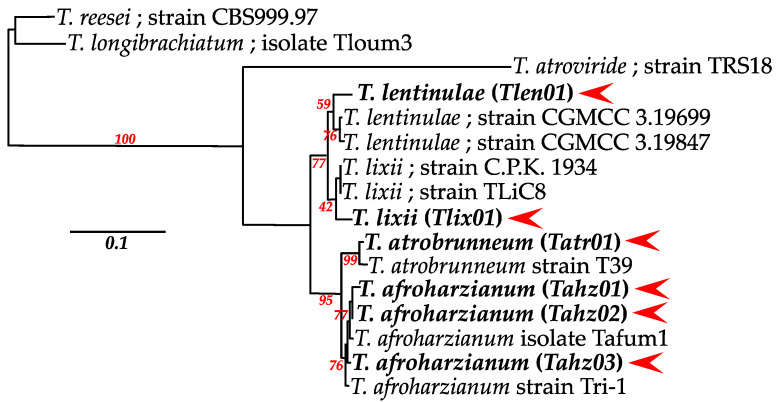
ITS-*tef1*-*rpb2* based molecular phylogenetic analysis of the six *Trichoderma* spp. isolates (red triangle) with closed sequences retrieved from NCBI. The analysis was generated using the maximum likelihood analysis implemented in the PhyML program. Values of the bootstrap analysis (1000 repetitions) are given at the nodes. Bar 0.1 corresponds to the nucleotide substitution per sequence position. Each new strain is labeled with an acronym (e.g., *Tahz01*, *Tahz02*, etc.) that will be used throughout the article.

A total of 111 strains were isolated on TSM medium from rhizospheric soil samples. In order to identify the potential strains from the diversity of colonies, a fragment of approximately 575 bp of the *ITS* region was obtained from PCR amplification using ITS1 and ITS4 primers, followed by forward and reverse sequencing. The BLASTn alignment on the NCBI database enabled us to retrieve six different sequences that share about 98% to 100% similarity with *Trichoderma* strains. These sequences were divided into three large groups with highly significant bootstrap values (NJ (Neighbor-Joining) method) (Figure 1): a first group of 66.66% from the total isolates represented by three different *Trichoderma harzianum* strains, a second group of 14.41% represented by one *Trichoderma lixii* and a third group of 18.91% represented by two *Trichoderma* sp. close to the *T. harzianum* complex. Every sequence was recorded in NCBI, and the related GenBank accession numbers are given in Figure 1.

Interestingly, all six isolates were placed in the *T. harzianum* complex [36,37,38]. Since ITS loci analyses are insufficient to distinguish the species’ delimitation within a closely related *Trichoderma* species complex such as *T. Harzianum* [85,86,87], partial regions of loci encoding *tef1* and *rpb2* were sequenced from these six “*T. harzianum*” isolates. These loci are powerful sources of information due to their inherent interspecific variations and are extensively used to accurately resolve the taxonomy of *Trichoderma* [88,89]. The analysis of concatenated ITS-*tef1*-*rpb2* sequences confirmed that the six isolates belong to a common complex. Moreover, it facilitated the identification of the two *Trichoderma* sp. isolates, as being the *T. afroharzianum* (*Tahz03*) and *T. lentinulae* (*Tlen01*) species. Similarly, the species’ range was substantially clarified for the three *T. harzianum*, which are precisely affiliated with two *T. afroharzianum* (*Tahz01* and *Tahz02*) and one *T. atrobrunneum* (*Tatr01*). *T. atrobrunneum* is a new species clade closely related to the *T. Afroharzianum* taxon but clearly distinguished within the *T. harzianum* sub-complex [90]. Finally, this multi-loci analysis confirmed the correct identification of *T. lixii* (*Tlix01*), which is phylogenetically close to *T. lentinulae* and forms a highly supported clade with it. These two clades, *T. afroharzianum*/*T. atrobrunneum* and *T. lixii*/*T. lentinulae*, have been separated from the *T. harzianum* complex [38,91].

Each strain exhibited the expected morphological characteristics (macro- and microscopic) of the *Trichoderma* genus (Figure S2 in Supplementary Materials). On the Potato Dextrose Agar (PDA) medium, *Trichoderma* strains appeared as fast-growing colonies, concentric halos and floccose or compact surfaces that looked similar to tufts. The mycelium started out white, then turned green due to the production of conidia. Under an optical microscope, abundant sporulation of conidia with smooth or rough appearance was observed, conidiophores were branched and irregularly verticillated, and phialides were generally ampliform or fusiform and arranged in clusters, according to Gam et al. [92].

All of these species have also been reported all across the world; however, to our knowledge, this is the first report of *T. atrobrunneum*, *T. lixii* and *T. lentinulae* in an agroecological context in Tunisia, and they have never been introduced as beneficial agents in wheat.

Some *Trichoderma* strains were isolated from Tunisian soil and plant habitats. Their characterization was based on evaluating their occurrence and diversity within contrasting bioclimatic zones [93,94] or on their PGP and/or BCA abilities on perennial (*T. harzianum* on Olive trees [32]) or annual crops (e.g., *T. harzianum* and *T. viride* on Potatoes [95] and Faba bean [96]; and *Trichoderma* spp. on wheat [33,34]). The strains mentioned in these studies represented different ranges of *Trichoderma* complexes, and these strains and some of our isolates presented common strain complexes, of which *T. (afro)harzianum* appeared to be the most representative. However, little is known about the diversity of *Trichoderma* spp. that occurs or interacts with wheat, with most studies focusing on *T. harzianum* and more marginally on *T. gamsii*, *T. viride*, *T. koningii* or sp. [16,33,35,97,98]. *T. harzianum* T22, originally derived from the fusion of two auxotrophic strains, T-95 and T-12, and the first fungus registered by the Environmental Protection Agency (EPA) for biocontrol of plant diseases in 1989 [99], is still one of the most widely used biopesticides today [16,100,101]. The genus *Trichoderma* has more than 150,000 members, and of the hundreds of *Trichoderma* sp. species recorded, only a fraction is exploited as biocontrol agents on field crops. The diversity of the above-mentioned Tunisian strains added to our own (with the originality of the *T. atrobrunneum*, *T. lixii* and *T. lentinulae*) and remained, nevertheless, a very promising source of beneficial agents. To this end, the study of this fungal genus is still a very active research area, especially as the beneficial influences of these fungi are dependent on the fungal strains and the plant species with which they interact. Likewise, this situation is paradoxically prevalent in durum wheat, especially during FSB on durum wheat caused by *F. culmorum*. Because the plant growth-promoting potential varies between different *Trichoderma* species and strains, and also because local isolates are now favored over *Trichoderma*’s subsequent use as biofertilizer and biocontrol agents, we have evaluated the plant growth-promoting abilities and the antagonism activities of these six native *Trichoderma* strains isolated from wheat rhizosphere against that of *F. culmorum* in vitro and in vivo.

### 3.2. Trichoderma Isolates Exhibit Differential PGP Abilities

**Figure 2:**
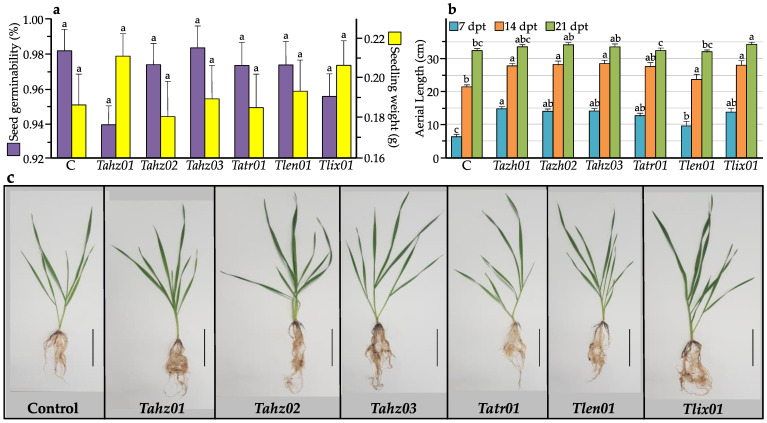
Effect of *Trichoderma* strains on seed germination (seed germinability and seedling weight) at 7 days after fungal treatment and on wheat plant morphology (**a**) and leaf length (**b**) over time (i.e., 7, 14 and 21 days after treatment). (**c**) Morphological status of the plants at the end of the experiment (21 days after treatment). C, PDB control. Scale bars in photographs represent 10 cm. Data correspond to the mean of fifty (**a**) and twenty (**b**) plants, respectively. Bars represent the standard biological error. Distinct letters in the same column indicate significant differences (*p* ≤ 0.05).

During germination in axenic condition, coating seeds with all six *Trichoderma* strains showed no significant effect on the final germination percentage effect (*p*-value for Kruskal–Wallis rank sum test = 0.4971; rank epsilon2 = 0.199) or on total fresh weight of 7-day-old seedlings (*p*-value for standard ANOVA = 0.246; omega squared = 0.029) (Figure 2a).

In addition, no particular symptom related to any pathogenicity expression of our tested *Trichoderma* strains was observed on the variety of wheat Khiar used in this study, whether during germination or seedling growth experiments (Figure 2c; Figures S3 and S4 in Supplementary Materials). These first symptomatological inspections are of the utmost significance and should be performed on all wheat genotypes commonly grown in Tunisia. Indeed, some studies have shown that the treatment with *Trichoderma* either had no effect or was even detrimental. In this respect, several *Trichoderma* species are included among pathogens of cultivated plants: e.g., *T. viride* is the causal agent of onion green mold rot [102], and *T. afroharzianum* is responsible for the occurrence of a new ear rot disease in maize in Europe [103].

**Figure 3:**
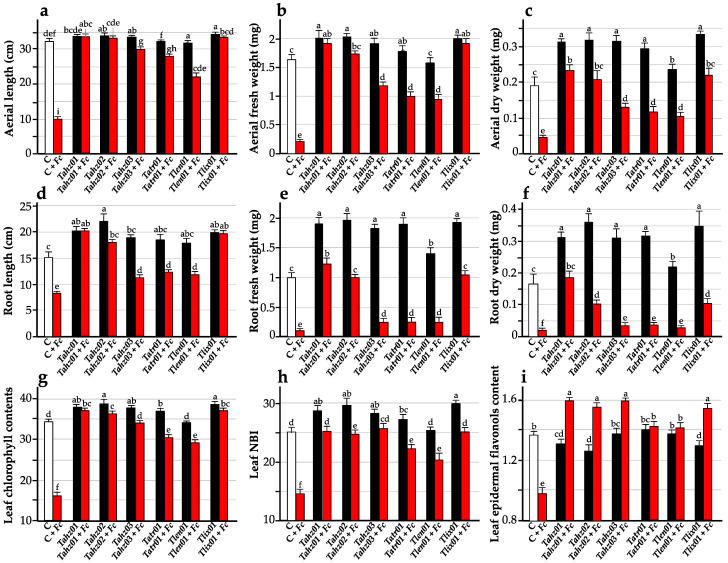
Effect of *Trichoderma* strains on physio-morphological parameters: (**a**) aerial length, (**b**) shoot fresh weight, (**c**) shoot dry weight, (**d**) root length, (**e**) root fresh weight, (**f**) root dry weight, (**g**) leaf chlorophyll content, (**h**) Leaf NBI, and (**i**) Leaf epidermal flavonols content of wheat plants (*Khiar* variety) after 21 days of cultivation in greenhouse conditions. White bars: control condition (C), which corresponds to healthy plants from seeds treated with sterile PDB (C); Black bars: plants from seeds inoculated with *Trichoderma* isolates; Red bars: plants inoculated with *F. culmorum* (*Fc*) or co-inoculated with *F. culmorum* and *Trichoderma* isolates. Data correspond to the mean of twenty plants. Bars represent the biological standard error. Distinct letters in the same column indicate significant differences (*p* ≤ 0.05).

Seven days after planting seedlings in pots, the plant growth-promoting ability was clear, and every isolate exhibited a significant (*p*-value for Welch’s ANOVA = 1.53 × 10^−7^; estimated omega squared = 0.562) biostimulatory effect on shoot height (Figure 2b,c). This impact was still significant 14 days later (*p*-value for Welch’s ANOVA = 1.80 × 10^−9^; estimated omega squared = 0.693) (except for *Tlen01*) and even up to 21 days later (*p*-value for standard ANOVA = 1.75 × 10^−5^; omega squared = 0.193) (except for *Tlen01* and *Tatr01*). The effect size for the shoot height after 7 days, as well as after 14 days and 21 days, was large; the observed differences among strains for the shoot height were of practical significance. At day 21 in the greenhouse, the lengths of shoots from seeds coated with *Trichoderma* strains were 4% to 11% longer, depending on the isolate, than those of untreated seeds. The best stimulatory effects on seedling growth were observed with the isolates *Tahz02* and *Tlix01* compared to the other tested strains. These effects were manifested by a highly significant (*p*-value for Kruskal–Wallis rank sum test < 0.0001) increase in both shoot (rank epsilon2 = 0.644) and root (rank epsilon2 = 0.535) length as well as fresh (shoot: rank epsilon2 = 0.591; root: rank epsilon2 = 0.621) and dry biomass (shoot: rank epsilon2 = 0.602; root: rank epsilon2 = 0.661) (Figure 3a–f). For all these variables, rank epsilon2 values showed a large effect size between strains; the magnitude of the differences in the mean scores among the strains seems to be large on these variables.

Additional ecophysiological traits correlated with the nitrogen status, such as leaf chlorophyll content and nitrogen balance index (NBI), as well as the secondary compounds related to plant defenses with the epidermal flavonols, were measured using Dualex, a non-destructive method commonly used for assessing ecophysiological traits in many plant species, including wheat [104]. Chlorophylls are key compounds for the photosynthetic process, and a great deal of research revealed the direct relationship between the number of chlorophylls and photosynthetic rates. As for the NBI, it is defined as the ratio of chlorophylls to epidermal flavonols (Chl/Flav) [104]. This proxy is an informative indicator for crop growth as the high and low nitrogen status can be quickly monitored by measuring NBI, providing accurate information for farmers to make timely N management decisions. It is generally assumed that a high NBI is an indicator of both high nitrogen content and high nitrogen use efficiency [105]. Results showed a remarkable impact on total chlorophyll content when seed coating with *Trichoderma* strains (*p*-value for Kruskal–Wallis rank sum test < 0.0001; rank epsilon2 = 0.411) (Figure 3g). All tested *Trichoderma* strains (except for *Tlen01*) significantly enhanced leaf pigments as demonstrated by the measurement of chlorophyll content: *Trichoderma*-treated plants exhibited 107% to 112% higher chlorophyll content which was positively correlated with the nitrogen content [106]. The improvement in the photosynthetic capacity of plants, modulated by various endophytic *Trichoderma* spp., would be attributed to an increase in the number of photosynthetic pigments (in addition to the expression of genes regulating the biosynthesis of chlorophyll and proteins integrated into the light-harvesting complex or the Calvin cycle [107]). These cell responses would be elicited by specific secondary metabolites and/or VOCs emitted by the fungus [108,109]. All isolates secreted VOCs (phenomena described in Section 3.4.2), and it is plausible that these emissions are involved in the observed increase in photosynthetic pigment content and in the ensuing promotion of plant growth.

Similarly, NBI, as well as leaf flavonol content, were significantly influenced by the strains (*p*-value for Kruskal–Wallis rank sum test < 0.0001), and large effect sizes were detected (rank epsilon2 = 0.333 for NBI, and 0.369 for flavonol). NBI has significantly increased between 108% to 118% under the effect of all *Trichoderma* strains except for the strain *Tlen01*, which has almost the same NBI as the control plants (Figure 3h). In contrast, leaf flavonol content decreased significantly for *Tahz02* (−8%) and *Tlix01* (−5%) and showed a similar trend for *Tahz01* (−28%). *Tatr01* and *Tahz03 and Tlen01* showed the same flavonol content as the control plants (Figure 3i). Flavonols (or flavonoids) are abundant and ubiquitous secondary compounds in plants. They are produced through the phenylpropanoid pathway, and their abundances can be regulated through de novo biosynthesis or rapid translocation and modification of existing compounds. Because of their remarkably versatile function, these molecules integrate many physiological processes, including plant growth (as well as disease resistance, which will be discussed in Section 3.4.1.). In this respect, their accumulation is a rapid and long-lasting physiological response to PGP in general and *Trichoderma* spp. in particular [110,111,112]. In our study, the slight decrease in (only for *Tahz01*, *Tahz02* and *Tlix01*) or stable abundances of these flavonols for the other strains are not contradictory. Indeed, the chlorophyll content is positively correlated with the nitrogen content, while the content of epidermal flavonols is inversely correlated to nitrogen content [106]. Thus, the decreases in flavonoid accumulations observed with the strains exhibiting the strongest PGP potentials would reflect basal constitutive amounts of phenolic compounds in wheat leaves that can remain under the influences of external stimuli, a priori of root origin and induced under *Trichoderma* activity. In other words, these differential contents between strains would result from molecular and systemic signals, which would be strain dependent. In addition, our study is based on the duration of the interaction and not on short time intervals after the protagonists’ interactions. In this respect, the abundances recorded 21 days after germination do not prejudge those that could have been recorded after a few hours or a few days of treatment. For example, a low level of flavonols correlated with a high NBI was also observed in maize seedlings from seeds inoculated with *T. atroviride* [106]. Similarly, we hypothesize here that the low flavonol abundances recorded for some strains echo the plant growth promotion induced by these same strains, knowing that under conditions of no stress, these two physiological processes interfere with each other. In any case, the content of epidermal flavonols recorded in wheat plants treated with the six Trichoderma isolates confirms that treatment with these strains did not affect the general health of the plants.

### 3.3. Trichoderma Isolates Exhibit Differential Biochemical Potentials

The isolated *Trichoderma* strains in this study developed significant and long-lasting PGP abilities four weeks after seed coating, and these abilities are strain-dependent. This finding strongly suggested that these strains produced growth-regulating factors or displayed metabolic activities that are involved in the increase of seedling growth kinetics. In this respect, the PGP potential of the six *Trichoderma* strains was assessed in vitro through the production of ammonia (NH_3_), Hydrogen cyanide (HCN) and indole-related compounds and their ability to solubilize phosphate (Ca_3_(PO_4_)_2_) (Table 2).

In our study, only the ammonia and the indole-related derivatives tests were positive. All strains were able to produce ammonia at the same level, and a level in the range of 14.1 µg/mL to 16.7 µg/mL. *Tahz01* was found to produce the highest amount of ammonia. This result is supported by several studies showing the ability of *Trichoderma* to produce ammonia [61,113,114]. Ammonia is an essential trait linked to plant growth promotion, and the produced ammonia has been shown to supply nitrogen to the plant, thereby promoting root and shoot elongation and biomass [115]. Likewise, all strains produced indole-related derivatives, with the exception of *T. lixii* (*Tlix01*), for which no production was detected for the three technical repetitions. *T. afroharzianum* strains seem to be the most productive, in particular *Tahz02* with 12.4 µg·mL^−1^ and, to a lesser extent, *Tahz01* with 4.2 µg·mL^−1^. The other isolates showed lower production ranges (i.e., 0.93 to 1.7 µg·mL^−1^). These levels were commonly recorded for some *Trichoderma* spp. [116,117,118] or rhizospheric PGPs [119,120]. Several factors can be put forward to explain these variations in the production of indole-like derivatives between strains: for example, the differential modulation of the biosynthetic pathways and the related-regulatory pathways or the thermodynamic specificities of enzymes that convert primary heterocyclic aromatic organic compounds (Tryptophan) into several indoles and related conjugates forms [121]. A colorimetric method using Salkowski’s reagent was performed to quantify the production of indole-related compounds, but it does not allow the specific determination of Indole-3-acetic acid (IAA), even if the standard curves were carried out with pure IAA. Indeed, the levels of IAA produced in plant biological tissues or by microorganisms are infinitesimal to the order of nM [122]. However, *Trichoderma* spp. increased systemic biomass production and lateral root growth promotion in several plant species such as *Arabidopsis* [123], tomato [124] or cucumber [125], two physiological processes which are known to be dependent on IAA. We do not exclude that IAA is produced by our isolates, but this hypothesis must be validated by additional and more precise analytical methods such as High-Pressure Liquid Chromatography (HPLC) [126], Liquid Chromatography Electrospray Ionization Tandem Mass Spectrometric (LC-ESI-MS/MS) [127] or again by High-Performance Thin Layer Chromatography HPTLC [128]. It is nevertheless true that Salkowski’s reagent test is a useful primary index in the screening of PGP functional rhizospheric microorganisms correlated to the production of various indole-related compounds and derivatives—among which IAA is a key substance—such as *Trichoderma* genus.

It is suggested that over 80% of rhizosphere bacteria are capable of synthesizing IAA and/or indole-related derivatives. Correlated to these specific metabolic syntheses, these strains are identified as biostimulators of plant growth, particularly by stimulating lateral and adventitious root length [129]. An increase in root surface area allows plants to access more minerals and nutrients from the soil and, presumably, to absorb them better [130]. It is confusing but nevertheless very interesting that *T. lixii* is one of the best-performing PGP isolates while it does not seem to produce indole-like derivatives. Although *T. lixii* is usually presented as the sexual state of *T. harzianum* in nature, we demonstrate here that each strain is genetically distinctive, including in their ability to produce Indole-like metabolites. To the best of our knowledge, no bibliographic reference reports the lack of production of indoles-like and/or IAA-related derivatives by *T. lixii* spp. Further analytical research (e.g., HPLC, LC, or HPTLC) would provide a definitive answer to this observation.

A contrario, all strains did not appear to metabolize phosphate nor produce HCN. Phosphate is essential for long-standing crop production, and although it is present in large amounts in soils, it needs to be mainly solubilized by the soil microbial community in order to be mobilized by plants [131] (Fankem et al., 2006). As for HCN, its production is intricately related to antifungal activity and the priming of the root length and root hair germination [132]. Each test was performed qualitatively on Pikovskaya’s agar medium, which was supplemented with tri-calcium phosphate as an insoluble phosphate source, and by using Glycine or Succinate as primary substrates, respectively. The absence of these activities has been found in other studies applying the same methods [61,113,118], although some positive activities have also been demonstrated [133,134].

### 3.4. Trichoderma Isolates Exhibit Functional BCA Abilities against F. culmorum

*Trichoderma* spp. uses several antagonistic strategies against plant diseases. This includes the expression of a direct aggressiveness against its prey (i.e., the phytopathogenic fungi) by using mycoparasitism, antibiosis and secretion of extracellular cell wall-degrading enzymes and by eliciting transcriptional expression of a large set of host resistance-related genes.

#### 3.4.1. Trichoderma Isolates Annihilate the Expression of Fusarium Seedling Blight Disease (FSB) Symptoms in Wheat

In order to confirm the bioprotective capacities of our isolates against Fusarium seedling blight (FSB) induced by *F. culmorum* in wheat, two bioassays were conducted, including a germination test in a water agar medium (in vitro), followed by a greenhouse seedling growth experiment for up to 3 weeks.

**Figure 4:**
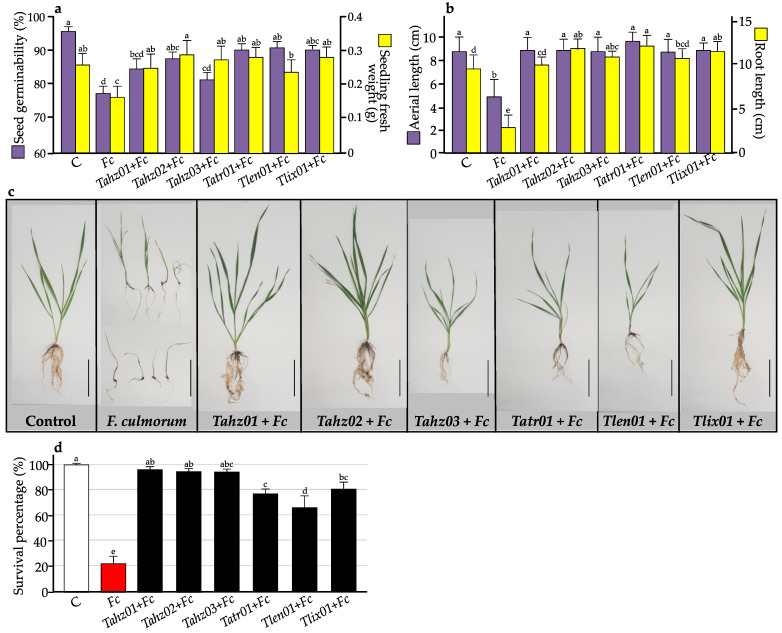
Bioprotection effect of *Trichoderma* strains on wheat plant infested by *F. culmorum* (*Fc*). (**a**) Percentage of germination and fresh seedling weight, and (**b**) aerial section and root lengths, 7 days post-germination in an axenic environment. (**c**) General morphological aspect of plant co-inoculated by protagonists, and (**d**) percentage of plant survival 21 days post germination. C, sterile PDB control. Scale bars in photographs represent 10 cm. (**a**,**b**) correspond to the mean of thirteen plants; (**d**) corresponds to the mean of six biological repetitions, including ten plants. Letters in the same column describe levels of statistical significance (*p* ≤ 0.05); values are means ± SE. Disease symptoms related to virulence of *F. culmorum* are presented with more resolution and details in Figures S4 and S5 in Supplementary Materials.

The first bioassay, based on seed emergence in vitro, showed highly pathogenic behavior of *F. culmorum,* with mycelium surrounding the seed by forming thick and robust hyphae (Figure S4 in Supplementary Materials). This infection blocked seed germination before the appearance of coleoptile and radicle, killing more than 23% of the seeds (Figure 4a). Therefore, the final germination percentage (FGP) was significantly impacted by *F. culmorum*; it ranged from 75% in seeds infested with *F. culmorum* compared to 97% in non-infested seeds (control) (Figure 4a). For seeds that managed to germinate, they were significantly impacted by the aggressiveness of *F. culmorum*, showing the lowest total fresh weight and reduced root and shoot lengths (Figure 4a,b; Figures S3 and S4 in Supplementary Materials).

Treatment with *Trichoderma* isolates showed that they were able to profoundly combat the negative impacts of *F. culmorum*, which is reflected in the suppression of the appearance and development of any *Fusarium* symptoms (Figures S3 and S4 in Supplementary Materials). This results in a significant increase in the final germination percentage for *Tahz02*, *Tatr01*, *Tlix01* and *Tlen01* to levels similar to that of the control and complete suppression of the negative effect of *F. culmorum* on total fresh seedling weight, shoot and root length for all strains. In addition, *Tahz02*, *Tahz03, Tatr01* and *Tlix01* showed a bioprotective and biostimulatory effect at the same time (in the presence of *F. culmorum*) by significantly increasing root length compared to the control (Figure 4b).

The second bioassay carried out in a greenhouse aimed to confirm the protective performance of *Trichoderma* isolates on growing wheat. Results showed that *F. culmorum* severely negatively affected the survival of seedlings (Figure 4d). Only 22% of seedlings had survived and showed reduced growth parameters (shoot/root lengths, fresh/dry total weights, leaf Chl/Flav content and NBI). We note, however, that the protective abilities of *Trichoderma* isolates were expressed differently between our six strains. The strain *Tahz01* had completely protected the seedlings from *F. culmorum,* with neither the survival percentage nor the growth parameters being significantly influenced by *F. culmorum* infection, allowing seedlings to grow normally. In this respect, *Tahz01* is considered to be the best protective *Trichoderma* strain tested in this study. In addition to that, this strain (*Tahz01*) has exhibited a biostimulatory effect in addition to its protective role. This effect was made very clear from the values of the shoot/root lengths, total fresh weight, shoot dry weight and leaf chlorophyll contents, compared to the values of the control seedlings (Figure 3 and Figure 4d). The rest of the strains were partially protective by reducing the aggressiveness of *F. culmorum*. *Tahz02* and *Tahz03* were the most efficient when it came to seedling survival, while *Tatr01*, *Tlen01* and *Tlix01* were less effective (Figure 4d). Concerning the growth parameters, the ecophysiological traits recorded in leaves (chlorophyll content and NBI proxy) were relatively unaffected, while a weak protective action was noted in roots (fresh and dry weights) for *Tahz03*, *Tatr01* and *Tlen01* (Figure 3).

Finally, plants that were diseased by inoculation of *F. culmorum* alone showed a drastic drop in the content of epidermal flavonols (Figure 3i). Interestingly, flavonol abundances were significantly up-regulated when the *Trichoderma* were co-inoculated with *F. culmorum*, except for *Tatr01* and *Tlen01,* whose levels stayed stable and similar to the control. It is plausible that these increases in flavonol productions have a significant impact on the resistance of wheat induced by these *Trichoderma*, as it has been observed in other positive biotic interactions involving *Trichoderma* spp. or symbionts [110,111,112,135,136]. Finally, the synthesis of these secondary metabolites involves the intervention of several specific enzymes (phenylpropanoid pathway). Their regulation also includes accumulation levels, which are ensured by the transcriptomic regulation of their related encoding genes. These genomic regulations will be discussed in greater detail in the next item.

#### 3.4.2. Trichoderma Display Antagonistic Abilities In Vitro against *F. culmorum* Growth

**Figure 5:**
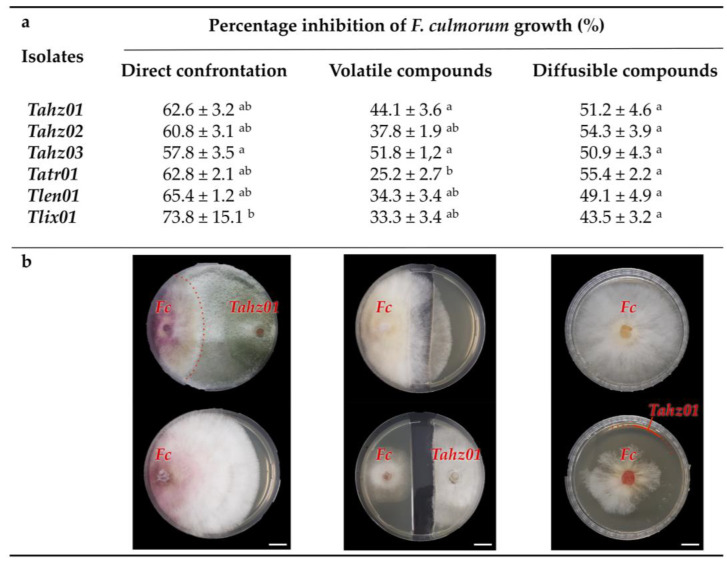
In vitro antagonistic activity of *Trichoderma* isolates against *F. culmorum* on PDA medium after 7 days of confrontation (28 °C). (**a**) Antagonism was highlighted after direct confrontations of mycelia or indirect confrontations with volatile and diffusible *Trichoderma*’s metabolites. (**b**) Photographic examples of antagonistic activities with *T. afroharzianum* (*Tahz01*). Data correspond to the mean of six biological repetitions. Letters in the same column describe levels of statistical significance (*p* ≤ 0.05); values are means ± SE (*n* = 6). Scale bars in photographs represent 1 cm. Antagonism activity of each *Trichoderma* isolate is presented in Figures S5 and S6 in Supplementary Materials.

The antagonistic ability of the six native strains against *F. culmorum* was evaluated using in vitro dual culture assay. All the evaluated strains were capable of reducing *F. culmorum* growth. The inhibition rate (IR) was above 50%, indicating that all of the isolates exhibited high antagonistic activity. The most effective strain was *Tlix01* with 73.8% IR, and that was only significant with *Tahz03,* which showed the lowest IR (57.8%) (Figure 5; Figure S5 in Supplementary Materials). In addition, the results of the determination of diffusible metabolites demonstrated that the growth of *F. culmorum* was inhibited by at least 43.5%. This could be explained by the fast growing ability of *Trichoderma* to quickly colonize the surface and outcompete for resources, producing metabolites that lead to the suppression of the growth of *F. culmorum* [137].

The method used to determine volatile metabolites proved that all tested *Trichoderma* strains were able to inhibit *F. culmorum* development by 25.2% to 51.8%, with a significant difference between *Tatr01* and *Tahz03*, respectively (Figure 5a,b; Figure S6 in Supplementary Materials). *Trichoderma* species produce various secondary metabolites, such as diffusible and volatile organic compounds (VOCs) [138], and among them, a group of more than 500 carbon-based compounds is able to diffuse into the atmosphere and soil and/or is solubilize in water. These compounds are known as microbial volatile organic compounds (mVOCs), and their biosynthesis is dependent on strains and environmental conditions [24,108]. They display multiple biological functions, such as enhancing plant growth and resistance [108,139,140], and are harmful to a broad spectrum of plant pathogens [24,29,140] or both [141]. The molecular diversity of VOCs is remarkable; it includes (thio)alcohols, aldehydes, heterocycles, hydrocarbons, ketones, phenols, thioesters and a plethora of derivatives [142,143]. Our study has demonstrated the ability of the *Trichoderma* isolates to produce volatile and soluble mVOCs, which would weaken *F. culmorum* mycelia development to be vulnerable to hydrolytic enzymes. These mycoparasitic events would lead to the successful inhibition of *F. culmorum* growth, as previously observed in the interaction Trichoderma-*Pyrenophora teres*, the causal agent of barley net blotch [144]. In addition, we do not exclude the possibility that some of the emitted mVOCs are involved in the biostimulation of seed germination and seedling growth, especially in the presence of *F. culmorum*, as the general metabolism of *Trichoderma* is influenced by the roots of its host [145,146], as well as its potential prey including *Fusarium graminearum* [147]. This hypothesis would partly explain the PGP ability of *Tlix01*, where mVOCs could compensate for the absence of IAA-related indolic metabolites in this strain.

**Figure 6:**
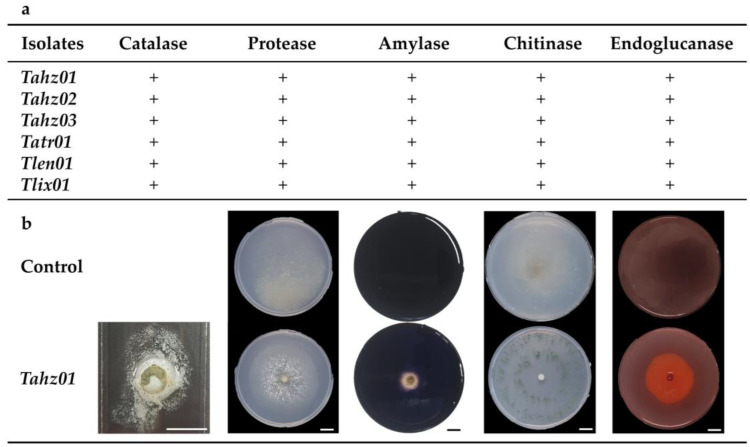
(**a**) Extracellular enzymes produced by the six *Trichoderma* isolates. “+” means a positive result for the enzyme production. (**b**) Photographic examples of the qualitative assay of extracellular enzyme production in the absence of fungal isolate (Control) or in the presence of *Trichoderma afroharzianum* (*Tahz01*). Scale bars in photographs represent 1 cm.

Along the same lines of exploring *Trichoderma* weapons used in the control of *F. culmorum* development, qualitative determination of enzymes, presumably involved in the antagonist activity of these isolates, revealed positive results illustrated by bubbling for Catalases and a halo of degradation inside the mycelial growth zone for Endoglucanases, Proteases, Chitinases and Amylases production (Figure 6).

These enzymes are produced as part of *Trichoderma* metabolism to obtain nutrients and to defend themselves against other microorganisms. Catalases, which are oxidative enzymes, have been shown to be involved in its biocontrol activity against *F. culmorum*, while the hydrolytic enzymes are secreted to break down *Fusarium* cell walls to get access to nutrients and help thus to reduce the severity of *Fusarium* infections. We hypothesize that these lytic enzymes (which are primarily involved in the extracellular digestion of *Trichoderma*) act in concert with various antagonistic metabolites (mVOCs) to take part in the mechanisms that drive *Trichoderma*’s hyperparasitism activities against *F. culmorum*.

#### 3.4.3. Trichoderma Strains Stimulate the Wheat Immune Responses

##### General Consideration

**Figure 7:**
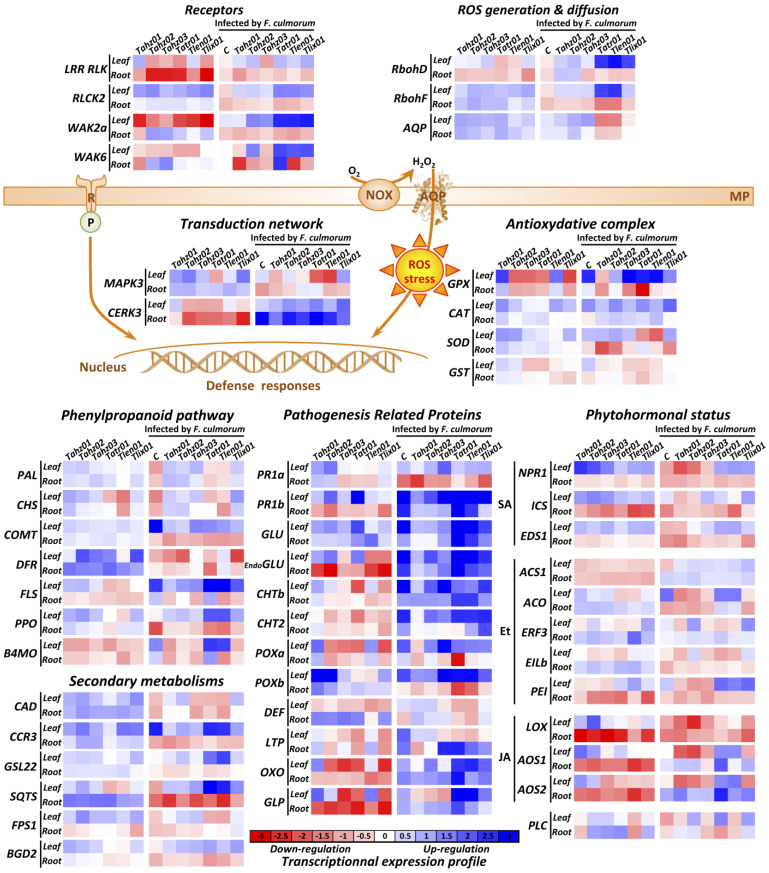
Defense-related gene modulation in wheat roots and leaves following fungal inoculations with *Trichoderma* isolates alone or in combination with *F. culmorum*. Organs sampled for molecular analyses came from plants used for physiological and morphological analyses. Transcript levels for each gene were estimated using real-time *q*RT-PCR analyses and normalized by the expression of three housekeeping genes. Relative transcript abundance rates were obtained by the 2^−ΔΔCT^ method. Data correspond to the mean of three independent biological experiments; each biological repetition corresponds to a mix of 5 plants. Colors represent the levels of transcriptional expressions (blue, up-expression; red, down-expression, compared with healthy samples treated with sterile PDB). NOX, NADPH oxidase; AQP, Aquaporin. Statistical analyses were detailed in Table S2 in Supplementary Materials. Gene acronyms and related primers used for *q*PCR amplification are given in Table S1 in Supplementary Materials. SA, salicylic acid; Et, Ethylen; JA, Jasmonates.

As for the biostimulation of plant defenses, all isolates significantly modulated the transcriptional expressions of genes involved in the system-wide immunity of wheat (all *p*-values < 0.05) (Figure 7; Table S2 in Supplementary Materials). Plant defenses are remarkably diverse, and the ways in which they are regulated are also remarkably varied and complex. They involve multiple pathways (viz., PR proteins, secondary metabolites, parietal reinforcements, reactive oxygen species (ROS)) inherent to the recognition of bioagents, which, in our study, correspond to both pathogenic (*F. culmorum*) and beneficial agents (*Trichoderma* spp.). The accumulation of plant defenses is one among cellular responses that are set off by the recognition of external agents or substances and the self-recognition of plant molecules released by itself during the interaction such as stress phytohormones (SA, JA, Et, IAA, etc.), ROS with the hydrogen peroxide (H_2_O_2_), and/or various metabolites coming from microorganisms living on the host plant. The defense markers we selected for this study are common and cover several defense pathways (PR-proteins, phenylpropanoids, sesquiterpenoids, oxidative stress, recognition and signal transduction).

Molecular analysis was carried out on the roots and leaves of 21-day-old seedlings grown from seeds that were coated with the six *Trichoderma* isolates. An overview of transcriptional data showed that all targeted molecular markers were significantly modulated by the application of fungus (*Trichoderma*/*Fusarium*) independent of analyzed tissues. The modulations are highly diversified, with positive and negative kinetics reflecting the systemic regulation of the defense responses established by the host, which perpetuate over time during plant growth. These physiological features highlight the complexity and richness of the molecular responses that the plants deploy to provide a general resistance phenotype correlated to the BCA capacities of *Trichoderma* or the disease susceptibility caused by the virulence of *F. culmorum*.

Another general observation is that *Trichoderma*, when co-inoculated with *F. culmorum*, significantly enhanced the expression of some of the positive modulations that were recorded in the case of inoculation with only *Trichoderma* or even completely reversed other transcriptional expression trends induced by *F. culmorum* when inoculated alone (down- vs. up-regulations, and vice versa). These cellular responses clearly demonstrated that *Trichoderma* strains triggered deep and long-lasting transcriptional reprogramming of genes involved in plant immunity of wheat seedlings that were infected with *F. culmorum*. The reprogramming concerned all cellular processes: recognition (e.g., *LRR*, *RLK*, *RLCK* and *WAK6*), transduction pathways (*CERK3*), oxidative burst (ROS generation with *RbohD* and *-F*, and the antioxidant system with *GPX* and *CAT*), accumulation of defenses (secondary metabolisms with *PAL*, *CHS*, *GST* and *COMT*, as well as PR-proteins with *DEF* and *LTP*), and modulation of phytohormonal balances (*ACO* and *AOS2*).

When analyzing transcriptional responses at the *Trichoderma* isolates level, it is interesting to note that *Tatr01* and *Tlen01* exhibited contrasting profiles to those observed with the rest of the isolates. This was significant for genes involved in the recognition process and transduction pathways (e.g., WAKs, MAPK3, *WSF* and *NFLX*), oxidative stress (*RbohF*, *AQP*, *GPX*, *SOD* and *GST*), defense metabolites (*PAL*, *FLS*, *PPO*, *CCR3*, *GSL22*, *SQTS*, *CHS*, *CCR3*, *GSL22*, *PPO*, *PR1b*, *GLU*, and *OXO*) and phytohormonal balances (mainly Et- and JA-dependent pathways with *ACO*, *LOX*, *AOS1* and *-2*). These two isolates (*Tatr01* and *Tlen01*) were protective, similar to the other isolates, against the emergence of morbid disease symptoms (root and leaf rots) caused by *F. culmorum* (Figure 4b; Figure S3 in Supplementary Materials) but not against growth delays due to the presence of *F. culmorum* on plants (Figure 3). Although these are correlations, this finding may be explained by the fact that *Tatr01* and *Tlen01* have affected the transduction pathways inherent to plant growth and immunity in a different way than other isolates. This data is a prerequisite for guiding the selection of strains to be developed in the field. To our knowledge, such contrasting strain-dependent modulations in the instruction of different physiological functions are rarely demonstrated or further discussed in wheat interacting with BCAs in general and with *Trichoderma* in particular.

##### *Trichoderma* Isolates Stimulate the Systemic Accumulation of Wheat Defenses

Plant defenses are expressed sequentially, temporally and spatially. *Trichoderma* inoculation in the presence or absence of *F. culmorum* modulated all the targeted markers. Based on our transcriptomic results, we proposed a putative model of the long-term molecular interaction between above-ground and underground parts of wheat seedlings grown from seeds coated with the six *Trichoderma* isolates.

The expression levels of the defenses encoding genes resulting from the recognition of the agents by plant receptors (R) would participate in triggering both the root colonization of *Trichoderma* and the systemic modulation of defenses accumulation which then leads to the resistance of wheat against *F. culmorum*. Different molecular modalities of *Trichoderma* spp. plant recognition and its related defense elicitation have been described, for example, involving the Sm1/Epl1 elicitors [148,149]. Consistent with several transcriptomic and proteomic studies [30,150], the six *Trichoderma* isolates induced the modulation of several plant resistance (R) genes, which was correlated with the activation of a wide range of plant defenses and a high physiological level of resistance. It is then also plausible that this resistance acquisition should be extended to pathosystems other than *F. culmorum* (and of which *WAK2* would be one of their recognition systems [151]), such as *Blumeria graminis* f. sp. *tritici* (*LRR RLK*) [152], *Puccinia triticina* (*WAK6*) [153], *Rhizoctonia cerealis* (*RLCK*) [154], and those whose related receptor-like kinases encoding genes are significantly up-regulated by certain *Trichoderma* strains alone and/or in the presence of *F. culmorum*. However, the enhancement of the effector-triggered resistance relies on the highly specific interaction between the microorganism’s effectors and the potential plant’s receptors (R). The fact that we observe diversified transcriptional patterns of the (R)-gene responses between strains suggests that each isolate has deployed specific effectors that interfere with the plant effector-triggered immunity (ETI). It will be very informative to further characterize the effectors involved in the interaction of the most efficient *Trichoderma* strains on wheat.

Receptors perceive and process signals from invading microorganisms in diverse pathological and beneficial systems. Among the subsequent cellular responses induced by our *Trichoderma* isolates, we observed an increase in transcription of wheat respiratory burst oxidase homologues (i.e., NADPH oxidase with the *Rboh-D* and *-F* subunits) and proteins involved in ROS diffusion (*AQP*) and scavenging (antioxidative complex). These modulations mainly involved up-regulations that led to the modification of the global oxidative state of plants that were linked to a generation of ROS. They occurred in the roots (except for *Rboh-D*) and in the leaves, where they were correlated to the modulation of the SA-dependent markers. Indeed, SA is a key mediator in eliciting an oxidative burst in challenged plants, thus regulating antioxidant metabolism. In this respect, these results agree with those which were reported in wheat inoculated with different *Trichoderma* species where the application of *Trichoderma* triggers both oxidative stress and systemic defense pathways in wheat seedlings [155].

Besides the toxic and antimicrobial aspects of ROS, hydrogen peroxide (H_2_O_2_) is a key player in the complex signaling network of plant responses to abiotic and biotic stresses. It is, therefore, essential to maintain ROS at sub-toxic levels, which reflects a delicate balance between ROS production (involving the activation of ROS-generating enzymes under stress and the unavoidable production of ROS during basic cellular metabolism) and ROS elimination pathways. Here, our experiments demonstrated that *Trichoderma* induced persistent and latent oxidative stress in wheat, but this stress was counteracted by the production of antioxidant enzymes (*CAT* and *SOD*) that then reduced the oxidative damage linked to the high chemical reactivity of ROS. In addition to their role in the elimination of *F. culmorum*, these ROS and in particular H_2_O_2_, would also participate in the changes of the plant’s phytohormone balances rather than in the elimination of *F. culmorum*, which, subsequently, would notably activate the accumulation of defenses. Furthermore, H_2_O_2_ is a relatively stable molecule over time, and it has the distinctive feature of freely diffusing through the plasma membrane. However, its diffusion in tissues should and could be regulated by peroxyporins. Peroxyporins are specific aquaporins (AQP) that belong to the Major Intrinsic Protein (MIP) family [156]. Aquaporins are studied in a plethora of cellular physiological pathways due to their ability to regulate water transport in biological systems. However, peroxyporins play a relevant role in controlling H_2_O_2_ permeability, and they ensure the ROS outcome during oxidative stress, including that related to the initiation of the disease immune responses by plant cells [157]. AQP expressions were modulated by all isolates, both in roots and leaves, implicating them in the diffusion of many solutes and also H_2_O_2_, reinforcing the hypothesis that ROS-dependent signals may have influenced the overall resistance responses of the plant. It is, therefore, interesting to correlate the cellular pathway inherent to the production, diffusion and pro-oxidative control of ROS with the resistance of wheat against *F. culmorum* by *Trichoderma*.

Concomitant with oxidative stress, the strong induction of various defense pathways is a key plant defense strategy to limit pathogen development. It involves the production of a large panel of “biopesticide” secondary metabolites, including phenylpropanoids, terpenoids (*SQTS*, *FPS1*), and enzymes involved in the biosynthesis of monolignol (Cinnamyl alcohol dehydrogenase, *CAD*, and Cinnamyl coenzyme reductase 3, *CCR3*), which participate in cell wall strengthening and callose apposition (*GSL22*). These genes are mainly induced in leaves and roots, and a few of them may also be repressed but with similar modulation rates.

PAL and CHS are key enzymes of the phenylpropanoid biosynthesis pathway, providing precursors for the synthesis of antimicrobial phenolics such as lignins, flavonoids, isoflavonoids and coumarins. Furthermore, these enzymes are de facto involved in the SA defense pathway since SA is synthetized from phenolic derivatives. However, more broadly, it should be emphasized that several genes involved in the biosynthesis of flavonoids and terpenoids were up-regulated by several *Trichoderma* isolates, thus reinforcing the relevance of such a pathway during the Wheat–*Trichoderma* interaction leading to efficient systemic resistance against *F. culmorum*. These transcriptional up-regulations are correlated with the leaf epidermal flavonol contents recorded in the leaves of plants treated with both *Trichoderma* isolates alone or with *F. culmorum* (Figure 3i).

The intertwining of enzymatic markers involved in different metabolic pathways under stress seems to contribute to disease resistance. For instance, *CAD* and *CCR3*, two key enzymes involved in lignin biosynthesis, interplay in apoplasm with the oxidative enzymes Peroxidase (*POX*) and Polyphenol oxidase (*PPO*) in the presence of ROS (O_2_°^−^, °OH) and H_2_O_2_ to catalyze the formation of lignins and their crosslinking with other oxidative phenols, proteins and molecular components of the cell wall. These biochemical interactions contribute to the reinforcement of the cell wall and to the callose apposition process itself, assisted by Glucan synthase-like activities (*GSL22*). Interestingly, these key enzymes display significant up-coregulation in the roots and leaves elicited by *Trichoderma* alone or with *F. culmorum*. In addition, their accumulation patterns are analogous to Superoxide dismutase (*SOD*) enzymes that are responsible for the dismutation of superoxide radicals into oxygen and H_2_O_2_. A contrario, the expression profile of these enzymatic markers was significantly inhibited in the roots of *Trichoderma*-*F. culmorum* co-inoculated plants, compared to *F. culmorum* alone. These expressions are possibly related to the toxic impact of certain enzymes on the development of microorganisms in general and on the long-term *Trichoderma* colonization in roots in particular. For example, PPO activity oxidizes phenols into quinones, which are more toxic to microorganisms than the original phenolic compounds [158]. Likewise, POXs belong to a large multigene family that participates in several physiological processes, such as the synthesis of phytoalexins, lignin and suberin, the cross-linking of cell wall components and the rapid synthesis of ROS derivatives by oxidative burst contributing to cell death [159]. All these molecular responses provide a physical barrier to and/or substantially limit pathogen spread and invasion throughout the plant. Finally, their inhibition in co-treated root correlates with the down-regulation of the SA- and JA-dependent signaling pathways, possibly to finely control the abundance of related defenses in the roots which are in direct contact with the fungal protagonists.

As for the pathogenesis-related (PR) proteins, which are grouped into 17 families based on their sequence and biological activities, they are components of an early response toward pathogen infection, playing a key role in pathogen-spread limitation [160]. Phytohormones (SA, JA and Et) with ROS are involved in the signal transduction network and the accumulation of PR-proteins primed by *Trichoderma* spp. [32,161,162,163]. In our assay, PR-protein transcriptional accumulations were significantly activated in leaves challenged by *Trichoderma* alone, while these expressions were strongly potentiated in the leaves and also in roots in the case of co-treatment *Trichoderma*-*F. culmorum*. Finally, co-regulations of PR-proteins, as well as other defense markers, could be pointed out mainly in the leaves challenged by *Trichoderma* alone; it concerns *CHTb*, *POXa*, *DEF*, *LTP, OXO* and GLP, with *LRR-RLK*, *CERK3* and *GPX*. Interestingly, these expression patterns are quite connected to the modulation of the JA-dependent signaling pathways (*AOS1* and *AOS2*), knowing that among these markers *CHTb* (PR-4), *GLP* and *AOS2* are also positively induced by fungal pathogens through the JA signaling pathway [164]. To our knowledge, these markers do not seem to be linked to a common signaling pathway. However, the fact that these profiles are correlated may underline key regulatory points in the establishment of certain disease resistance, such as those involved in the role of the LRR-RLK receptor against *Blumeria graminis* f. sp. *Tritici*, the agent of wheat powdery mildew.

Finally, we do not omit that some defenses are weakly/unmodulated or significantly inhibited during the interaction in roots in similar patterns to those recorded with *F. culmorum* alone (e.g., *C4L*, *CCR3*, *GSL22*). It is plausible that these defenses remain under the control of the pathogen, even with the strong regulations that *Trichoderma* exerted on the global signaling pathways in wheat seedlings. However, these modulations could also be related to the successful, long-lasting *Trichoderma* establishment on or in these plant organs. We can then assume that certain specific defenses are “effective” against *F. culmorum* (correlated to their down-regulation) and that they would be equally toxic to *Trichoderma*. For example, the expression profiles of certain PR-proteins (*PR1b*, *GLP*, *OXO*), key biosynthetic steps of secondary metabolites or the sextet (*SOD*-*PPO*-*POX*-*CCR3*-*CAD*-*GSL22*) would fit this hypothetical scenario. As demonstrated, even though these defenses can be up-regulated rapidly by *Trichoderma* treatments [30,116], their expression is reprogrammed over time to perpetuate an optimal mutualistic interaction that the plant maintains with its beneficial microorganisms. From this perspective, the mechanisms deployed by *Trichoderma* to persist in the root system as an avirulent symbiont are not yet fully revealed. It is highly probable that it depends on “virulence effectors” involved in plant defense suppression, whose nature and mode of action remain to be deciphered.

##### *Trichoderma* Isolates Influence the Wheat Phytohormone Balances

Root penetration by *Trichoderma* is reported to transiently induce the expression of defense-related genes and the production of antimicrobial compounds. Our results demonstrated here imply that root colonization by *Trichoderma* after seed coating application maintained the transcription of many defense-related genes at significant levels for a relatively long period of time (21 days). These observations suggest that the long-term response of wheat to *Trichoderma* may involve phytohormone signaling.

In addition to the primary mechanisms of elicitor recognition by plant receptors, complex phytohormonal adjustments occur that supplement or potentiate the primary signals of direct microbial recognition. It is unanimously recognized that beneficial agents in general, and *Trichoderma* in particular, have the potential to profoundly influence the phytohormonal balances of plants involved in local and systemic signaling responses to stress. These balances include salicylic acid (SA)- and jasmonate (JA)/ethylene (Et)-mediated signaling responsible for the Systemic Acquired Resistance (SAR) and Induced Systemic Resistance (ISR), respectively, making plants resistant to a broad spectrum of pathogens [116,165,166]. Generally, SAR is induced by fungal biotrophic pathogens, and ISR usually relies on the interaction of plants with symbionts or necrotrophic microorganisms. However, *Trichoderma* spp. are reported to engage several hormonal-signaling pathways to instruct the elicitation of disease resistance in plants. They act in synergy and in balance with each other, and they depend closely on the strains, the plant genotypes, the environment (including the challenged pathogen) and the temporal modalities of the interaction [116,167,168]. We explore these balances here through the SA- and JA/Et-mediated signaling pathways.

Concerning the SA-dependent pathway, *Trichoderma* isolates the negatively modulated *NPR1* and *ICS* in the roots, whether inoculated alone or with *F. culmorum*. These modulations are quite diversified in the leaves, being positive for simple *Trichoderma* inoculation or with inhibitions or activations for co-inoculation with *F. culmorum,* but they are strain-dependent. The down-regulation of the SA pathway markers recorded in our work should not be opposed to the key role played by SA in the early stages of root colonization by beneficial microorganisms where SA accumulation and/or transcriptional up-regulation of SA pathway-dependent genes are reported. Early phases involve plant responses that resemble those induced by biotrophic pathogens present in the intercellular spaces, similar to many beneficial agents [169]. These first phases are correlated with transitory accumulations of SA that may be responsible for the accumulation of plant defenses. In an interaction involving *Trichoderma*, these common physio-molecular responses would prevent the fungus from entering the apoplastic space of the epidermal and cortical tissues towards the root vascular system [170]. Here, our study targeted the defense responses of 21-day-old wheat seedlings. This specific temporality of our analyses would then explain the weak modulation and inhibition of several defenses observed in roots and could also be related to the successful and long-lasting establishment of *Trichoderma* on these plant organs. Such partial suppression of SA-dependent responses in plants also appears to be necessary for the onset of symbiotic associations [171,172]. Moreover, these organ- and strain-specific transcriptional responses are very informative. They highlight that isolates behave and/or are perceived differentially by the plant, which is dependent on their presence both alone or with their plausible prey (*F. culmorum*). This demonstrates the complexity of the interactions that beneficial agents establish with plants in the presence or absence of other protagonists and whose outcomes are closely dependent on the biotic and abiotic environments where these interactions take place.

The expression pattern of the JA/Et pathway markers allowed us to discover some interesting correlations. Ethylene (Et) is a phytohormone that regulates plant growth, development and senescence, but low Et concentrations in the root system are correlated with higher shoot growth. These molecular responses (along with the ecophysiological traits we recorded) have to be linked to the fact that many PGP microorganisms from the rhizosphere could reduce the levels of 1-aminocyclopropane-1-carboxylic acid (ACC), which is the precursor metabolite of Et, by producing the enzyme ACC deaminase (ACCD). *Trichoderma* strains own this feature of Et regulation [173], regulating the level of ET in conjunction with other phytohormones involved in the growth process (GA3, CKs) [124,174]. This could be explained by the fact that *Tatr01* and *Tlen01* have induced an overexpression of *ACO* genes, which is correlated with a reduction in root growth (Figure 3d–f). In our analysis, it is interesting to note that the expression of *ACS* and *ACO* genes, which encode for enzymes involved in the last two key steps of the Et biosynthesis in plants, is apparently desynchronized between biological variations (i.e., inoculation alone or combined with *F. culmorum*). Similarly, the expression of regulatory factors known to be related to ethylene (Et)-mediated signaling (*ERF3*, *PIE*) have shown highly divergent profiles. These contrasting transcriptional profiles have not been over-interpreted to avoid making wrong conclusions, but they could plausibly be attributed to several global regulations. These regulations are generated from long-term transcriptional reprogramming of Et-signaling influenced by the general phytohormonal balances of plants, to which *Trichoderma* contributes with its own phytohormone-like production.

The same goes for the jasmonates (JA)-mediated signaling pathway. Jasmonates are a family of molecules derived from the lipid metabolism and subsequent peroxidation of membrane lipids caused by an oxidative burst. Jasmonates, in cooperation with other phytohormones (including SA), play an active role in the regulation of plant innate immune responses by controlling a cascade of complex physio-biochemical changes (metabolite accumulation and enzymatic activities) within the host plant, which then contributes to the development of resistance/tolerance against a wide range of invaders [175,176]. In addition, jasmonates influence the regulation of mutualistic interactions between plant and their associated microbes in the roots and aerial parts. In this respect, the fact that *Trichoderma* would inhibit JA-dependent regulatory pathways (*LOX*, *AOS1* and *-2*) in the roots of wheat plants further mirrors the fact that mycorrhizal colonization is stimulated by low concentrations of jasmonates rather than higher ones. Concerning leaves, plant systemic resistance induced by *Trichoderma* spp. correlate with a significant increase in JA-related markers, which is consistent with several works [116,177]. However, a complete re-organization of the JA-marker transcripts accumulations occurs when *Trichoderma* is co-inoculated with *F. culmorum*, while *F. culmorum* alone displayed inhibitor activity on these JA-markers. These trends suggest the possible existence of cooperative interactions between the mutualistic plant symbiont (i.e., *Trichoderma*) and the roots of the host plant in response to *F. culmorum* infection through the emission of specific JA derivative metabolites such as the methyl jasmonates (MeJA). Both molecules are natural inducers of plant resistance and their concomitant application with *Trichoderma* spp. significantly improves the induction of plant immune responses [175,177]. In addition, MeJA also exhibits significant antimicrobial activities by inhibiting spore germination and mycelial development of various plant pathogenic fungi [178,179,180].

These modulations need to be analyzed in a more holistic sense. We have to mention that most of the studied *Trichoderma* species colonize either the root surface or the interior as endophytes [181,182], including in wheat [183]. However, within the soil fungal communities, *Trichoderma* spp. has a relatively low abundance in both agricultural [184,185] and natural ecosystems [186,187]. The act of seed coating guarantees the presence of *Trichoderma* in wheat tissues over time to ensure a long-lasting and systemic elicitation of plant responses. Among the early signaling-induced events related to the host-*Trichoderma* recognition, high levels of SA, Et and JA were observed in different parts of the plant [188,189]. While several studies have focused on the early (i.e., hours or days) and systemic elicitation of *Trichoderma* when inoculated on roots and/or leaves by transcriptomic or proteomic analysis, our study has the originality of providing new insights about these root and leaf responses when seeds are coated with *Trichoderma*. The isolates thus have time to acclimatize and settle in their edaphic environment while potentially colonizing the growing plant organs (i.e., coleoptiles, then all or part of the young seedling, including the roots). The result after 21 days of interaction in a greenhouse shows that plant response patterns related to phytohormonal defense regulations are highly diversified between pathways (SA and JA/Et) and closely dependent on the applied strains. These responses are very complex to interpret, but growth and immune performances appear to be associated with the simultaneous transcript expression of SA and JA/Et defense-related genes, as well as with the expression of an oxidative burst and accumulation of antimicrobial metabolites. SA- and JA/Et-dependent signaling are often depicted as antagonistic, but here, their synergistic overlap seems to occur instead, as previously reported for some pathosystems [116,190,191]. However, although the induced SAR and IRS seem compatible in our study, the signaling pathways leading to plant resistance and growth are different and distinctly strain dependent. Furthermore, the complexity of the transcriptional responses observed would plausibly be linked to the interaction of two pools of phytohormones emitted during plant/*Trichoderma* interaction, i.e., the first emitted by the plant in response to the recognition and settlement of *Trichoderma* in the root, and the second emitted by the fungus to manipulate its (biotic) environment, and which, here, would fully integrate the signaling pathways of the plant. In other words, the global and systemic hormonal regulations observed in this study would be conditionally linked to the ability of *Trichoderma* to produce several phytohormones (such as ABA, CKs, GAs, SA, Et and IAA). The level of these phytohormones is strain-specific, and their cooperation synergistically modulates plant disease resistance and growth promotion [192].

Therefore, and consistent with what has been observed in other studies on tomato [193], cucumber [194] or olive [32], this evidence further underlines the key influence of *Trichoderma* on the overall phytohormonal balances that interconnect plant defense and development responses. The transcriptional expression levels of the markers studied here did not allow us to identify which phytohormone-likes are mainly produced by the different *Trichoderma* strains, nor the way in which they cooperate to reach this effect on plant growth and defense promotion. Other ongoing research will quantify the phytohormone-like compounds produced by these beneficial isolates in order to identify their potential mode of action during the molecular dialogues of the defensive protection process in wheat.

### 3.5. Long-Lasting Root Installation of Trichoderma Isolates for a Sustainable Plant Protection

It should be noted that the physiological responses recorded here are expressed in the roots and leaves of wheat plants harvested after three weeks under greenhouse conditions and that the fungal treatments were carried out only on seeds.

Several events can explain the durability of the signal over time. Each *Trichoderma* isolate was detected in the roots of plants coming from seeds treated with that same strain (Figure S7 in Supplementary Materials). The same goes for the pathogen, which was only detected in the roots of plants originating or not from seeds previously treated with *Trichoderma* isolates. Regarding leaves, no PCR amplification signal was detected for all *Trichoderma* isolates and *F. culmorum* was detected in plants treated only with this pathogen. However, it is plausible that the isolates are also present on/in the aerial parts but at levels not measurable by the analytical approaches used here and/or that endophytic populations mask the signal. In this respect, these potential trace populations that would be controlled by the immune performance of plants stimulated by *Trichoderma* could conceivably still have a positive influence on the foliar defense responses by intensifying the primary systemic signals emitted by the roots during the interaction with *Trichoderma* isolates.

The prolonged presence of the fungal protagonists should thus have an influence (individually or synergistically) on the intrinsic “immune memory” of plant cells whose inhibitory feedback regulations have not yet been fully deployed. The combination of these two events can temporarily reduce the vitality of the growth of the plants. Plants actively prioritize defense over growth, and this prioritization and the associated growth trade-offs are regulated by crosstalk between plant hormones [195,196]. In our experimental context, these negative collateral effects were not observed with any of the beneficial interactions, and *Tahz01*, *-02* and *-03*, and *Tlix01* significantly improved plant growth dynamic (Figure 3). Therefore, this data is crucial to determining the choice of strains whose positive effect is closely linked to their ability to develop BCA and PGP capabilities simultaneously.

Finally, in our study, where in planta experiments were conducted in a growth chamber, *F. culmorum* was traceable in diseased plants, as well as in symptomless plants that were previously treated with each *Trichoderma* isolate. This data is of great significance because the fact that *Trichoderma* persistently colonizes the roots alongside the pathogen suggests the relevance of using these agents as a form of biocontrol, particularly for a pathogenic population, and/or in the modulation of the expression of pathogenicity genes that instruct the level of pathogen’s aggressiveness. The molecular dialogues that regulate cellular responses in each of the interacting fungal protagonists open up many remarkable exploratory avenues, which will be studied in the future.

## 4. Conclusions

To conclude, this study was conducted to isolate local *Trichoderma* spp. from rhizospheric soil of an old wheat variety (cultivated in an organic farming system) in order to use them as Plant Growth Promoting Fungi (PGPF) and Biocontrol Agents (BCAs) against *F. culmorum*, the agent responsible for Fusarium seedling blight disease in wheat (FSB). Of all the *Trichoderma* isolates, six strains were tested in this study. The results showed that seed coating with *Trichoderma* strains before sowing was efficient in promoting plant growth by increasing shoot/root length, total fresh/dry weight, total chlorophyll content and nitrogen plant status (NBI). In addition, strains appeared to be efficient biological control agents in their capacity to inhibit *F. culmorum* growth in vitro and by reducing disease severity under controlled environmental conditions. Moreover, the strains were able to induce systemic and long-lasting immune performance in the roots and leaves of 21-day-old seedlings. These physiological characteristics correlated with profound transcriptional changes in the gene encoding for plant defenses and reprogramming of hormonal balances, leading to simultaneous expressions of SA- and JA/Et-dependent pathways. The spatio-temporal coordination of these cellular responses conferred systemic resistance to wheat seedlings where the levels of its protective effect depended on the *Trichoderma* strain tested.

This research allowed us to explore the local *Trichoderma* strains which colonize the wheat rhizosphere and examine their performance as PGPF and BCAs. Their successful use here needs to be followed up by further analysis to fully understand the nature of the molecules (diffusible and volatile metabolites) involved in the biocontrol activity, as well as their impact on wheat growth and resistance performances in the field. In this respect, new studies focused on deciphering the molecular dialogue between the protagonists (*Fusarium*, *Trichoderma* and wheat) are in progress to better understand and valorize the isolated *Trichoderma* strains, which will then potentially guide their industrial application.

## Figures and Tables

**Table 1:** Species details and their GenBank accession numbers used in phylogenetic analyses.

Species	Strain/Isolate	ITS	*tef1*	*rpb2*
*T. afroharzianum*	*Tahz01* ^a^	OP970986	OR039793	OR039787
*T. afroharzianum*	*Tahz02* ^a^	OP970987	OR039794	OR039788
*T. afroharzianum*	*Tahz01* ^a^	OP970990	OR039795	OR039789
*T. atrobrunneum*	*Tatr03* ^a^	OP970988	OR039796	OR039790
*T. lentinulae*	*Tlen01* ^a^	OP970991	OR039797	OR039791
*T. lixii*	*Tlix01* ^a^	OP970989	OR039798	OR039792
*T. afroharzianum*	Tafum1	MT102401.1	MT081431.1	MT118246.1
*T. afroharzianum*	Tri-1	MT793748.1	OP102131.1	OP102132.1
*T. atrobrunneum*	T39	MG952890.1	KX632628.1	KX632571.1
*T. lentinulae*	CGMCC 3.19699	MN594478.1	MN605887.1	MN605876.1
*T. lentinulae*	CGMCC 3.19847	MN594469.1	MN605878.1	MN605867.1
*T. lixii*	C.P.K. 1934	EF392746.2	FJ179573.1	MT587315.1
*T. lixii*	TLiC8	MT434003.1	MT587276.1.1	MT587315.1
*T. reesei*	CBS999.97	CP020878.1	CP020876.1	CP017984.1
*T. atroviride*	TRS18	KJ786757.1	KJ786839.1	KP009061.1
*T. longibrachiatum*	Tloum3	MT102396.1	MT081437.1	MT118251.1

^a^ The newly generated sequences and their respective codes used in this work.

**Table 2:** Plant growth-promoting traits of *Trichoderma* isolates after 5 days of fungal growth.

Strains	Phosphate Solubilization	Indole-like Compounds Production (μg·mL^−1^)	Ammonia Production (μg·mL^−1^)	HCN Production
***Tahz01***	-	4.16 ± 1.76 ^b^	16.7 ± 1.2 ^a^	-
***Tahz02***	-	12.43 ± 6.56 ^a^	15.9 ± 0.3 ^a^	-
***Tahz03***	-	0.93 ± 1.35 ^d^	14.4 ± 1.4 ^a^	-
***Tatr01***	-	1.7 ± 0.2 ^c^	14.1 ± 1.5 ^a^	-
***Tlen01***	-	1.1 ± 1.81 ^ed^	14.8 ± 1.1 ^a^	-
***Tlix01***	-	-	14.4 ± 2.4 ^a^	-

Values are means ± SE (*n* = 3). Distinct letters in the same assay indicate significant differences (*p* ≤ 0.05). A “-” corresponds to an absence of phosphate solubilization and HCN or indole-like compound productions.

## Data Availability

Data is contained within the article or supplementary material.

## Supplementary Materials

The following supporting information can be downloaded at: https://www.mdpi.com/article/10.3390/microorganisms11061512/s1, Figure S1: Tunisian site of origin of the *Trichoderma* strains used in this work; Figure S2: Macroscopic and microscopic aspects of the *Trichoderma* isolates; Figure S3: Root and leaves disease symptoms in wheat (Khiar variety) caused by *Fusarium culmorum*; Figure S4: Bioprotection effect of the six *Trichoderma* isolates against *F. culmorum* infestation on wheat (“Khiar” variety) in axenic environment; Figure S5: Direct mycelial confrontation between *Trichoderma* isolates and *F. culmorum*; Figure S6: Indirect antagonistic activity related to emission of volatile and diffusible organic compounds by *Trichoderma* isolates against *F. culmorum*; Figure S7: Qualitative molecular characterization of the presence of *Trichoderma* or *F. culmorum* isolates in the aerial and underground parts of wheat plants 21 days after seed treatment with *Trichoderma* and/or *F. culmorum* isolates; Table S1: Primers used for qPCR amplification of the wheat defense-encoding genes; Table S2: Statistical analyses of the transcript accumulation induced by *Trichoderma* isolates and/or *F. culmorum*.

## References

[1] Huffman W.E., Evenson R.E. (2001). Structural and productivity change in US agriculture, 1950–1982. Agric. Econ..

[2] Weese D.J., Heath K.D., Dentinger B.T., Lau J.A. (2015). Long-term nitrogen addition causes the evolution of less-cooperative mutualists. Evolution.

[3] Vaccino P., Laino P., Limonta M., Gerna D., Vaccino P. (2015). Morpho-physiolological and qualitative traits of a bread wheat collection spanning a century of breeding in Italy. Biodivers. Data J..

[4] Slama A., Ben Salem M., Ben Naceur M., Zid E. (2005). Les céréales en Tunisie: Production, effet de la sécheresse et mécanismes de résistance. Sécheresse.

[5] ONAGRI (2016). Observatoire Nationale de l’Agriculture, Annuaire Statistique.

[6] Gargouri S., Hajlaoui M.R., Guermech A., Marrakchi M. (2001). Identification des espèces fongiques associées à la pourriture du pied du blé et étude de leur répartition selon les étages bioclimatiques en Tunisie. Bull. OEPP/EPPO Bull..

[7] Wiese M.V. (1987). Compendium of Wheat Diseases.

[8] Hollaway G.J., Evans M.L., Wallwork H., Dyson C.B., McKay A.C. (2013). Yield Loss in Cereals, Caused by *Fusarium culmorum* and *F. pseudograminearum,* Is Related to Fungal DNA in Soil Prior to Planting, Rainfall, and Cereal Type. Plant Dis..

[9] Antalová Z., Bleša D., Martinek P., Matušinsky P. (2020). Transcriptional analysis of wheat seedlings inoculated with *Fusarium culmorum* under continual exposure to disease defense inductors. PLoS ONE.

[10] Khemir E., Chekali S., Moretti A., Gharbi M.S., Allagui M.B., Gargouri S. (2018). Survival of *Fusarium culmorum*, causal agent of foot and root rot of cereals, on wheat, barley and oat residues in Tunisia. Ann. L’INRAT.

[11] Simpson D.R., Thomsett M.A., Nicholson P. (2004). Competitive interactions between *Microdochium nivale* var. *majus*, *M. nivale* var. *nivale* and *Fusarium culmorum* in planta and in vitro. Environ. Microbiol..

[12] Li X., Zhang J.B., Song B., Li H.P., Xu H.Q., Qu B., Dang F.J., Liao Y.C. (2010). Resistance to Fusarium head blight and seedling blight in wheat is associated with activation of a cytochrome P450 gene. Phytopathology.

[13] Pirgozliev S.R., Edwards S., Hare M.C., Jenkinson P. (2003). Strategies for the control of Fusarium head blight in cereals. Eur. J. Plant Pathol..

[14] Dal Bello G.M., Mónaco C.I., Simón M.R. (2002). Biological control of seedling blight of wheat caused by *Fusarium graminearum* with beneficial rhizosphere microorganisms. World J. Microbiol. Biotechnol..

[15] Khemir E., Chekali S., Moretti A., Gharbi M.S., Allagui M.B., Gargouri S. (2020). Impacts of previous crops on inoculum of *Fusarium culmorum* in soil, and development of foot and root rot of durum wheat in Tunisia. Phytopathol. Mediterr..

[16] Bhaskara Reddy M.V., Arul J., Angers P., Couture L. (1999). Chitosan treatment of wheat seeds induces resistance to *Fusarium graminearum* and improves seed quality. J. Agric. Food Chem..

[17] Khan M.R., Fischer S., Egan D., Doohan F.M. (2006). Biological control of Fusarium seedling blight disease of wheat and barley. Phytopathology.

[18] Wisniewska H., Kowalczyk K. (2005). Resistance of cultivars and breeding lines of spring wheat to *Fusarium culmorum* and powdery mildew. J. Appl. Genet..

[19] Gharbi M.S., El Felah M. (2013). Les céréales en Tunisie: Plus d’un siècle de recherche variétale. Ann. L’INRAT.

[20] Giorgi F. (2007). Climate change hot-spots’. Adaptation to climate change: Development of a national strategy for agriculture, ecosystems and water resources in Tunisia 2005 to December. Geophys. Resour. Lett..

[21] Woo S.L., Ruocco M., Vinale F., Nigro M., Marra R., Lombardi N., Pascale A., Lanzuise S., Manganiello G., Lorito M. (2014). *Trichoderma*-based products and their widespread use in agriculture. Open Mycol. J..

[22] Awad-Allah E.F.A., Mohamed I.A.A., Allah S.F.A.A., Shams A.H., Elsokkary I.H. (2022). *Trichoderma* Species: An Overview of Current Status and Potential Applications for Sustainable Agriculture. Indian J. Agric. Res..

[23] Benítez T., Rincón A.M., Limón M.C., Codón A.C. (2004). Biocontrol mechanisms of *Trichoderma* strains. Int. Microbiol..

[24] Li N., Alfiky A., Wang W., Islam, Nourollahi K., Liu X., Kang S. (2018). Volatile Compound-Mediated Recognition and Inhibition between *Trichoderma* Biocontrol Agents and *Fusarium oxysporum*. Front. Microbiol..

[25] Mendoza-Mendoza A., Zaid R., Lawry R., Hermosa R., Monte E., Horwitz B.A., Mukherjee P.K. (2018). Molecular dialogues between *Trichoderma* and roots: Role of the fungal secretome. Fungal Biol. Rev..

[26] Halifu S., Deng X., Song X., Song R., Liang X. (2020). Inhibitory Mechanism of *Trichoderma virens* ZT05 on Rhizoctonia Solani. Plants.

[27] Rao Y., Zeng L., Jiang H., Mei L., Wang Y. (2022). *Trichoderma atroviride* LZ42 releases volatile organic compounds promoting plant growth and suppressing Fusarium wilt disease in tomato seedlings. BMC Microbiol..

[28] Kaur S., Samota M.K., Choudhary M., Pandey A.K., Sharma A., Thakur J. (2022). How do plants defend themselves against pathogens-Biochemical mechanisms and genetic interventions. Physiol. Mol. Biol. Plants.

[29] Sallam N.M.A., Eraky A.M.I., Sallam A. (2019). Effect of *Trichoderma* spp. on Fusarium wilt disease of tomato. Mol. Biol. Rep..

[30] Yuan M., Huang Y., Ge W., Jia Z., Song S., Zhang L., Huang Y. (2019). Involvement of jasmonic acid, ethylene and salicylic acid signaling pathways behind the systemic resistance induced by *Trichoderma longibrachiatum* H9 in cucumber. BMC Genom..

[31] Zhou C., Guo R., Ji S., Fan H., Wang J., Wang Y., Liu Z. (2020). Isolation of *Trichoderma* from forestry model base and the antifungal properties of isolate TpsT17 toward *Fusarium oxysporum*. Microbiol. Res..

[32] Ben Amira M., Lopez D., Mohamed A.T., Khouaja A., Chaar H., Fumanal B., Gousset-Dupont A., Bonhomme L., Label P., Goupil P. (2017). Beneficial effect of *Trichoderma harzianum* strain Ths97 in biocontrolling *Fusarium solani* causal agent of root rot disease in olive trees. Biol. Control..

[33] Kthiri Z., Ben Jabeur M., Machraoui M., Gargouri S., Hiba K., Hamada W. (2020). Coating seeds with *Trichoderma* strains promotes plant growth and enhance the systemic resistance against *Fusarium* crown rot in durum wheat. Egypt. J. Biol. Pest Control.

[34] Boamah S., Zhang S., Xu B., Li T., Calderón-Urrea A. (2021). *Trichoderma longibrachiatum* (TG1) Enhances Wheat Seedlings Tolerance to Salt Stress and Resistance to *Fusarium pseudograminearum*. Front. Plant Sci..

[35] Kthiri Z., Ben Jabeur M., Harbaoui K., Karmous C., Chamekh Z., Chairi F., Serret M.D., Araus J.L., Hamada W. (2021). Comparative Performances of Beneficial Microorganisms on the Induction of Durum Wheat Tolerance to Fusarium Head Blight. Microorganisms.

[36] Bissett J. (1991). A revision of the genus *Trichoderma*. II. Infrageneric classification. J. Bot..

[37] Chaverri P., Castlebury L.A., Samuels G.J., Geiser D.M. (2002). Multilocus Phylogenetic Structure within the *Trichoderma harzianum/Hypocrea lixii* Complex. Mol. Phylogenetics Evol..

[38] Chaverri P., Branco-Rocha F., Jaklitsch W., Gazis R., Degenkolb T., Samuels G.J. (2015). Systematics of the *Trichoderma harzianum* species complex and the re-identification of commercial biocontrol strains. Mycologia.

[39] Zadoks J.C., Chang T.T., Konzak C.F. (1974). A decimal code for the growth stages of cereals. Weed Res..

[40] Elad Y., Chet I., Henis Y.A. (1981). A selective medium for improving quantitative isolation of *Trichoderma* spp. from soil. Phytoparasitica.

[41] Doyle J.J., Doyle J.L. (1987). A rapid DNA isolation procedure for small quantities of fresh leaf tissue. Phytochem. Bull..

[42] White T.J., Bruns T., Lee S.J., Taylor J., Innis M.A., Gelfand D.H., Sninsky J., White T.J. (1990). Amplification and Direct Sequencing of Fungal Ribosomal RNA Genes for Phylogenetics. PCR Protocols: A Guide to Methods and Applications.

[43] Carbone I., Kohn L.M. (1999). A method for designing primer sets for speciation studies in filamentous ascomycetes. Mycologia.

[44] Jaklitsch W.M., Komon M., Kubicek C.P., Druzhinina I.S. (2005). *Hypocrea voglmayrii* sp. *nov*. from the Austrian Alps represents a new phylogenetic clade in *Hypocrea*/*Trichoderma*. Mycologia.

[45] Liu Y.J., Whelen S., Hall B.D. (1999). Phylogenetic relationships among ascomycetes: Evidence from an RNA polymerse II subunit. Mol. Biol. Evol..

[46] Skidmore A.M., Dickinson C.H. (1976). Colony interactions and hyphal interference between Septoria Nodorum and phylloplane fungi. Trans. Br. Mycol. Soc..

[47] Dennis C., Webster J. (1971). Antagonistic properties of species-Groups of *Trichoderma*: II. Production of volatile antibiotics. Trans. Br. Mycol. Soc..

[48] Mahfooz M., Dwedi S., Bhatt A., Raghuvanshi S., Bhatt M., Agrawal P.K. (2017). Evaluation of Antifungal and Enzymatic Potential of Endophytic Fungi Isolated from Cupressus torulosa D. Don. Int. J. Curr. Microbiol. Appl. Sci..

[49] Bhardwaj A., Sharma D., Jadon N., Agrawal P.K. (2015). Antimicrobial and phytochemical screening of endophytic fungi isolated from spikes of *Pinus rouxburghii*. Arch. Clin. Microbiol..

[50] López A.C., Alvarenga A.E., Zapata P.D., Luna M.F., Villalba L.L. (2019). *Trichoderma* spp. from Misiones, Argentina: Effective fungi to promote plant growth of the regional crop *Ilex paraguariensis* St. Hil. Mycology.

[51] Roberts W.K., Selitrennikoff C.P. (1988). Plant and Bacterial Chitinases Differ in Antifungal Activity. J. Fish Biol..

[52] Coniglio R.O., Fonseca M.I., Villalba L.L., Zapata P.D. (2017). Screening of new secretory cellulases from different supernatants of white rot fungi from Misiones, Argentina. Mycology.

[53] Pikovskaya R.I. (1948). Mobilization of phosphorus in soil in connection with the vital activity of some microbial species. Mikrobiologya.

[54] Gordon S.A., Weber R.P. (1951). Colorimetric estimation of indole acetic acid. Plant Physiol..

[55] Noori M.S.S., Saud H.M. (2012). Potential plant growth-promoting activity of *Pseudomonas* sp. isolated from paddy soil in Malaysia as biocontrol agent. J. Plant Pathol. Microbiol..

[56] Castric K.F., Castric P.A. (1983). Method for rapid detection of cyanogenic bacteria. Appl. Environ. Microbiol..

[57] Rakh R.R., Raut L.S., Dalvi S.M., Manwar A.V. (2011). Biological control of *Sclerotium rolfsii*, causing stem rot of groundnut by *Pseudomonas* cf. *monteilii 9*. Recent Res. Sci. Technol..

[58] Cappucino J.C., Sherman N. (1992). Microbiology: A Laboratory Manual.

[59] Rezgui M., Ben Mechlia N., Bizid E., Kalboussi R., Hayouni R. (2000). Etude de La Stabilité du Rendement de Blé dur dans Différentes Régions de la Tunisie. L’Amélioration du Blé dur dans La Région Méditerranéenne: Nouveaux Défis. Options Méditerranéennes, Série A: Séminaires Méditerranéennes.

[60] Fernandez M.R., Chen Y. (2005). Pathogenicity of *Fusarium* species on diferent plant parts of spring wheat under controlled conditions. Plant Dis..

[61] Triveni S., Prasanna R., Shukla L., Saxena A.K. (2012). Evaluating the biochemical traits of novel *Trichoderma*-based biofilms for use as plant growth-promoting inoculants. Ann. Microbiol..

[62] Goulas Y., Cerovic Z.G., Cartelat A., Moya I. (2004). Dualex: A new instrument for field measurements of epidermal ultraviolet absorbance by chlorophyll fluorescence. Appl. Opt..

[63] Amira M.B., Mom R., Lopez D., Chaar H., Khouaja A., Pujade-Renaud V., Fumanal B., Gousset-Dupont A., Bronner G., Label P. (2018). MIP diversity from *Trichoderma*: Structural considerations and transcriptional modulation during mycoparasitic association with *Fusarium solani* olive trees. PLoS ONE.

[64] Livak K.J., Schmittgen T.D. (2001). Analysis of relative gene expression data using real-time quantitative PCR and the 2(-Delta Delta C(T)) Method. Methods.

[65] Levene H., Olkin I. (1960). Robust Tests for Equality of Variances. Contributions to Probability and Statistics.

[66] Fox J., Weisberg S. (2019). An R Companion to Applied Regression.

[67] Shapiro S.S., Wilk M.B. (1965). An Analysis of Variance Test for Normality (Complete Samples). Biometrika.

[68] R Core Team (2022). R: A Language and Environment for Statistical Computing.

[69] Welch B. (1951). On the Comparison of Several Mean Values: An Alternative Approach. Biometrika.

[70] Games P.A., Keselman H.J., Clinch J.J. (1979). Tests for Homogeneity of variance in factorial designs. Psychol. Bull..

[71] Kassambara A. (2022). rstatix: Pipe-Friendly Framework for Basic Statistical Tests.

[72] Carroll R.M., Nordholm L.A. (1975). Sampling Characteristics of Kelley’s epsilon and Hays’ omega. Educ. Psychol. Meas..

[73] Field F. (2013). Discovering Statistics Using IBM SPSS Statistics.

[74] Buchanan E.M., Gillenwaters A., Scofield J.E., Valentine K. (2019). MOTE (Measure of the Effect): Package to assist in effect size calculations and their confidence intervals. http://github.com/doomlab/MOTE.

[75] Kruskal W.H., Wallis W.A. (1952). Use of Ranks in One-Criterion Variance Analysis. J. Am. Stat. Assoc..

[76] Tomczak M., Tomczak E. (2014). The need to report effect size estimates revisited. An overview of some recommended measures of effect size. Trends Sport Sci..

[77] Mangiafico S. (2022). Rcompanion: Functions to Support Extension Education Program Evaluation.

[78] Dunn O.J. (1964). Multiple comparisons using rank sums. Technometrics.

[79] de Mendiburu F. (2021). Agricolae: Statistical Procedures for Agricultural Research.

[80] Royston P. (1982). An extension of Shapiro and Wilk’s W test for normality to large samples. Appl. Stat..

[81] Bartlett M.S. (1937). Properties of sufficiency and statistical tests. Proc. R. Soc. Lond. Ser. A.

[82] Kassambara A. (2023). ggpubr: ‘ggplot2′ Based Publication Ready Plots_.

[83] Wickham H., Averick M., Bryan J., Chang W., McGowan L.D.A., François R., Grolemund G., Hayes A., Henry L., Hester J. (2019). Welcome to the Tidyverse. J. Open Source Softw..

[84] Benjamini Y., Hochberg Y. (1995). Controlling the False Discovery Rate: A Practical and Powerful Approach to Multiple Testing. J. Royal Statistical Soc. Ser. B Methodol..

[85] Samuels G.J. (2006). *Trichoderma*: Systematics, the sexual state, and ecology. Phytopathology.

[86] Feitosa Y.B., Cruz-Magalhães V., Argolo-Filho R.C., De Souza J.T., Loguercio L.L. (2019). Characterization of genetic diversity on tropical *Trichoderma germplasm* by sequencing of rRNA internal transcribed spacers. BMC Res. Notes.

[87] Raja H.A., Miller A.N., Pearce C.J., Oberlies N.H. (2017). Fungal Identification Using Molecular Tools: A Primer for the Natural Products Research Community. J. Nat. Prod..

[88] Jaklitsch W.M., Voglmayr H. (2015). Biodiversity of *Trichoderma* (*Hypocreaceae*) in Southern Europe and Macaronesia. Stud. Mycol..

[89] Tekpinar A.D., Kalmer A. (2019). Utility of various molecular markers in fungal identification and phylogeny. Nova Hedwig..

[90] Fanelli F., Liuzzi V.C., Logrieco A.F., Altomare C. (2018). Genomic characterization of *Trichoderma atrobrunneum* (*T. harzianum* species complex) ITEM 908: Insight into the genetic endowment of a multi target biocontrol strain. BMC Genom..

[91] Gu X., Wang R., Sun Q., Wu B., Sun J.-Z. (2020). Four new species of *Trichoderma* in the Harzianum clade from northern China. Mycokeys.

[92] Gams W., Bisset J., Kubicek C.P., Harman G.E., Ondik K.L. (2007). Morphology and Identification of *Trichoderma*. Trichoderma and Gliocladium: Basic Biology, Taxonomy, and Genetics.

[93] Sadfi-Zouaoui N., Hannachi I., Rouaissi M., Hajlaoui M.R., Rubio M.B., Monte E., Boudabous A., Hermosa M.R. (2009). Biodiversity of *Trichoderma* strains in Tunisia. Can. J. Microbiol..

[94] Yangui I., Boutiti Z., Hlaiem S., Vettraino A.M., Vannini A., Ben Jamaâ M.L., Messaoud C. (2018). Identification and occurrence of *Trichoderma harzianum* associated with cork oak in Tunisia. Tunis. J. Plant Prot..

[95] Daami-Rreamdi M., Hibar K., Jabnoun-Khiareddine H., Ayed F., El-Mahjoub M. (2006). Effect of Two *Trichoderma* species on severity of potato tuber dry rot caused by Tunisian *Fusarium* complex. Int. J. Agric. Res..

[96] Mbazia A., Omri Ben Youssef N., Kharrat M. (2016). Tunisian isolates of *Trichoderma* spp. and *Bacillus subtilis* can control *Botrytis fabae* on faba bean. Biocontrol. Sci. Technol..

[97] El-Sharkawy H.H., Rashad Y.M., Ibrahim S.A. (2018). Biocontrol of stem rust disease of wheat using arbuscular mycorrhizal fungi and *Trichoderma* spp.. Physiol. Mol. Plant Pathol..

[98] Stummer B.E., Zhang Q., Zhang X., Warren R.A., Harvey P.R. (2020). Quantification of *Trichoderma afroharzianum*, *Trichoderma harzianum* and *Trichoderma gamsii* inoculants in soil, the wheat rhizosphere and in planta suppression of the crown rot pathogen *Fusarium pseudograminearum*. J. Appl. Microbiol..

[99] Junaid J.M., Dar N.A., Bhat T.A., Bhat A.H., Bhat M.A. (2013). Commercial biocontrol agents and their mechanism of action in the management of plant pathogens. Int. J. Mod. Plant Anim. Sci..

[100] Harman G.E., Obregón M.A., Samuels G.J., Lorito M. (2010). Changing models of biocontrol in the developing and developed world. Plant Dis..

[101] Stummer B.E., Zhang X., Yang H., Harvey P.R. (2022). Co-inoculation of *Trichoderma gamsii* A5MH and *Trichoderma harzianum* Tr906 in wheat suppresses in planta abundance of the crown rot pathogen *Fusarium pseudograminearum* and impacts the rhizosphere soil fungal microbiome. Biol. Control..

[102] Qiao M., Du X., Zhang Z., Xu J., Yu Z. (2018). Three new species of soil-inhabiting *Trichoderma* from southwest China. Mycokeys.

[103] Pfordt A., Schiwek S., Karlovsky P., Von Tiedemann A. (2020). *Trichoderma Afroharzianum* Ear Rot—A New Disease on Maize in Europe. Front. Agron..

[104] Cartelat A., Cerovic Z., Goulas Y., Meyer S., Lelarge C., Prioul J.-L., Barbottin A., Jeuffroy M.-H., Gate P., Agati G. (2005). Optically assessed contents of leaf polyphenolics and chlorophyll as indicators of nitrogen deficiency in wheat (*Triticum aestivum* L.). Field Crop. Res..

[105] Liu Y., Wang J., Xiao Y., Shi X., Zeng Y. (2021). Diversity Analysis of Chlorophyll, Flavonoid, Anthocyanin, and Nitrogen Balance Index of Tea Based on Dualex. Phyton.

[106] Guler N.S., Pehlivan N., Karaoglu S.A., Guzel S., Bozdeveci A. (2016). *Trichoderma atroviride* ID20G inoculation ameliorates drought stress-induced damages by improving antioxidant defense in maize seedlings. Acta Physiol. Plant..

[107] Harman G.E., Doni F., Khadka R.B., Uphoff N. (2021). Endophytic strains of *Trichoderma* increase plants’ photosynthetic capability. J. Appl. Microbiol. Biotechnol..

[108] Lee S., Yap M., Behringer G. (2016). Volatile organic compounds emitted by *Trichoderma* species mediate plant growth. Fungal Biol. Biotechnol..

[109] Nieto-Jacobo M.F., Steyaert J.M., Salazar-Badillo F.B., Nguyen D.V., Rostás M., Braithwaite M., De Souza J.T., Jimenez-Bremont J.F., Ohkura M., Stewart A. (2017). Environmental Growth Conditions of *Trichoderma* spp. Affects Indole Acetic Acid Derivatives, Volatile Organic Compounds, and Plant Growth Promotion. Front. Plant Sci..

[110] Yedidia I., Shoresh M. (2003). Concomitant induction of systemic resistance to *Pseudomonas syringae* pv. *lachrymans* in cucumber by *Trichoderma asperellum* (T-203) and accumulation of Phytoalexins. Appl. Environ. Microbiol..

[111] Jayapala N., Mallikarjunaiah N., Puttaswamy H. (2019). *Rhizobacteria Bacillus* spp. induce resistance against anthracnose disease in chili (*Capsicum annuum* L.) through activating host defense response. Egypt. J. Biol. Pest Control.

[112] Singh S.P., Keswani C., Singh S.P., Sansinenea E., Hoat T.X. (2021). *Trichoderma* spp. mediated induction of systemic defense response in brinjal against *Sclerotinia sclerotiorum*. Curr. Res. Microb. Sci..

[113] Rawat R., Tewari L. (2011). Effect of abiotic stress on phosphate solubilization by biocontrol fungus *Trichoderma* sp.. Curr. Microbiol..

[114] Sawant I.S., Wadkar P.N., Ghule S.B., Salunkhe V.P., Chavan V., Sawant S.D. (2020). Induction of systemic resistance in grapevines against powdery mildew by *Trichoderma asperelloides* strains. Australas. Plant Pathol..

[115] Marques A.P.G.C., Pires C., Moreira H., Rangel A.O., Castro P.M. (2010). Assessment of the plant growth promotion abilities of six bacterial isolates using Zea mays as indicator plant. Soil Biol. Biochem..

[116] Marina M.A.S., Silva-Flores M.A., Uresti-Rivera E.E., Castro-Longoria E., Herrera-Estrella A., Casas-Flores S. (2011). Colonization of *Arabidopsis* roots by *Trichoderma atroviride* promotes growth and enhances systemic disease resistance through jasmonic acid/ethylene and salicylic acid pathways. Eur. J. Plant Pathol..

[117] Kotasthane A., Agrawal T., Kushwah R., Rahatkar O.V. (2015). In-vitro antagonism of *Trichoderma* spp. against Sclerotium rolfsii and Rhizoctonia solani and their response towards growth of cucumber, bottle gourd and bitter gourd. Eur. J. Plant Pathol..

[118] Ng L.C., Ngadin A., Azhari M. (2015). Potential of *Trichoderma* spp. as Biological Control Agents Against Bakanae Pathogen (*Fusarium fujikuroi*) in Rice. Asian J. Plant Pathol..

[119] Poonguzhali S., Madhaiyan M., Sa T.M. (2008). Isolation and identification of phosphate solubilizing bacteria from Chinese cabbage and their effect on growth and phosphorus utilization of plants. J. Microbiol. Biotechnol..

[120] Majeed A., Abbasi M., Hameed S. (2015). Isolation and characterization of plant growth-promoting rhizobacteria from wheat rhizosphere and their effect on plant growth promotion. Front. Microbiol..

[121] Patten C., Glick B.R. (1996). Bacterial biosynthesis of indole-3-acetic acid. Can. J. Microbiol..

[122] Gilbert S., Xu J., Acosta K., Poulev A., Lebeis S., Lam E. (2018). Bacterial Production of Indole Related Compounds Reveals Their Role in Association Between Duckweeds and Endophytes. Front. Chem..

[123] Contreras-Cornejo H.A., Macias-Rodriguez L., Cortes-Penagos C. (2009). *Trichoderma virens*, a plant beneficial fungus, enhances biomass production and promotes lateral root growth through an auxin-dependent mechanism in *Arabidopsis*. Plant Physiol..

[124] Chowdappa P., Kumar S.P.M., Lakshmi M.J., Upreti K.K. (2013). Growth stimulation and induction of systemic resistance in tomatoagainst early and late blight by *Bacillus subtilis* OTPB1 or *Trichoderma harzianum* OTPB. Biol. Control.

[125] Zhao L., Zhang Y. (2015). Effects of phosphate solubilization and phytohormone production of *Trichoderma asperellum* Q1 on promoting cucumber growth under salt stress. J. Integr. Agric..

[126] Perrig D., Boiero M.L., Masciarelli O.A., Penna C., Ruiz O.A., Cassán F.D., Luna M.V. (2007). Plant-growth-promoting compounds produced by two agronomically important strains of Azospirillum brasilense, and implications for inoculant formulation. Appl. Microbiol. Biotechnol..

[127] Chiwocha S.D.S., Abrams S.R., Ambrose S.J., Cutler A.J., Loewen M., Ross A.R.S., Kermode A.R. (2003). A method for profiling classes of plant hormones and their metabolites using liquid chromatography-electrospray ionization tandem mass spectrometry: An analysis of hormone regulation of thermodormancy of lettuce (*Lactuca sativa* L.) seeds. Plant J..

[128] Goswami D., Thakker J.N., Dhandhukia P.C. (2015). Simultaneous detection and quantification of indole-3-acetic acid (IAA) and indole-3-butyric acid (IBA) produced by rhizobacteria from l-tryptophan (Trp) using HPTLC. J. Microbiol. Methods.

[129] Spaepen S., Vanderleyden J. (2011). Auxin and plant-microbe interactions. Cold Spring Harb. Perspect. Biol..

[130] Yedidia I., Srivastva A.K., Kapulnik Y. (2001). Effect of *Trichoderma harzianum* on microelement concentrations and increased growth of cucumber plants. Plant Soil.

[131] Fankem H., Nwaga D., Deubel A., Dieng L., Merbach W., Etoa F.X. (2006). Occurrence and functioning of phosphate solubilizing microorganisms from oil palm tree (*Elaeis guineensis*) rhizosphere in Cameroon. Afr. J. Biotechnol..

[132] Ghosh S.K., Banerjee S., Pal S. (2018). Encountering epidemic effects of leaf spot disease (*Alternaria brassicae*) on *Aloe vera* by fungal biocontrol agents in agri fields—An ecofriendly approach. PLoS ONE.

[133] El-Katatny M.S. (2004). Inorganic phosphate solubilisation by free or immobilized *Trichoderma harzianum* cells in comparison with some other soil fungi. Egypt. J. Biotechnol..

[134] Lalngaihawmi A., Bhattacharyya A. (2019). Study on the Different Modes of Action of Potential *Trichoderma* spp. from Banana Rhizosphere against *Fusarium oxysporum* f.sp. *cubense*. Int. J. Curr. Microbiol. Appl. Sci..

[135] Hassan S., Mathesius U. (2012). The role of flavonoids in root–rhizosphere signalling: Opportunities and challenges for improving plant–microbe interactions. J. Exp. Bot..

[136] Şesan T.E., Oancea A.O., Ştefan L.M., Mănoiu V.S., Ghiurea M., Răut I., Constantinescu-Aruxandei D., Toma A., Savin S., Bira A.F. (2020). Effects of foliar treatment with a *Trichoderma* plant biostimulant consortium on *Passiflora caerulea* L. yield and quality. Microorganisms.

[137] Harman G.E., Petzoldt R., Comis A., Chen J. (2004). Interactions Between *Trichoderma harzianum* Strain T22 and Maize Inbred Line Mo17 and Effects of These Interactions on Diseases Caused by *Pythium ultimum* and *Colletotrichum graminicola*. Phytopathology.

[138] Vinale F., Sivasithamparam K., Ghisalberti E.L., Marra R., Woo S.L., Lorito M. (2008). *Trichoderma*–plant–pathogen interactions. Soil Biol. Biochem..

[139] Garnica-Vergara A., Barrera-Ortiz S., Muñoz-Parra E., Raya-González J., Méndez-Bravo A., Macías-Rodríguez L., Ruiz-Herrera L.F., López-Bucio J. (2016). The volatile 6-pentyl-2H-pyran-2-one from *Trichoderma atroviride* regulates *Arabidopsis thaliana* root morphogenesis via auxin signaling and ETHYLENE INSENSITIVE 2 functioning. N. Phytol..

[140] Esparza-Reynoso S., Ruíz-Herrera L.F., Pelagio-Flores R., Macías-Rodríguez L.I., Martínez-Trujillo M., López-Coria M., Sánchez-Nieto S., Herrera-Estrella A., López-Bucio J. (2021). *Trichoderma atroviride*-emitted volatiles improve growth of *Arabidopsis* seedlings through modulation of sucrose transport and metabolism. Plant Cell Environ..

[141] Wonglom P., Ito S., Sunpapao A. (2020). Volatile organic compounds emitted from endophytic fungus *Trichoderma asperellum* T1 mediate antifungal activity, defense response and promote plant growth in lettuce (*Lactuca sativa*). Fungal Ecol..

[142] Lemfack M.C., Gohlke B.-O., Toguem S.M.T., Preissner S., Piechulla B., Preissner R. (2018). mVOC 2.0: A database of microbial volatiles. Nucleic Acids Res..

[143] Guo Y., Jud W., Ghirardo A., Antritter F., Benz J.P., Schnitzler J., Rosenkranz M. (2020). Sniffing fungi—Phenotyping of volatile chemical diversity in *Trichoderma* species. N. Phytol..

[144] Moya P., Girotti J.R., Toledo A.V., Sisterna M.N. (2018). Antifungal activity of *Trichoderma* VOCs against Pyrenophora teres, the causal agent of barley net blotch. J. Plant Prot. Res..

[145] Malinich E.A., Wang K., Mukherjee P.K., Kolomiets M., Kenerley C.M. (2019). Differential expression analysis of *Trichoderma virens* RNA reveals a dynamic transcriptome during colonization of Zea mays roots. BMC Genom..

[146] Zhang Y., Xiao J., Yang K., Wang Y., Tian Y., Liang Z. (2022). Transcriptomic and metabonomic insights into the biocontrol mechanism of *Trichoderma asperellum* M45a against watermelon Fusarium wilt. PLoS ONE.

[147] Zapparata A., Baroncelli R., Durling M.B., Kubicek C.P., Karlsson M., Vannacci G., Sarrocco S. (2021). Fungal cross-talk: An integrated approach to study distance communication. Fungal Genet. Biol..

[148] Seidl V., Marchetti M., Schandl R., Allmaier G., Kubicek C.P. (2006). Epl1, the major secreted protein of Hypocrea atroviridis on glucose, is a member of a strongly conserved protein family comprising plant defense response elicitors. FEBS J..

[149] Djonović S., Vargas W.A., Kolomiets M.V., Horndeski M., Wiest A., Kenerley C.M. (2007). A Proteinaceous Elicitor Sm1 from the Beneficial Fungus *Trichoderma virens* Is Required for Induced Systemic Resistance in Maize. Plant Physiol..

[150] Palmieri M.C., Perazzolli M., Matafora V., Moretto M., Bachi A., Pertot I. (2012). Proteomic analysis of grapevine resistance induced by *Trichoderma harzianum* T39 reveals specific defence pathways activated against downy mildew. J. Exp. Bot..

[151] Gadaleta A., Colasuonno P., Giove S.L., Blanco A., Giancaspro A. (2019). Map-based cloning of QFhb.mgb-2A identifies a WAK2 gene responsible for Fusarium Head Blight resistance in wheat. Sci. Rep..

[152] Chen T., Xiao J., Xu J., Wan W., Qin B., Cao A., Chen W., Xing L., Du C., Gao X. (2016). Two members of TaRLK family confer powdery mildew resistance in common wheat. BMC Plant Biol..

[153] Dmochowska-Boguta M., Kloc Y., Zielezinski A., Werecki P., Nadolska-Orczyk A., Karlowski W.M., Orczyk W. (2020). TaWAK6 encoding wall-associated kinase is involved in wheat resistance to leaf rust similar to adult plant resistance. PLoS ONE.

[154] Guo F., Wu T., Shen F., Xu G., Qi H., Zhang Z. (2021). The cysteine-rich receptor-like kinase TaCRK3 contributes to defense against *Rhizoctonia cerealis* in wheat. J. Exp. Bot..

[155] Rubio M.B., de Alba A.E.M., Nicolás C., Monte E., Hermosa R. (2019). Early Root Transcriptomic Changes in Wheat Seedlings Colonized by *Trichoderma harzianum* Under Different Inorganic Nitrogen Supplies. Front. Microbiol..

[156] Bienert G.P., Chaumont F. (2014). Aquaporin-facilitated transmembrane diffusion of hydrogen peroxide. Biochim. Biophys. Acta (BBA) Gen. Subj..

[157] Li G., Chen T., Zhang Z., Li B., Tian S. (2020). Roles of Aquaporins in Plant-Pathogen Interaction. Plants.

[158] Safdarpour F., Khodakaramain G. (2018). Endophytic bacteria suppress bacterial wilt of tomato. Caused by *Ralstonia solanacearum* and Activate defense–related metabolites. Biol. J. Microorg..

[159] Almagro L., Gómez Ros L.V., Belchi-Navarro S., Bru R., Ros Barceló A., Pedreño M.A. (2009). Class III peroxidases in plant defence reactions. J. Exp. Bot..

[160] Ali S., Ganai B.A., Kamili A.N., Bhat A.A., Mir Z.A., Bhat J.A., Tyagi A., Islam S.T., Mushtaq M., Yadav P. (2018). Pathogenesis-related proteins and peptides as promising tools for engineering plants with multiple stress tolerance. Microbiol. Res..

[161] Harman G.E., Howell C.R., Viterbo A., Chet I., Lorito M. (2004). *Trichoderma* species—Opportunistic, avirulent plant symbionts. Nat. Rev. Genet..

[162] Shoresh M., Harman G.E., Mastouri F. (2010). Induced systemic resistance and plant responses to fungal biocontrol agents. Annu. Rev. Phytopathol..

[163] Gomes E.V., Ulhoa C.J., Cardoza R.E., Silva R.N., Gutiérrez S. (2017). Involvement of *Trichoderma harzianum* Epl-1 Protein in the Regulation of Botrytis Virulence- and Tomato Defense-Related Genes. Front. Plant Sci..

[164] Pei Y., Li X., Zhu Y., Ge X., Sun Y., Liu N., Jia Y., Li F., Hou Y. (2019). GhABP19, a Novel Germin-Like Protein From Gossypium hirsutum, Plays an Important Role in the Regulation of Resistance to Verticillium and Fusarium Wilt Pathogens. Front. Plant Sci..

[165] Mathys J., De Cremer K., Timmermans P., Van Kerckhove S., Lievens B., Vanhaecke M., Cammue B.P.A., De Coninck B. (2012). Genome-Wide Characterization of ISR Induced in Arabidopsis thaliana by *Trichoderma hamatum* T382 Against Botrytis cinerea Infection. Front. Plant Sci..

[166] Rubio M.B., Quijada N.M., Pérez E., Domínguez S., Monte E., Hermosa R. (2014). Identifying Beneficial Qualities of *Trichoderma parareesei* for Plants. Appl. Environ. Microbiol..

[167] Martinez C., Blanc F., Le Claire E., Besnard O., Nicole M., Baccou J.-C. (2001). Salicylic Acid and Ethylene Pathways Are Differentially Activated in Melon Cotyledons by Active or Heat-Denatured Cellulase from *Trichoderma longibrachiatum*. Plant Physiol..

[168] Korolev N., David D.R., Elad Y. (2008). The role of phytohormones in basal resistance and *Trichoderma*-induced systemic resistance to *Botrytis cinerea* in *Arabidopsis thaliana*. BioControl.

[169] Medina M.H.J., Gagnon H., Piché Y., Ocampo J.A., Garcıa G.J.M., Vierheilig H. (2003). Root colonization by arbuscular mycorrhizal fungi is affected by the salicylic acid content of the plant. Plant Sci..

[170] Alonso-Ramírez A., Poveda J., Martín I., Hermosa R., Monte E., Nicolás C. (2014). Salicylic acid prevents *Trichoderma harzianum* from entering the vascular system of roots. Mol. Plant Pathol..

[171] Peleg-Grossman S., Golani Y., Kaye Y., Melamed-Book N., Levine A. (2009). NPR1 Protein Regulates Pathogenic and Symbiotic Interactions between Rhizobium and Legumes and Non-Legumes. PLoS ONE.

[172] Lopez-Raez J.A., Verhage A., Fernandez I., García J.M., Azcon-Aguilar C., Flors V., Pozo M.J. (2010). Hormonal and transcriptional profiles highlight common and differential host responses to arbuscular mycorrhizal fungi and the regulation of the oxylipin pathway. J. Exp. Bot..

[173] Viterbo A., Landau U., Kim S., Chernin L., Chet I. (2010). Characterization of ACC deaminase from the biocontrol and plant growth-promoting agent *Trichoderma asperellum* T203. FEMS Microbiol. Lett..

[174] Jaroszuk-Ściseł J., Tyśkiewicz R., Nowak A. (2019). Phytohormones (auxin, gibberellin) and ACC deaminase in vitro synthesized by the mycoparasitic *Trichoderma* DEMTkZ3A0 strain and changes in the level of auxin and plant resistance markers in wheat seedlings inoculated with this strain conidia. Int. J. Mol. Sci..

[175] Król P., Igielski R., Pollmann S., Kępczyńska E. (2015). Priming of seeds with methyl jasmonate induced resistance to hemibiotroph *Fusarium oxysporum* f. sp. *lycopersici* in tomato via 12-oxo-phytodienoic acid, salicylic acid, and flavonol accumulation. J. Plant Physiol..

[176] Sahu R., Sharaff M., Pradhan M., Sethi A., Bandyopadhyay T., Mishra V.K., Chand R., Chowdhury A.K., Joshi A.K., Pandey S.P. (2016). Elucidation of defense-related signaling responses to spot blotch infection in bread wheat (*Triticum aestivum* L.). Plant J..

[177] Singh U.B., Malviya D., Singh S., Kumar M., Sahu P.K., Singh H.V., Kumar S., Roy M., Imran M., Rai J.P. (2019). *Trichoderma harzianum*- and Methyl Jasmonate-Induced Resistance to Bipolaris sorokiniana Through Enhanced Phenylpropanoid Activities in Bread Wheat (*Triticum aestivum* L.). Front. Microbiol..

[178] Kepczynska E., Kepczynski J. (2005). Inhibitory effect of metyl jasmonates on developmentof phythopathogen *Alternaria alternata* (Fr.) Keissl. and its reversal by ethephon and ACC. Acta Physiol. Plant.

[179] Kepczynska E., Król P. (2012). The phytohormone methyl jasmonate as an activator of induced resistance against the necrotroph *Alternaria porri* f.sp. *solani* in tomato plants. J. Plant Interact..

[180] Švecová E., Proietti S., Caruso C., Colla G., Crinò P. (2013). Antifungal activity of Vitex agnus-castus extract against Pythium ultimum in tomato. Crop. Prot..

[181] Samolski I., Rincón A.M., Pinzón L.M., Viterbo A., Monte E. (2012). The qid74 gene from *Trichoderma harzianum* has a role in root architecture and plant biofertilization. Microbiology.

[182] Ruano-Rosa D., Prieto P., Rincón A.M., Gómez-Rodríguez M.V., Valderrama R., Barroso J.B., Mercado-Blanco J. (2016). Fate of *Trichoderma harzianum* in the olive rhizosphere: Time course of the root colonization process and interaction with the fungal pathogen Verticillium dahliae. Biocontrol.

[183] Basińska-Barczak A., Błaszczyk L., Szentner K. (2020). Plant Cell Wall Changes in Common Wheat Roots as a Result of Their Interaction with Beneficial Fungi of *Trichoderma*. Cells.

[184] Ganuza M., Pastor N., Boccolini M., Erazo J., Palacios S., Oddino C., Reynoso M., Rovera M., Torres A. (2019). Evaluating the impact of the biocontrol agent *Trichoderma harzianum* ITEM 3636 on indigenous microbial communities from field soils. J. Appl. Microbiol..

[185] Illescas M., Rubio M.B., Hernández-Ruiz V., Morán-Diez M.E., de Alba A.E.M., Nicolás C., Monte E., Hermosa R. (2020). Effect of Inorganic N Top Dressing and *Trichoderma harzianum* Seed-Inoculation on Crop Yield and the Shaping of Root Microbial Communities of Wheat Plants Cultivated Under High Basal N Fertilization. Front. Plant Sci..

[186] Friedl M.A., Druzhinina I.S. (2012). Taxon-specific metagenomics of *Trichoderma* reveals a narrow community of opportunistic species that regulate each other’s development. Microbiology.

[187] Singh A., Lasek-Nesselquist E., Chaturvedi V., Chaturvedi S. (2018). *Trichoderma polysporum* selectively inhibits white nose syndrome fungal pathogen *Pseudogymmnoascus destructans* amidst soil microbes. Microbiome.

[188] Martínez-Medina A., Fernández I., Sánchez-Guzmán M.J., Jung S.C., Pascual J.A., Pozo M.J. (2013). Deciphering the hormonal signalling network behind the systemic resistance induced by *Trichoderma harzianum* in tomato. Front. Plant Sci..

[189] Leonetti P., Zonno M.C., Molinari S., Altomare C. (2017). Induction of SA-signaling pathway and ethylene biosynthesis in *Trichoderma harzianum*-treated tomato plants after infection of the root-knot nematode Meloidogyne incognita. Plant Cell Rep..

[190] Van Wees S.C., De Swart E.A., Van Pelt J.A., Van Loon L.C., Pieterse C.M. (2000). Enhancement of induced disease resistance by simultaneous activation of salicylate- and jasmonate-dependent defense pathways in *Arabidopsis thaliana*. Proc. Natl. Acad. Sci. USA.

[191] Mur L.A., Kenton P., Atzorn R., Miersch O., Wasternack C. (2006). The Outcomes of Concentration-Specific Interactions between Salicylate and Jasmonate Signaling Include Synergy, Antagonism, and Oxidative Stress Leading to Cell Death. Plant Physiol..

[192] Illescas M., Pedrero-Méndez A., Pitorini-Bovolini M., Hermosa R., Monte E. (2021). Phytohormone Production Profiles in *Trichoderma* Species and Their Relationship to Wheat Plant Responses to Water Stress. Pathogens.

[193] Alfano G., Ivey M.L.L., Cakir C., Bos J.I.B., Miller S.A., Ma S., Kamoun S., Hoitink H.A.J., Alfano M.L.L.I.G., Uddin M.N. (2007). Systemic Modulation of Gene Expression in Tomato by *Trichoderma hamatum* 382. Phytopathology.

[194] Segarra G., Jáuregui O., Casanova E., Trillas I. (2006). Simultaneous quantitative LC–ESI-MS/MS analyses of salicylic acid and jasmonic acid in crude extracts of Cucumis sativus under biotic stress. Phytochemistry.

[195] Attaran E., Major I.T., Cruz J.A., Rosa B.A., Koo A., Chen J., Kramer D., He S.Y., Howe G.A. (2014). Temporal Dynamics of Growth and Photosynthesis Suppression in Response to Jasmonate Signaling. Plant Physiol..

[196] Huot B., Yao J., Montgomery B.L., He S.Y. (2014). Growth–Defense Tradeoffs in Plants: A Balancing Act to Optimize Fitness. Mol. Plant.

