# Supplementary material for: Assessment of Tunisian Trichoderma Isolates on Wheat Seed Germination, Seedling Growth and Fusarium Seedling Blight Suppression
Source: Microorganisms. 2023 Jun 6;11(6):1512. doi: 10.3390/microorganisms11061512 (PMC10303082; doi:10.3390/microorganisms11061512)
Supplement: Supplementary file 1 [file microorganisms-11-01512-s001.zip › Supplemental Fig S1.pdf]

Figure S1

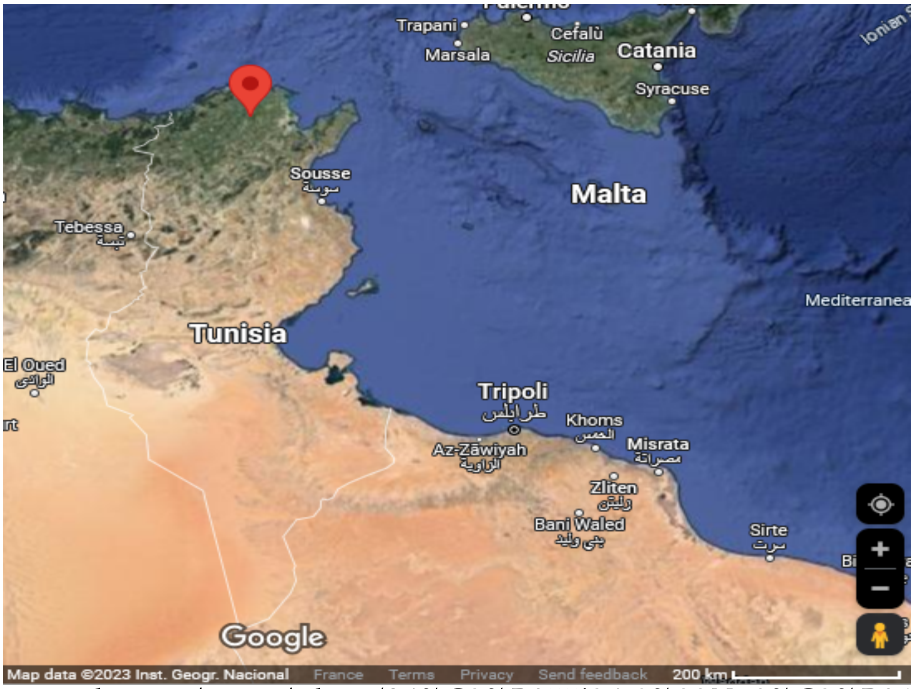

https://www.google.com/maps/place/36%C2%B055'26.8%22N+9%C2%B041'56.7%22E/@35.3806522,4.7332687,1262953m/data=!3m1!1e3!4m4!3m3!8m2!3d36.924096!4d9.699089?hl=en

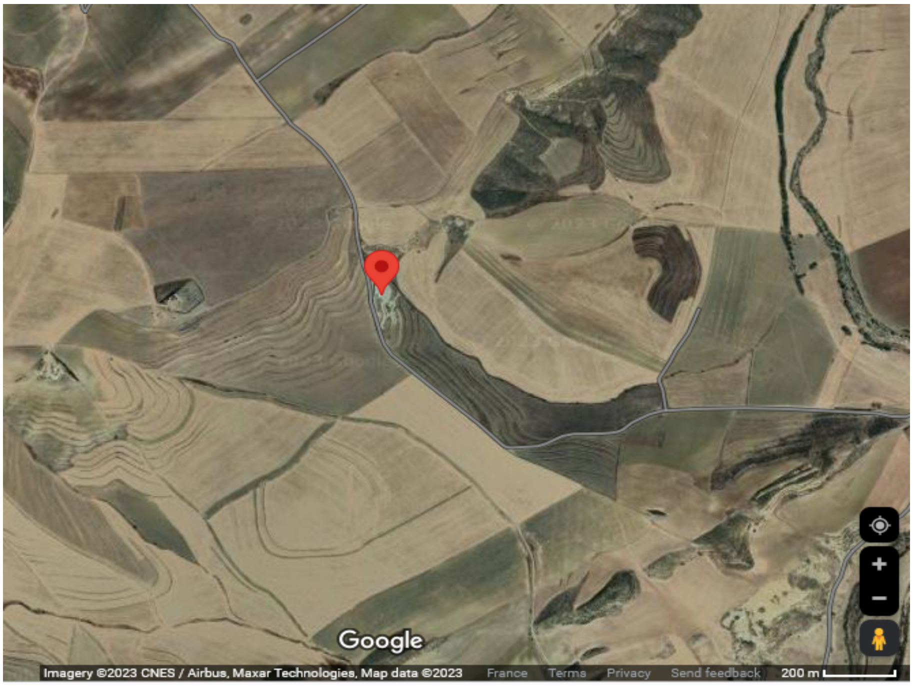

https://www.google.com/maps/place/36%C2%B055'26.8%22N+9%C2%B041'56.7%22E/@36.9233412,9.6896476,2419m/data=!3m1!1e3!4m4!3m3!8m2!3d36.924096!4d9.699089?hl=en

Supplementary Figure S1. Tunisian site of origin of the *Trichoderma* strains used in this work.
