# Supplementary material for: Assessment of Tunisian Trichoderma Isolates on Wheat Seed Germination, Seedling Growth and Fusarium Seedling Blight Suppression
Source: Microorganisms. 2023 Jun 6;11(6):1512. doi: 10.3390/microorganisms11061512 (PMC10303082; doi:10.3390/microorganisms11061512)
Supplement: Supplementary file 1 [file microorganisms-11-01512-s001.zip › Supplemental Fig S2.pdf]

Figure S2

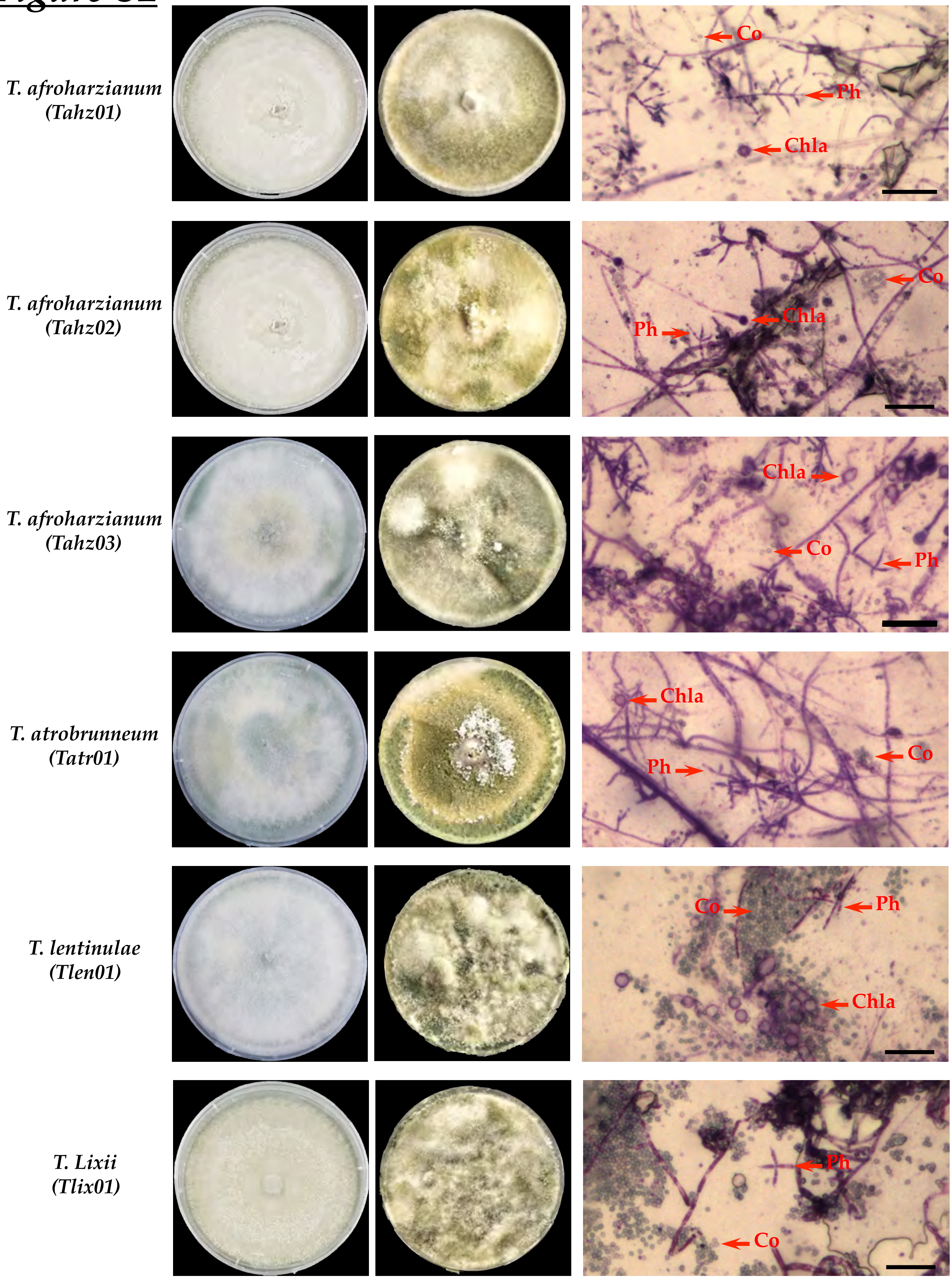

Supplementary Figure S2. Macroscopic and microscopic aspects of the *Trichoderma* isolates. Isolates were grown on PDA medium after 10 and 20 days of incubation at 28 °C. Morphological characteristics were visualized using optical microscopy (×100), after coloration with toluidine blue (0.5 % w/v). The scal bars represent 20 µm in all panels. Co, Conidiospores ; Chla, Chlamydospores; Ph, Phyalides.
