# Supplementary material for: Assessment of Tunisian Trichoderma Isolates on Wheat Seed Germination, Seedling Growth and Fusarium Seedling Blight Suppression
Source: Microorganisms. 2023 Jun 6;11(6):1512. doi: 10.3390/microorganisms11061512 (PMC10303082; doi:10.3390/microorganisms11061512)
Supplement: Supplementary file 1 [file microorganisms-11-01512-s001.zip › Supplemental Fig S3.pdf]

Figure S3.

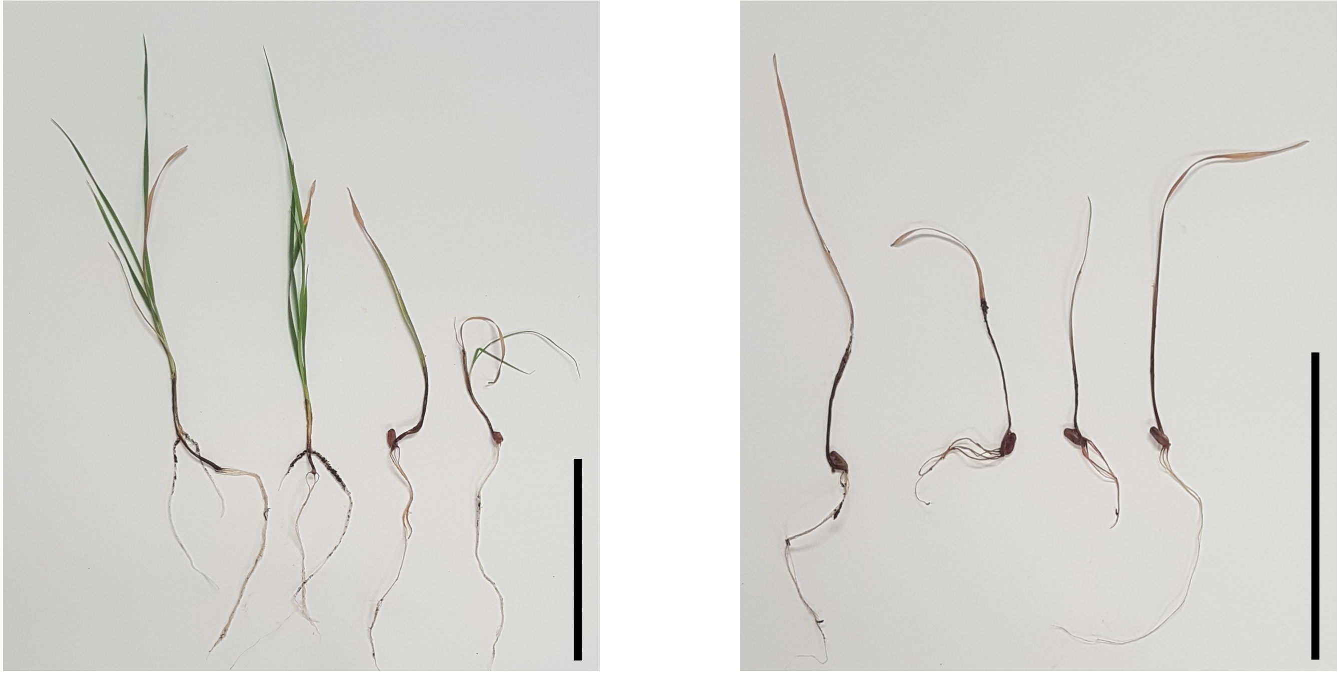

Supplementary Figure S3. Root and leaves disease symptoms on wheat (Khiar variety) caused by *Fusarium culmorum*, 21 days after seed inoculation. The scal bars represent 10 cm.
