# Supplementary material for: Assessment of Tunisian Trichoderma Isolates on Wheat Seed Germination, Seedling Growth and Fusarium Seedling Blight Suppression
Source: Microorganisms. 2023 Jun 6;11(6):1512. doi: 10.3390/microorganisms11061512 (PMC10303082; doi:10.3390/microorganisms11061512)
Supplement: Supplementary file 1 [file microorganisms-11-01512-s001.zip › Supplemental Fig S4.pdf]

Figure S4.

Supplementary Figure S4. Bioprotection effect of the six *Trichoderma* isolates against *F. culmorum* infestation on wheat (Khiar variety) in axenic environment, 7 days after seed co-inoculation of fungal protagonists. The white scale bars represent 5 cm. The box on the left is a top view, the right box to the down view.

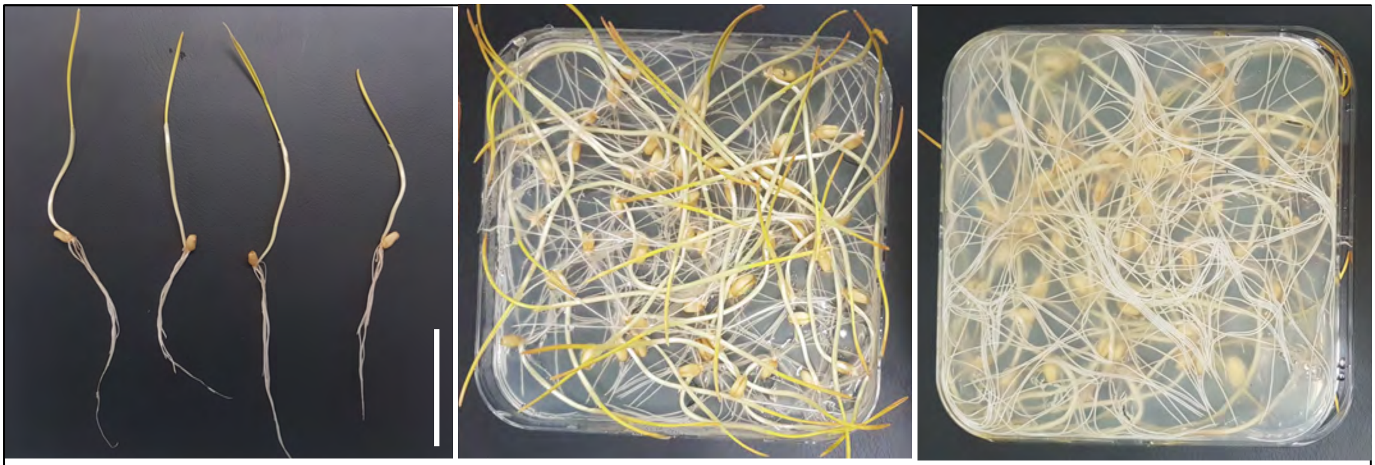

Untreated seedlings

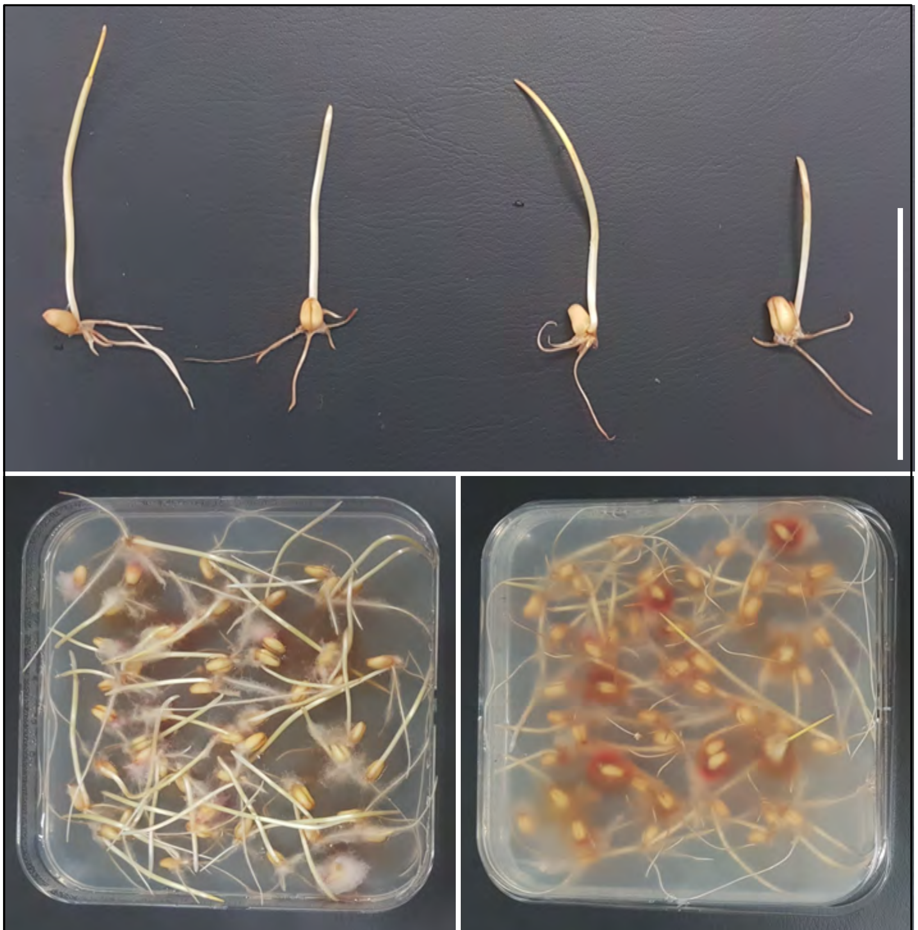

F. culmorum

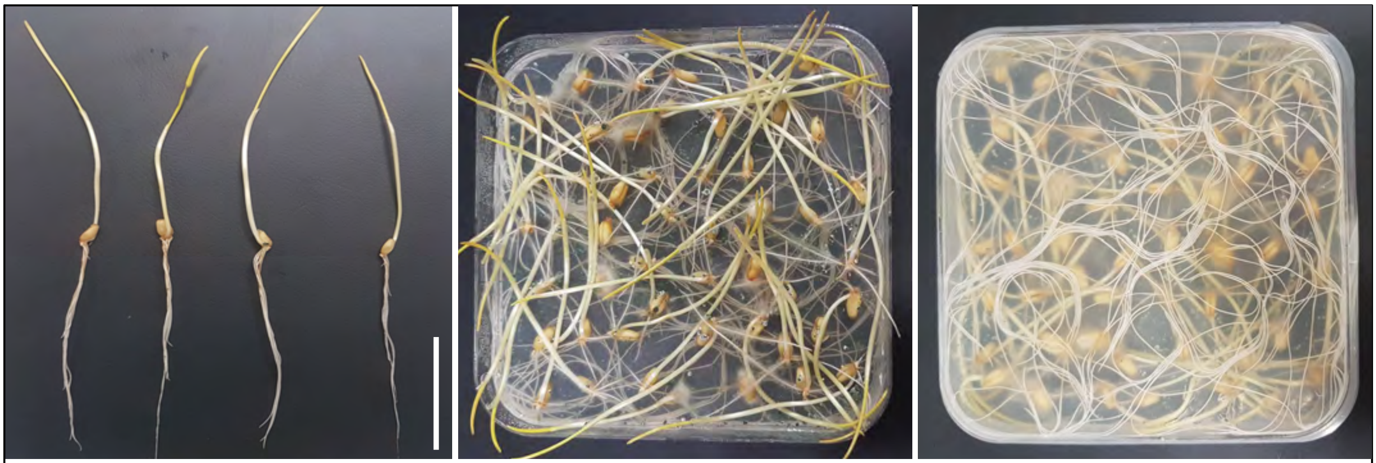

T. afroharzianum (*Tahz01*)

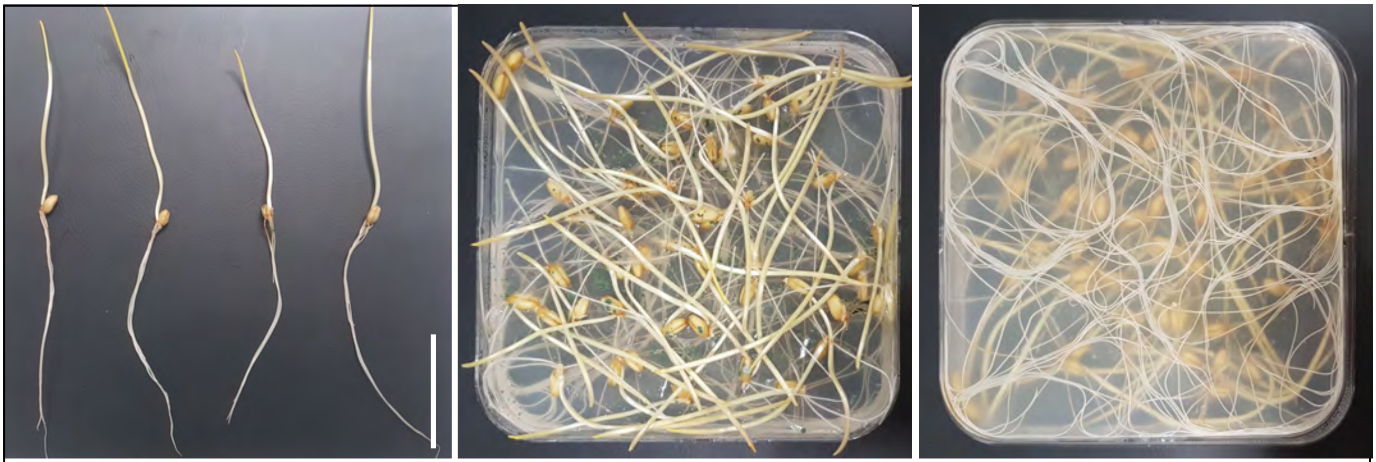

T. afroharzianum (*Tahz02*)

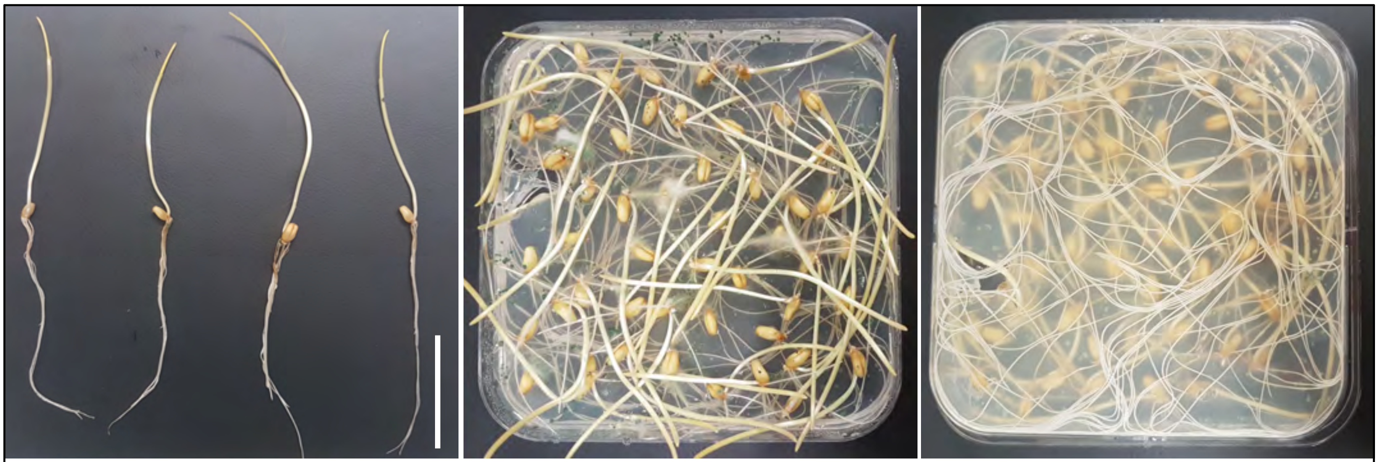

T. afroharzianum(*Tahz03*)

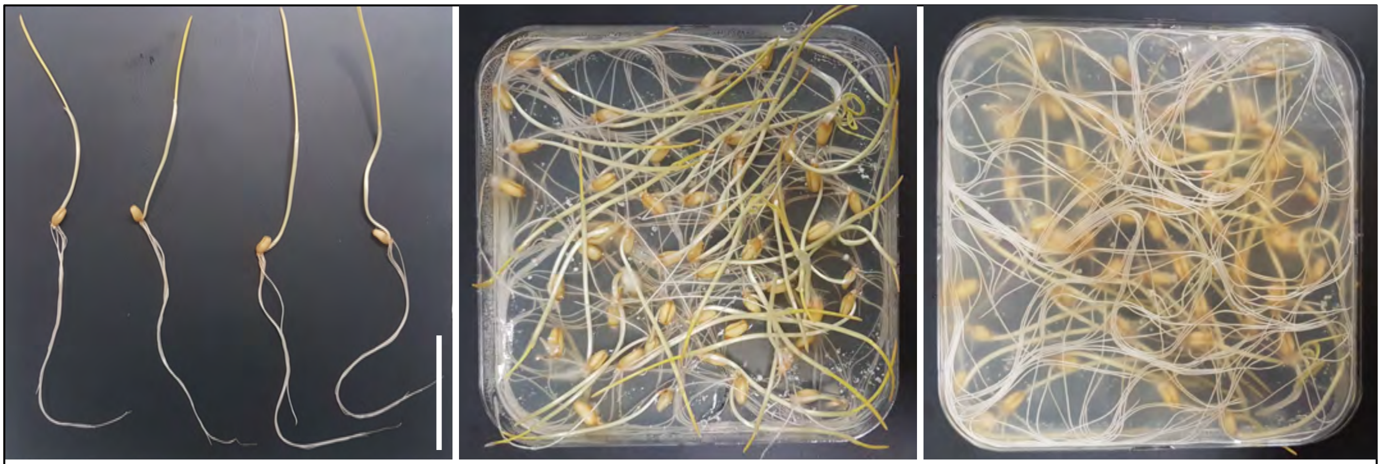

T. atrobrunneum (*Tatr03*)

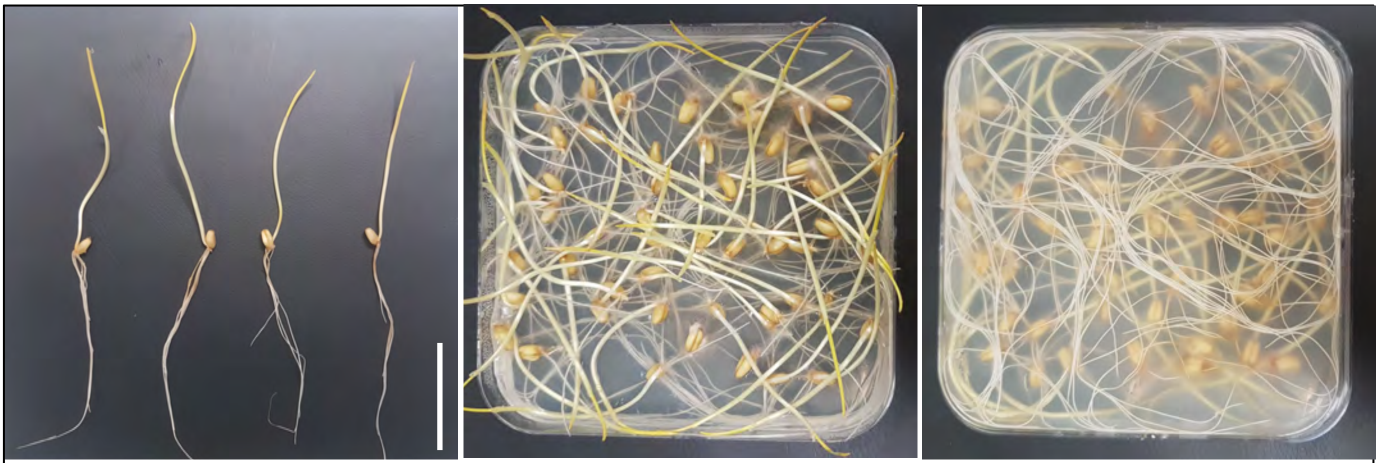

T. Lentinulae (*Tlen01*)

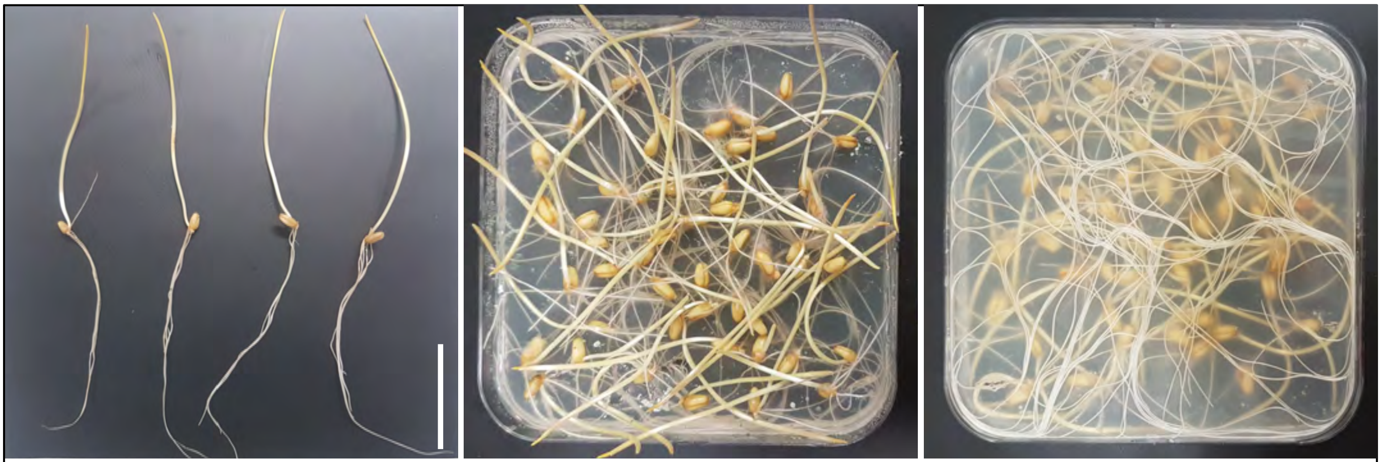

T. Lixii (*Tlix01*)
