# Supplementary material for: Assessment of Tunisian Trichoderma Isolates on Wheat Seed Germination, Seedling Growth and Fusarium Seedling Blight Suppression
Source: Microorganisms. 2023 Jun 6;11(6):1512. doi: 10.3390/microorganisms11061512 (PMC10303082; doi:10.3390/microorganisms11061512)
Supplement: Supplementary file 1 [file microorganisms-11-01512-s001.zip › Supplemental Fig S5.pdf]

Figure S5.

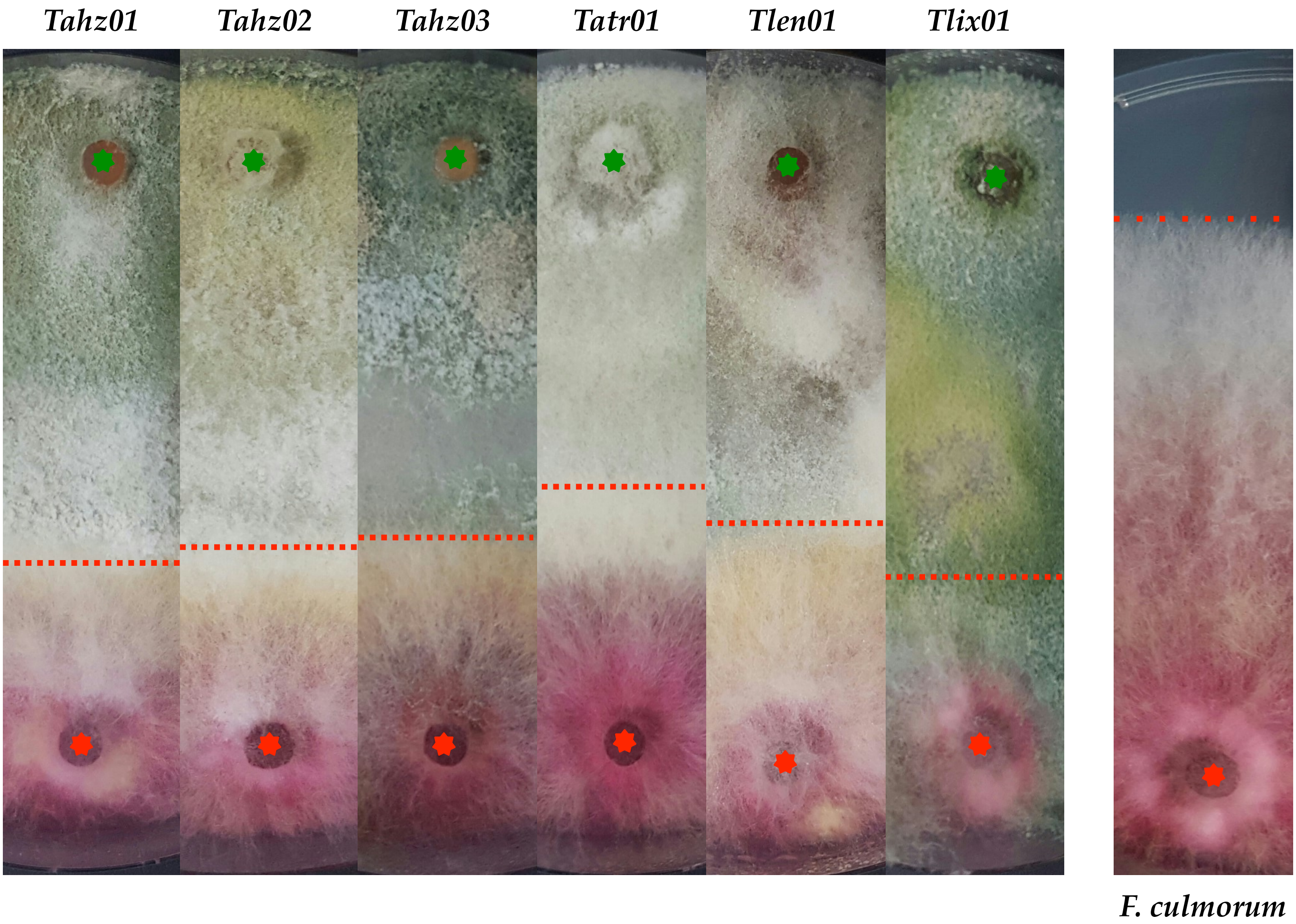

Supplementary Figure S5. Direct mycelial confrontation between *Trichoderma* isolates and *F. culmorum*. Development of the mycoparasitic reaction of the 6 *Trichoderma* isolates (★) against *F. culmorum* (★). Mycelial was grown on PDA medium for 6 days at 27 °C. The growth periphery of *F. culmorum* is indicated by a dotted red line.
