# Supplementary material for: Assessment of Tunisian Trichoderma Isolates on Wheat Seed Germination, Seedling Growth and Fusarium Seedling Blight Suppression
Source: Microorganisms. 2023 Jun 6;11(6):1512. doi: 10.3390/microorganisms11061512 (PMC10303082; doi:10.3390/microorganisms11061512)
Supplement: Supplementary file 1 [file microorganisms-11-01512-s001.zip › Supplemental Fig S6.pdf]

Figure S6.

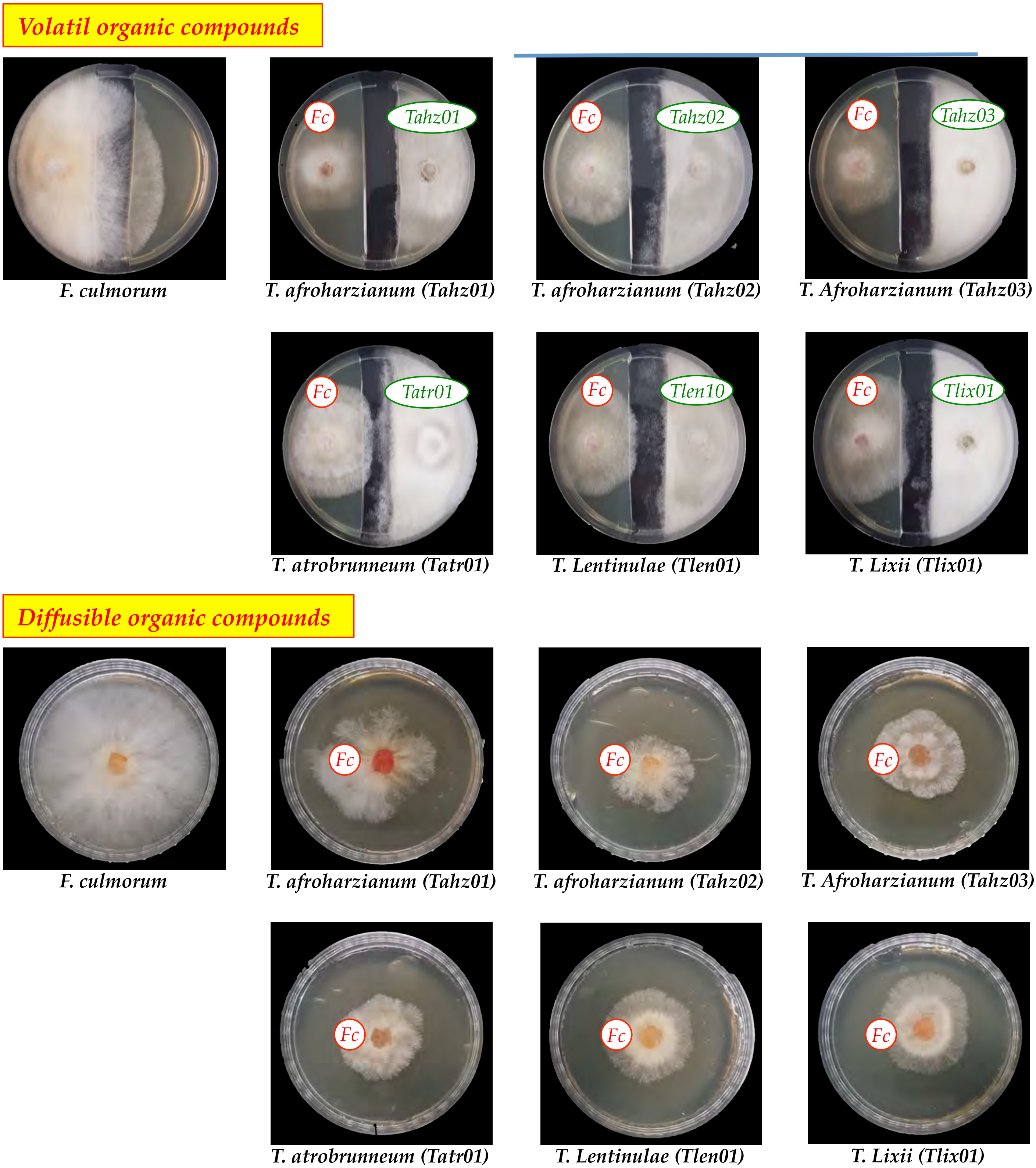

Supplementary Figure S6. Indirect antagonistic activity related to emission of volatile and diffusible organic compounds by *Trichoderma* isolates against *F. culmorum* on PDA medium after 7 days of confrontation (28 °C). Photographic examples for one biological repetition from six ones.
