# Supplementary material for: Assessment of Tunisian Trichoderma Isolates on Wheat Seed Germination, Seedling Growth and Fusarium Seedling Blight Suppression
Source: Microorganisms. 2023 Jun 6;11(6):1512. doi: 10.3390/microorganisms11061512 (PMC10303082; doi:10.3390/microorganisms11061512)
Supplement: Supplementary file 1 [file microorganisms-11-01512-s001.zip › Supplemental Fig S7.pdf]

Figure S7.

	Trichoderma		*Fusarium*	
**Isolates**	**Leaves**	**Roots**	**Leaves**	**Roots**
**Control**	**-**	**-**	**-**	**-**
Tahz01	nd	+	nd	+
Tahz02	nd	+	nd	+
Tahz03	nd	+	nd	+
Tatr01	nd	+	nd	+
Tlen01	nd	+	nd	+
Tlix01	nd	+	nd	+
F. Culmorum	-	-	+	+

Supplementary Figure S7. Qualitative molecular characterisation of the presence of *Trichoderma* or *F. culmorum* isolates in the aerial and underground parts of wheat plants 21 days after seed treatment with *Trichoderma* and/or *F. culmorum* isolates. Characterisation carried out on samples used for molecular analysis of plant defences. Presence detected by PCR through the amplification of the gene encoding for *Tubulin,* and whose primers are specific to the *Trichoderma* or *Fusarium* genera. The PCR analyses were conducted on cDNA diluted 1:5 and 1:20. "+", significant PCR signal; "-", no PCR signal; nd: not detectable (signal not analysable because it is mired in an amplification background noise).
