# Supplementary material for: Assessment of Tunisian Trichoderma Isolates on Wheat Seed Germination, Seedling Growth and Fusarium Seedling Blight Suppression
Source: Microorganisms. 2023 Jun 6;11(6):1512. doi: 10.3390/microorganisms11061512 (PMC10303082; doi:10.3390/microorganisms11061512)
Supplement: Supplementary file 1 [file microorganisms-11-01512-s001.zip › Supplemental Table S1.pdf]

Supplementary Table S1. Primers used for *q*PCR amplification of the wheat defense-encoding genes.

Function	Gene, full name	Acronym	primer (For/Rev)	PCR efficiency
**Receptors**	*Receptor Like Kinase*	*LRR-RLK*	TGCTGCATTCCTGATCAAGG / GGTGAAATTTGGTGTTGCAC	2,01
	Receptor-like cytoplasmic kinase	*RLCK1*	GCGAGGGTGAGATTCTGGAG / CTGGCTTAGTTGGGGTGAGG	1,81
	Wall Asssociated Kinase 2	*WAK2A*	GGTATCGTGCTACTGGAGCTC / CTCCCATGGTAGGCCTGTTA	2,15
	Wall Asssociated Kinase 6	*WAK6*	AGAACCTCTCGTCGCACTTC / CCTTAGCCTGCCAAGCTCTT	2,14
**Transduction network**	*Mitogen-activated protein kinase 3*	*MAPK3*	TACATGAGGCACCTGCCGCAGT / GGTTCAACTCCAGGGCTTCGTTG	2,32
	Cysteine-rich receptor-like kinase	*CERK3*	AGGTTCGAGACTTTCATGTTCT / GTGGAGCAGCTACAGATATAGC	2,18
**ROS generation and diffusion**	*NADPH oxidase (subunit D)*	*RbohD*	ACCACCAGACCAGACAGAC / TGGTTGGATAGGAGGCGTAG	2,31
	NADPH oxidase (subunit F)	*RbohF*	TGGCACCCCTTCTCAATTAC / CTCTCGTGTCCAGTCACCAA	1,97
	Aquaporin	*AQP1*	TGGTCAGACCACTGGATCTTC / TGGCATCTTCTTTGCAGCAG	2,07
**Antioxidative complexe**	*Glutathion peroxidase*	*GPX*	GCGGTGACACCAACATCAAC / GTCCAGGTTCTCCAGGTTGG	1,99
	Catalase	*CAT*	CACCTGGTGGAGAAGATCGC / TCACCTCGAAGAAGCCCTTG	1,96
	Superoxide dismutase	*SOD*	CGATAGCCAGATTCCTTTGACT / GAAACCAGCGACCTACAACG	2,11
	Glutathion S-transferase	*GST*	TGCTCCGCAAGTACAAGACG / ATGAGCGGGTTGATGAGGC	2,01
**Phenylpropanoid pathway**	*Phenylalanine ammonia lyase*	*PAL*	GGCCCACAAATCGAAGTTATTC / GGAAGTTGCCACCGTGGAT	2,09
	Chalcone synthase	*CHS*	GCGCCTGCGTACTCTTCATC / CCTCGGCGGAGCGTTT	1,92
	caffeic acid O-methyltransferase	*COMT*	ACGTCGACATGATCATGCTC / ACTCGATGGCAAATGCGTTG	2,07
	dihydroflavonol 4-réductase	*DFR*	TACGACCAGGACAACTGGAG / GGGATGATGCTGATGAAGTC	1,95
	Flavonol synthase1	*FLS*	GGTGTTCAAGGACGGATGCTGGTA / CGGCCACGACATCCGCGTCTT	2,16
	Polyphenol oxidase	*PPO*	GTCCGGACGAACAAGAAGAA / GGCGATGCTTCACTTGGTAT	2,09
	Benzoate 4-monoxygenase	*B4MO*	CGCCGAGGACATGCACGAGTG / GAAGACGCCGTGCCAGAAGGG	2,09
**Secondary metabolisms**	*Cinnamyl alcohol dehydrogenase*	*CAD1*	AGATACCGCTTCGTCATCG / GAATCGCACGCACCAACC	1,97
	Cinnamyl Coenzyme Reductase 3	*CCR3*	CTGTCGGCTAGTTAATTCTATG / ATATGATCGCCAACCAACC	2,11
	glucan synthase-like 22 (callose synthase)	*GSL22*	GTCTGGGGGCGATTTTA / GCTGTATGACGAACTGCTGA	2,18
	Farnesyl pyrophosphate synthase (sesquiterpenes)	*FPS1*	TCCTTCAACGCCACTTCAGAGGAA / TGTCTTGTATTGCACAATGCGCCG	2,12
	β-glucosidase (cyanogenic glycosides)	*BGD2*	ATGGTATATGGATCCACTGACGT / TATGGGATTCCCGTTACGCTCAC	1,87
**Pathogenesis Related Proteins**	*Pathogenesis Related protein 1a (PR-1)*	*PR1a*	GAATGCAGACGCCCAAGCTA / GCACGGGCAGCGTTGT	1,78
	Pathogenesis Related protein 1b (PR-1)	*PR1b*	ACTACGACTACGGGTCCAACA / TCGTAGTTGCAGGTGATGAAG	2,26
	Glucanase (PR-2)	*GLU*	TCCTGGGTTCAGAACAATGTCC / TTGATGTTGACAGCCGGGTAGT	1,96
	Chitinase basic, class 1 (PR-3)	*CHTb*	GCCACGTCCCCACCATACTAT / CCGGCAAGATCGTAGTTGGA	2,25
	Chitinase (PR-4)	*CHT2*	GGGTGGACCTGCTGAACAAT / AGAACCATATCGCCGTCTTGA	2,01
	Peroxydase (PR-9)	*POX2*	TGCTTTGTCCAAGGCTGTGA / GACCCGCGTTTTGTTCCA	1,97
	Defensine (PR-12)	*DEF*	ATGTCCGTGCCTTTTGCTA / CCAAACTACCGAGTCCCCG	1,93
	Lipid transfer protein (PR-14)	*LTP*	ACGTAGGTACTCCTCTCGCTGT / GTTGATCGACCACTTCTTCTCA	2,19
	Oxalate oxidase (PR-15)	*OXO*	GCCAGAACCCCGGTATCG / GGTGGGTTGGAGCTGAAGAG	2,14
	Germine-like (PR-16)	*GLP*	AACAAAGGTGATGTGTTCGTCTTC / GAGCCGGTCTATTGTATTCTTTTCC	1,83
**Phytohormonal status**	*Non Expresseur of Pathogenesis Related -1 (SA syhtesis)*	*NPR1*	GCTTGTCAGGATGCTGCTC / GAACAGTATAACCTCTTGGGTTTC	1,78
	Isochorismate synthase (SA syhtesis)	*ICS*	AGAAATGAGGACGACGAGTTTGAC / CCAAGTAGTGCTGATCTAATCCCAA	2,04
	Enhanced Disease Susceptibility 1 (SA syhtesis)	*EDS1*	CATCATCCCACGAATACCGCTGG / CCTAGGATTGAGCTAGTGCATCC	2,01
	Aminocyclopropane (ACC) synthase 1 (Et syhtesis)	*ACS1*	CTCGAAACCCTGCAAGAGATAG / CTCGAAACCCTGCAAGAGATAG	1,89
	Aminocyclopropane (ACC) oxydase (Et syhtesis)	*ACO*	TGTCCATCGCCTCCTTCTA / CGAACACGAACCTTGGGTAT	1,79
	Ethylene response Factor 3 (Et pathway)	*ERF3*	GCAATCGGGCAAAGCAAAC / CGACTCAGAACGAACCACGA	2,02
	Ethylene Insensitive3-Like1 (Et pathway)	*EIL1b*	GGCTCAACAACCTGGATTTT / CCCTGTCCAAAGAAACCCT	2,03
	Ethylene signaling pathway (Et pathway)	*PIE*	GGAGCCACCAGTCCGTATGA / CACCCGGCAGAGGTATTCAA	1,87
	Jasmonate resistant 1 (JA synthesis)	*JAR1b*	TCTTCTGTCGCCCTTCTTTG / GCGGAACAAAATCAAATTGC	1,78
	Lipoxygénase (JA synthesis)	*LOX*	GGGCACCAAGGAGTACAAGGA / GCTCGTGATGGTGTGGATGA	2,18
	Allene oxide synthase 1 (JA synthesis)	*AOS*	ACCGTGTTCAACAGCTACGG / AGCGCCTCTATCGTCACCTT	1,92
	Allene oxide synthase 2 (JA synthesis)	*AOS*	AGGCCGGAGAGAAGTTCCAC / CCGACTTGGTCAGCTCCATC	2,05
	Phosphoinositide-speci!c phospholipase (lipid signaling)	*PLC*	CCCGGAGAAAGCACGACAT / CCCAAGCTCATCCGAGACA	1,87
**Housekeeping genes**	*Actine*	*ACT*	AGTGGAGGTTCTACCATGTTTCCT / CACTGTATTTCCTTTCAGGTGGTG	2,52
	RNaseL inhibitor-like	*Rli*	TTGAGCAACTCATGGACCAG / GCTTTCCAAGGCACAAACAT	2,09
	Heteogeneous nuclear ribonucleoprotein (hn-RNP-Q)	*RPN*	TCACCTTCGCCAAGCTCAGAACTA / AGTTGAACTTGCCCGAAAC	2,05

Function	Gene, full name	Acronym	primer (For/Rev)	PCR efficiency
**Receptors**	*Receptor Like Kinase*	*LRR-RLK*	TGCTGCATTCCTGATCAAGG / GGTGAAATTTGGTGTTGCAC	2,01
	Receptor-like cytoplasmic kinase	*RLCK1*	GCGAGGGTGAGATTCTGGAG / CTGGCTTAGTTGGGGTGAGG	1,81
	Wall Asssociated Kinase 2	*WAK2A*	GGTATCGTGCTACTGGAGCTC / CTCCCATGGTAGGCCTGTTA	2,15
	Wall Asssociated Kinase 6	*WAK6*	AGAACCTCTCGTCGCACTTC / CCTTAGCCTGCCAAGCTCTT	2,14
**Transduction network**	*Mitogen-activated protein kinase 3*	*MAPK3*	TACATGAGGCACCTGCCGCAGT / GGTTCAACTCCAGGGCTTCGTTG	2,32
	Cysteine-rich receptor-like kinase	*CERK3*	AGGTTCGAGACTTTCATGTTCT / GTGGAGCAGCTACAGATATAGC	2,18
**ROS generation and diffusion**	*NADPH oxidase (subunit D)*	*RbohD*	ACCACCAGACCAGACAGAC / TGGTTGGATAGGAGGCGTAG	2,31
	NADPH oxidase (subunit F)	*RbohF*	TGGCACCCCTTCTCAATTAC / CTCTCGTGTCCAGTCACCAA	1,97
	Aquaporin	*AQP1*	TGGTCAGACCACTGGATCTTC / TGGCATCTTCTTTGCAGCAG	2,07
**Antioxidative complexe**	*Glutathion peroxidase*	*GPX*	GCGGTGACACCAACATCAAC / GTCCAGGTTCTCCAGGTTGG	1,99
	Catalase	*CAT*	CACCTGGTGGAGAAGATCGC / TCACCTCGAAGAAGCCCTTG	1,96
	Superoxide dismutase	*SOD*	CGATAGCCAGATTCCTTTGACT / GAAACCAGCGACCTACAACG	2,11
	Glutathion S-transferase	*GST*	TGCTCCGCAAGTACAAGACG / ATGAGCGGGTTGATGAGGC	2,01
**Phenylpropanoid pathway**	*Phenylalanine ammonia lyase*	*PAL*	GGCCCACAAATCGAAGTTATTC / GGAAGTTGCCACCGTGGAT	2,09
	Chalcone synthase	*CHS*	GCGCCTGCGTACTCTTCATC / CCTCGGCGGAGCGTTT	1,92
	caffeic acid O-methyltransferase	*COMT*	ACGTCGACATGATCATGCTC / ACTCGATGGCAAATGCGTTG	2,07
	dihydroflavonol 4-réductase	*DFR*	TACGACCAGGACAACTGGAG / GGGATGATGCTGATGAAGTC	1,95
	Flavonol synthase1	*FLS*	GGTGTTCAAGGACGGATGCTGGTA / CGGCCACGACATCCGCGTCTT	2,16
	Polyphenol oxidase	*PPO*	GTCCGGACGAACAAGAAGAA / GGCGATGCTTCACTTGGTAT	2,09
	Benzoate 4-monoxygenase	*B4MO*	CGCCGAGGACATGCACGAGTG / GAAGACGCCGTGCCAGAAGGG	2,09
**Secondary metabolisms**	*Cinnamyl alcohol dehydrogenase*	*CAD1*	AGATACCGCTTCGTCATCG / GAATCGCACGCACCAACC	1,97
	Cinnamyl Coenzyme Reductase 3	*CCR3*	CTGTCGGCTAGTTAATTCTATG / ATATGATCGCCAACCAACC	2,11
	glucan synthase-like 22 (callose synthase)	*GSL22*	GTCTGGGGGCGATTTTA / GCTGTATGACGAACTGCTGA	2,18
	Farnesyl pyrophosphate synthase (sesquiterpenes)	*FPS1*	TCCTTCAACGCCACTTCAGAGGAA / TGTCTTGTATTGCACAATGCGCCG	2,12
	β-glucosidase (cyanogenic glycosides)	*BGD2*	ATGGTATATGGATCCACTGACGT / TATGGGATTCCCGTTACGCTCAC	1,87
**Pathogenesis Related Proteins**	*Pathogenesis Related protein 1a (PR-1)*	*PR1a*	GAATGCAGACGCCCAAGCTA / GCACGGGCAGCGTTGT	1,78
	Pathogenesis Related protein 1b (PR-1)	*PR1b*	ACTACGACTACGGGTCCAACA / TCGTAGTTGCAGGTGATGAAG	2,26
	Glucanase (PR-2)	*GLU*	TCCTGGGTTCAGAACAATGTCC / TTGATGTTGACAGCCGGGTAGT	1,96
	Chitinase basic, class 1 (PR-3)	*CHTb*	GCCACGTCCCCACCATACTAT / CCGGCAAGATCGTAGTTGGA	2,25
	Chitinase (PR-4)	*CHT2*	GGGTGGACCTGCTGAACAAT / AGAACCATATCGCCGTCTTGA	2,01
	Peroxydase (PR-9)	*POX2*	TGCTTTGTCCAAGGCTGTGA / GACCCGCGTTTTGTTCCA	1,97
	Defensine (PR-12)	*DEF*	ATGTCCGTGCCTTTTGCTA / CCAAACTACCGAGTCCCCG	1,93
	Lipid transfer protein (PR-14)	*LTP*	ACGTAGGTACTCCTCTCGCTGT / GTTGATCGACCACTTCTTCTCA	2,19
	Oxalate oxidase (PR-15)	*OXO*	GCCAGAACCCCGGTATCG / GGTGGGTTGGAGCTGAAGAG	2,14
	Germine-like (PR-16)	*GLP*	AACAAAGGTGATGTGTTCGTCTTC / GAGCCGGTCTATTGTATTCTTTTCC	1,83
**Phytohormonal status**	*Non Expresseur of Pathogenesis Related -1 (SA syhtesis)*	*NPR1*	GCTTGTCAGGATGCTGCTC / GAACAGTATAACCTCTTGGGTTTC	1,78
	Isochorismate synthase (SA syhtesis)	*ICS*	AGAAATGAGGACGACGAGTTTGAC / CCAAGTAGTGCTGATCTAATCCCAA	2,04
	Enhanced Disease Susceptibility 1 (SA syhtesis)	*EDS1*	CATCATCCCACGAATACCGCTGG / CCTAGGATTGAGCTAGTGCATCC	2,01
	Aminocyclopropane (ACC) synthase 1 (Et syhtesis)	*ACS1*	CTCGAAACCCTGCAAGAGATAG / CTCGAAACCCTGCAAGAGATAG	1,89
	Aminocyclopropane (ACC) oxydase (Et syhtesis)	*ACO*	TGTCCATCGCCTCCTTCTA / CGAACACGAACCTTGGGTAT	1,79
	Ethylene response Factor 3 (Et pathway)	*ERF3*	GCAATCGGGCAAAGCAAAC / CGACTCAGAACGAACCACGA	2,02
	Ethylene Insensitive3-Like1 (Et pathway)	*EIL1b*	GGCTCAACAACCTGGATTTT / CCCTGTCCAAAGAAACCCT	2,03
	Ethylene signaling pathway (Et pathway)	*PIE*	GGAGCCACCAGTCCGTATGA / CACCCGGCAGAGGTATTCAA	1,87
	Jasmonate resistant 1 (JA synthesis)	*JAR1b*	TCTTCTGTCGCCCTTCTTTG / GCGGAACAAAATCAAATTGC	1,78
	Lipoxygénase (JA synthesis)	*LOX*	GGGCACCAAGGAGTACAAGGA / GCTCGTGATGGTGTGGATGA	2,18
	Allene oxide synthase 1 (JA synthesis)	*AOS*	ACCGTGTTCAACAGCTACGG / AGCGCCTCTATCGTCACCTT	1,92
	Allene oxide synthase 2 (JA synthesis)	*AOS*	AGGCCGGAGAGAAGTTCCAC / CCGACTTGGTCAGCTCCATC	2,05
	Phosphoinositide-speci!c phospholipase (lipid signaling)	*PLC*	CCCGGAGAAAGCACGACAT / CCCAAGCTCATCCGAGACA	1,87
**Housekeeping genes**	*Actine*	*ACT*	AGTGGAGGTTCTACCATGTTTCCT / CACTGTATTTCCTTTCAGGTGGTG	2,52
	RNaseL inhibitor-like	*Rli*	TTGAGCAACTCATGGACCAG / GCTTTCCAAGGCACAAACAT	2,09
	Heteogeneous nuclear ribonucleoprotein (hn-RNP-Q)	*RPN*	TCACCTTCGCCAAGCTCAGAACTA / AGTTGAACTTGCCCGAAAC	2,05
