# Supplementary material for: Assessment of Tunisian Trichoderma Isolates on Wheat Seed Germination, Seedling Growth and Fusarium Seedling Blight Suppression
Source: Microorganisms. 2023 Jun 6;11(6):1512. doi: 10.3390/microorganisms11061512 (PMC10303082; doi:10.3390/microorganisms11061512)
Supplement: Supplementary file 1 [file microorganisms-11-01512-s001.zip › Supplemental Table S2.pdf]

Supplementary Table S2. Statistical analyses of the transcript accumulation induced by *Trichoderma* isolates and/or *Fusarium culmorum* (Values highlighted in green are significant; Student's *T* realized after that normality and homoskedascity were tested; error level of 5 %; n = 3)

Leaves

Gene	Tahz01	Tahz02	Tahz03	Tatr01	Tlen01	Tlix01	Tahz01 + Fc	Tahz02 + Fc	Tahz03 + Fc	Tatr01 + Fc	Tlen01 + Fc	Tlix01 + Fc
ACO	0,057	0,22	0,23	0,057	0,081	0,32	0,053	0,036	0,019	0,8	0,82	0,025
ACS1	0,034	0,067	0,11	0,07	0,084	0,079	0,089	0,061	0,059	0,083	0,067	0,052
AOS1	0,027	0,029	0,57	0,07	0,9	0,77	0,08	0,025	0,15	0,14	0,15	0,22
AOS2	0,011	0,048	0,13	0,18	0,064	0,16	0,025	0,15	0,15	0,054	0,059	0,25
AQP1	0,11	0,07	0,05	0,034	0,2	0,065	0,64	0,37	0,27	0,082	0,083	0,37
CAD	0,035	0,025	0,048	0,22	0,048	0,038	0,036	0,054	0,78	0,55	0,069	0,034
CAT1	0,073	0,063	0,57	0,54	0,027	0,046	0,11	0,9	0,096	0,95	0,43	0,13
CCR3	0,028	0,0063	0,22	0,18	0,012	0,011	0,092	0,09	0,016	0,88	0,79	0,17
CHS	0,013	0,021	0,085	0,18	0,085	0,041	0,03	0,025	0,0048	0,03	0,25	0,014
CHTb	0,05	0,18	0,48	0,027	0,18	0,037	0,035	0,026	0,096	0,15	0,16	0,059
CHT2	0,71	0,07	0,29	0,025	0,095	0,054	0,028	0,13	0,061	0,82	0,82	0,92
COMT	0,088	0,07	0,19	0,23	0,11	0,13	0,0048	0,0049	0,061	0,058	0,035	0,037
CRK3	0,012	0,035	0,14	0,0069	0,037	0,052	0,51	0,22	0,15	0,18	0,24	0,039
DEF	0,05	0,18	0,085	0,05	0,026	0,034	0,68	0,63	0,16	0,069	0,069	0,5
DFR	0,28	0,012	0,037	0,06	0,54	0,013	0,51	0,25	0,23	0,82	0,12	0,31
ERF3	0,18	0,041	0,56	0,21	0,15	0,025	0,24	0,27	0,42	0,26	0,35	0,22
FLS1	0,03	0,029	0,58	0,06	0,055	0,97	0,03	0,12	0,068	0,089	0,19	0,42
FPS1	0,042	0,021	0,089	0,026	0,04	0,072	0,058	0,063	0,061	0,51	0,57	0,069
GLP	0,024	0,13	0,064	0,014	0,035	0,23	0,054	0,12	0,058	0,26	0,17	0,46
GLU	0,042	0,028	0,079	0,0071	0,23	0,05	0,014	0,11	0,03	0,083	0,083	0,13
GPX	0,015	0,11	0,035	0,036	0,013	0,021	0,078	0,11	0,13	0,91	0,73	0,24
GSL22	0,06	0,04	0,3	0,89	0,18	0,031	0,18	0,26	0,41	0,13	0,1	0,27
EndoGLU	0,096	0,016	0,22	0,042	0,11	0,062	0,03	0,068	0,043	0,63	0,82	0,035
ICS	0,041	0,14	0,028	0,1	0,053	0,035	0,09	0,15	0,37	0,82	0,37	0,41
LOX	0,035	0,011	0,33	0,49	0,18	0,084	0,12	0,12	0,58	0,32	0,12	0,67
LRR_RLK	0,052	0,041	0,11	0,035	0,15	0,06	0,26	0,13	0,15	0,15	0,2	0,11
LTP	0,2	0,37	0,052	0,07	0,28	0,025	0,025	0,025	0,017	0,0087	0,015	0,012
MAPK3	0,038	0,031	0,067	0,047	0,2	0,025	0,087	0,88	0,22	0,061	0,056	0,22
NPR1	0,0028	0,025	0,044	0,098	0,039	0,012	0,096	0,21	0,26	0,035	0,027	0,073
OXO	0,012	0,046	0,028	0,0063	0,2	0,0093	0,0049	0,29	0,13	0,22	0,19	0,13
PAL	0,07	0,06	0,079	0,13	0,63	0,23	0,063	0,058	0,016	0,12	0,063	0,023
PIE	0,38	0,28	0,1	0,44	0,04	0,22	0,061	0,067	0,069	0,17	0,15	0,4
PLC	0,35	0,038	0,11	0,53	0,057	0,3	0,43	0,092	0,43	0,1	0,58	0,14
POXa	0,01	0,027	0,036	0,0063	0,011	0,0069	0,016	0,083	0,11	0,53	0,2	0,073
POXb	0,0063	0,052	0,079	0,43	0,34	0,038	0,22	0,13	0,08	0,56	0,52	0,68
PPO	0,025	0,046	0,036	0,98	0,058	0,027	0,0092	0,22	0,27	0,21	0,098	0,054
PR1a	0,038	0,07	0,55	0,97	0,76	0,29	0,15	0,92	0,34	0,84	0,89	0,66
PR1b	0,052	0,038	0,2	0,0063	0,22	0,52	0,014	0,088	0,061	0,096	0,059	0,051
psyGST	0,063	0,16	0,1	0,066	0,3	0,79	0,034	0,15	0,031	0,069	0,016	0,06
RbohD	0,53	0,37	0,14	0,041	0,14	0,26	0,19	0,2	0,21	0,054	0,036	0,043
RbohF	0,13	0,045	0,084	0,065	0,078	0,042	0,11	0,37	0,12	0,09	0,088	0,096
RLCK	0,012	0,013	0,2	0,018	0,012	0,013	0,054	0,11	0,016	0,044	0,13	0,081
SOD	0,11	0,025	0,53	0,43	0,64	0,035	0,79	0,24	0,96	0,11	0,11	0,083
WAK2	0,026	0,0075	0,022	0,011	0,025	0,11	0,95	0,2	0,23	0,26	0,27	0,11
WAK6	0,13	0,29	0,39	0,041	0,04	0,94	0,4	0,1	0,45	0,15	0,3	0,069

Roots

Gene	Tahz01	Tahz02	Tahz03	Tatr01	Tlen01	Tlix01	Tahz01 + Fc	Tahz02 + Fc	Tahz03 + Fc	Tatr01 + Fc	Tlen01 + Fc	Tlix01 + Fc
ACO	0,085	0,09	0,049	0,064	0,16	0,098	0,61	0,96	0,27	0,034	0,054	0,96
ACS1	0,062	0,057	0,042	0,042	0,049	0,04	0,68	0,86	0,4	0,66	0,42	0,91
AOS1	0,07	0,042	0,07	0,075	0,027	0,051	0,58	0,24	0,43	0,098	0,81	0,41
AOS2	0,04	0,054	0,052	0,11	0,026	0,034	0,14	0,6	0,74	0,084	0,68	0,43
AQP1	0,027	0,054	0,041	0,059	0,025	0,042	0,97	0,079	0,47	0,26	0,032	0,61
CAD	0,18	0,053	0,13	0,035	0,015	0,011	0,061	0,06	0,71	0,26	0,78	0,057
CAT1	0,14	0,62	0,24	0,14	0,95	0,74	0,15	0,22	0,13	0,2	0,089	0,63
CCR3	0,18	0,067	0,11	0,54	0,15	0,066	0,57	0,97	0,5	0,38	0,68	0,77
CHS	0,11	0,46	0,51	0,72	0,04	0,46	0,24	0,24	0,36	0,36	0,74	0,24
CHTb	0,2	0,42	0,57	0,95	0,07	0,13	0,92	0,96	0,96	0,13	0,15	0,95
CHT2	0,48	0,18	0,3	0,17	0,53	0,035	0,045	0,054	0,043	0,044	0,48	0,035
COMT	0,25	0,44	0,18	0,12	0,54	0,26	0,27	0,44	0,22	0,25	0,21	0,15
CRK3	0,39	0,037	0,05	0,06	0,052	0,025	0,22	0,88	0,64	0,19	0,71	0,26
DEF	0,025	0,086	0,024	0,025	0,4	0,024	0,12	0,15	0,16	0,66	0,7	0,15
DFR	0,044	0,048	0,06	0,058	0,054	0,084	0,53	0,77	0,81	0,069	0,29	0,37
ERF3	0,2	0,34	0,26	0,28	0,021	0,077	0,41	0,41	0,29	0,061	0,32	0,44
FLS1	0,33	0,44	0,25	0,2	0,13	0,18	0,096	0,058	0,069	0,22	0,054	0,083
FPS1	0,18	0,42	0,83	0,9	0,4	0,053	0,99	0,036	0,16	0,069	0,26	0,12
GLP	0,14	0,057	0,025	0,025	0,05	0,025	0,31	0,96	0,88	0,16	0,81	0,22
GLU	0,062	0,086	0,13	0,033	0,035	0,11	0,47	0,47	0,99	0,2	0,41	0,8
GPX	0,13	0,085	0,1	0,18	0,23	0,075	0,061	0,28	0,11	0,054	0,15	0,099
GSL22	0,12	0,11	0,13	0,18	0,11	0,42	0,45	0,12	0,66	0,083	0,56	0,4
EndoGLU	0,1	0,095	0,45	0,31	0,035	0,049	0,07	0,082	0,29	0,088	0,8	0,48
ICS	0,037	0,016	0,061	0,13	0,18	0,0082	0,29	0,26	0,35	0,51	0,66	0,21
LOX	0,18	0,089	0,28	0,027	0,15	0,081	0,73	0,71	0,24	0,42	0,48	0,51
LRR_RLK	0,041	0,0063	0,0063	0,0063	0,025	0,0063	0,093	0,41	0,91	0,38	0,58	0,17
LTP	0,13	0,1	0,63	0,046	0,072	0,049	0,037	0,11	0,5	0,15	0,86	0,63
MAPK3	0,086	0,23	0,29	0,17	0,22	0,14	0,48	0,37	0,078	0,097	0,55	0,4
NPR1	0,22	0,18	0,46	0,063	0,25	0,052	0,95	0,48	0,17	0,82	0,17	0,37
OXO	0,085	0,054	0,085	0,048	0,035	0,044	0,56	0,74	0,98	0,15	0,68	0,82
PAL	0,18	0,029	0,025	0,035	0,44	0,037	0,035	0,032	0,031	0,46	0,27	0,03
PIE	0,2	0,048	0,031	0,04	0,21	0,031	0,2	0,51	0,39	0,62	0,84	0,82
PLC	0,085	0,042	0,041	0,041	0,038	0,013	0,13	0,11	0,67	0,22	0,65	0,082
POXa	0,067	0,049	0,1	0,37	0,14	0,056	0,096	0,48	0,26	0,035	0,22	0,096
POXb	0,05	0,049	0,062	0,1	0,34	0,11	0,27	0,75	0,37	0,045	0,15	0,57
PPO	0,29	0,47	0,72	0,47	0,42	0,43	0,098	0,11	0,089	0,21	0,32	0,12
PR1a	0,018	0,22	0,23	0,11	0,026	0,073	0,5	0,52	0,82	0,15	0,29	0,81
PR1b	0,035	0,025	0,029	0,026	0,15	0,079	0,22	0,22	0,25	0,086	0,83	0,21
psyGST	0,69	0,75	0,81	0,95	0,53	0,4	0,1	0,23	0,97	0,26	0,91	0,2
RbohD	0,17	0,2	0,2	0,16	0,4	0,056	0,11	0,12	0,53	0,92	0,58	0,69
RbohF	0,058	0,04	0,045	0,05	0,26	0,085	0,44	0,61	0,85	0,13	0,1	0,65
RLCK	0,57	0,17	0,15	0,19	0,48	0,2	0,47	0,2	0,12	0,7	0,98	0,27
SOD	0,17	0,2	0,38	0,21	0,69	0,81	0,054	0,11	0,92	0,18	0,86	0,093
WAK2	0,14	0,042	0,048	0,11	0,66	0,095	0,81	0,91	0,8	0,69	0,49	0,86
WAK6	0,37	0,23	0,084	0,66	0,97	0,66	0,048	0,11	0,5	0,017	0,13	0,12
